# Combined reversible switching of ECD and quenching of CPL with chiral fluorescent macrocycles†
†Electronic supplementary information (ESI) available: Experimental conditions, full characterizations, ^1^H NMR and ^13^C NMR spectra of all new compounds (PDF); CSP-HPLC traces; UV-Vis, ECD, fluorescence and CPL spectra; computational details. CCDC 1045592 and 1045593. For ESI and crystallographic data in CIF or other electronic format see DOI: 10.1039/c8sc02935k


**DOI:** 10.1039/c8sc02935k

**Published:** 2018-08-01

**Authors:** Alexandre Homberg, Elodie Brun, Francesco Zinna, Simon Pascal, Marcin Górecki, Luc Monnier, Céline Besnard, Gennaro Pescitelli, Lorenzo Di Bari, Jérôme Lacour

**Affiliations:** a Department of Organic Chemistry , University of Geneva , Quai Ernest Ansermet 30 , 1211 Geneva 4 , Switzerland . Email: Jerome.lacour@unige.ch; b Dipartimento di Chimica e Chimica Industriale , Università di Pisa , Via Moruzzi 13 , 56124 Pisa , Italy; c Laboratoire de Cristallographie , University of Geneva , Quai Ernest Ansermet 24 , 1211 Geneva 4 , Switzerland

## Abstract

Straightforward synthesis and resolution of a series of chiral fluorescent macrocycles are presented, together with their electronic circular dichroism (ECD), strong excimer fluorescence (EF, *λ* 300 to 650 nm) and allied highly circularly polarized luminescence (CPL, *g*_lum_ up to 1.7 × 10^–2^). The ECD, EF and CPL responses are strongly affected by the presence of metal ions (Na^+^, Ba^2+^) thanks to deep conformational changes. While ECD signals can be almost completely reversibly inverted upon the complexation/decomplexation of metal ions in a typical binary response, CPL signals are reversibly quenched concomitantly. The designed macrocycles display thus a remarkable combination of both +/– ECD and on/off CPL reversible switching.

## Abstract 2

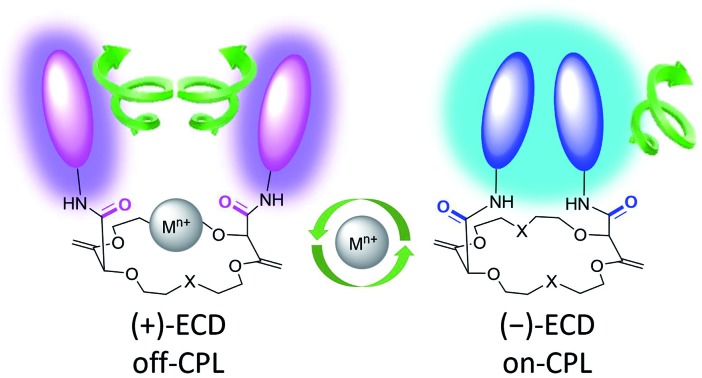
A series of chiral fluorescent macrocycles display a remarkable combination of both +/– ECD and strong on/off CPL reversible switching upon cation binding and displacement.

## Introduction

Tuning the chiroptical properties of molecular or supramolecular systems is of pivotal importance for applications in material science.^1^ Key examples have been reported for the development of switchable memories^2^ and molecular machines,^3^ polarized light emitting devices,^4^ chiral transistors4a,^5^ and chiral spin filters.^6^ Molecules of particular interest are those able to switch their optical properties in response to an external stimulus in a reversible fashion.^7^ Interactions with circularly polarized light, either in absorption or in emission, can then give unambiguous information about molecular states. Reversible electronic circular dichroism (ECD) switches have been reported. They are usually based on metallated helicenes,7a,^8^ gels,^9^ polymers,^10^ lanthanide complexes^11^ or supramolecular assemblies^12^ and can be triggered by light,^3^,^13^ electrochemical processes,^14^ thermal or mechanic stress,^15^ pH variations,^16^ ions coordination^17^ or concentration.^18^

**Fig. 1:**
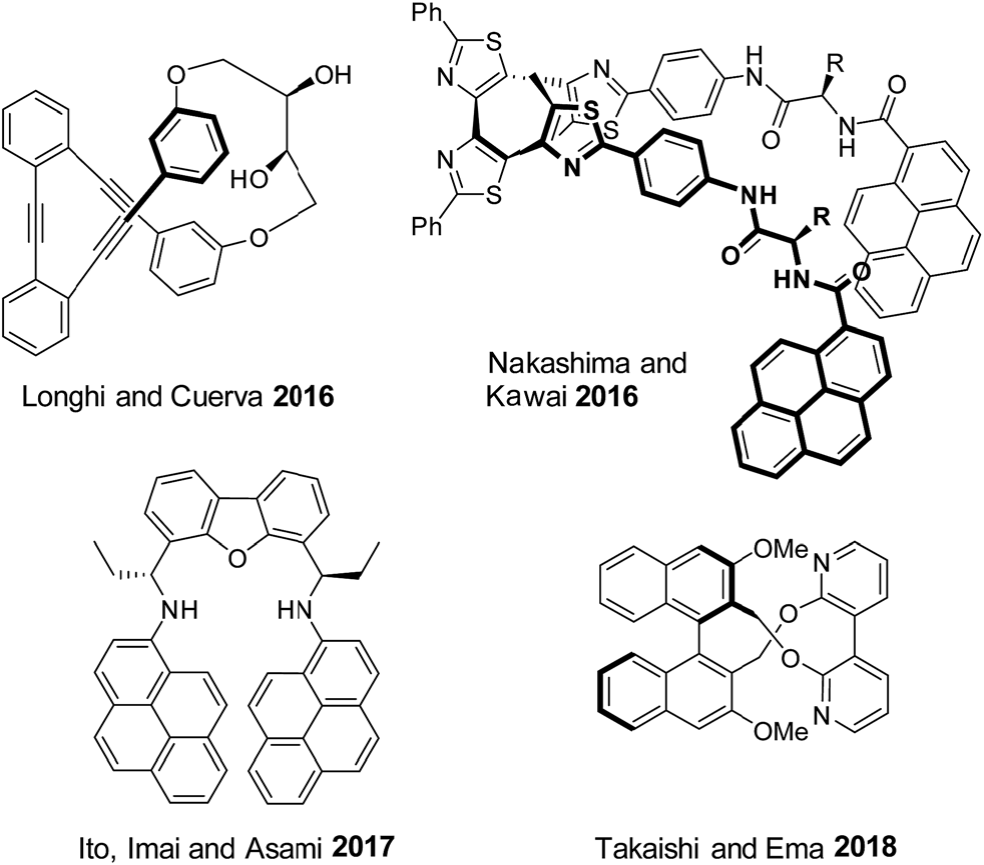
Selected examples of purely organic reversible CPL switches.

In addition to ECD, circular polarization of emitted light can be exploited as a selective read-out. Reversible circularly polarized luminescence (CPL) switches remain however rare,7a,8a,^16^ in particular for purely organic derivatives. For instance (Fig. 1), the groups of Longhi and Cuerva designed a macrocyclic *o*-oligo(phenylene)ethynylene on/off CPL switch by reversible Ag^+^ coordination.17b,^19^ Similarly, Nakashima, Kawai and coworkers reported a reversible on/off CPL switch of a bispyrene-bearing helical tetrathiazole upon photoisomerization.^20^ Ito, Imai, Asami and coworkers synthesized a concentration dependent pyrene-based CPL switch.^18^ Finally, Takaishi and Ema developed a macrocyclic acid/base triggered on/off CPL switch comprising of a binaphthyl linked to a 3,3′-bipyridyl moiety.^21^

**Scheme 1:**
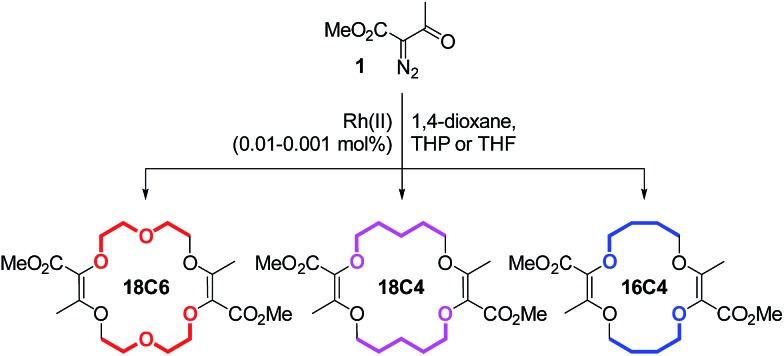
Synthesis of unsaturated macrocycles **18C6**, **18C4** and **16C4**.

**Fig. 2:**
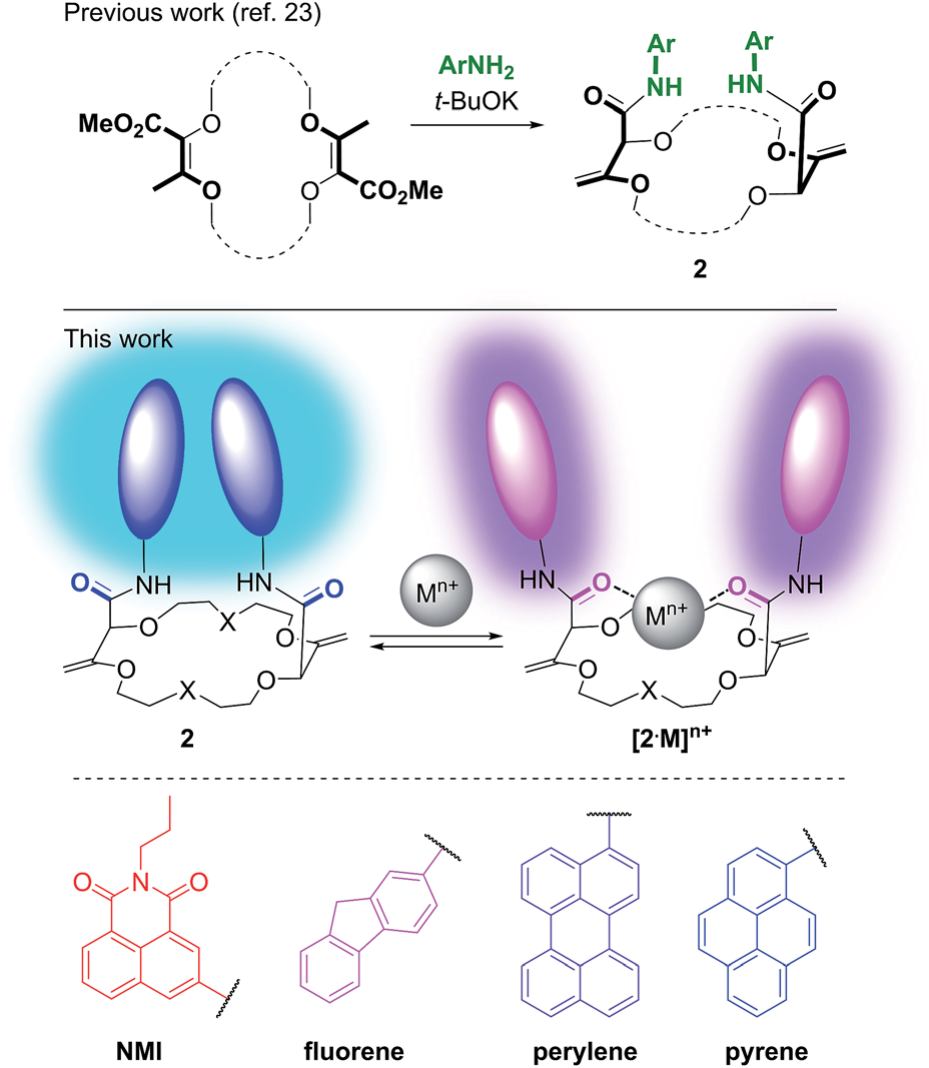
Synthesis of chiral macrocycles (top). Proposed conformational change upon cation binding (middle). Selected fluorophores (bottom).

In this framework, our group recently reported the efficient large-scale synthesis of macrocycles using simple cyclic ethers and diazoketoester **1** as reagents.^22^ Resulting unsaturated derivatives, denominated **18C6**, **18C4** or **16C4** (Scheme 1), react with aromatic amines to form chiral functionalized scaffolds **2** through tandem amidation/olefin transposition processes (Fig. 2, top).^23^ Of importance for the current study, products **2** assume a limited number of restricted conformations with the carbonyl groups facing outwards and the two aromatic units at immediate spatial proximity to each other (Fig. 2).^24^

If pyrenes are introduced as aromatic nuclei, then strong tell-tale excimer fluorescence (EF) is observed.^25^ However, upon binding of monovalent or divalent cations, major conformational changes occur. Presumably, the amide bonds rotate and the resulting inward orientation of the C=O bonds helps complex the cations.^26^ As a consequence, the aromatic rings part from each other, elongating the distance between the chromophores (Fig. 2). The excimer fluorescence is quenched and only the characteristic emission of (monomeric) pyrene is observed.

Herein, we took advantage of this unique ability to induce and cancel excimer fluorescence to develop a family of enantiopure chiroptical switches with a large and tunable wavelength emission range. For this purpose, a variety of fluorophores – namely NMI (NMI: *N*-propyl-1,8-naphthalene monoimide), fluorene, perylene and pyrene (Fig. 2, bottom) – were selected and introduced on different macrocyclic scaffolds (10 examples). After resolution by chiral stationary phase (CSP) HPLC, high CPL response (*g*_lum_ up to 1.7 × 10^–2^) allied with intense excimer fluorescence was demonstrated. In presence of metal ions (Na^+^, Ba^2+^), the ECD, EF and CPL responses are strongly affected. While the ECD signals can be almost completely reversibly inverted upon the complexation/decomplexation of metal ions in a typical binary +/– response, the CPL signal is reversibly quenched establishing a rare combined reversible switching of ECD and extinction of CPL behavior for the designed macrocycles.

## Results and discussion

### Fluorescent macrocycles: synthesis and resolution

**Fig. 3:**
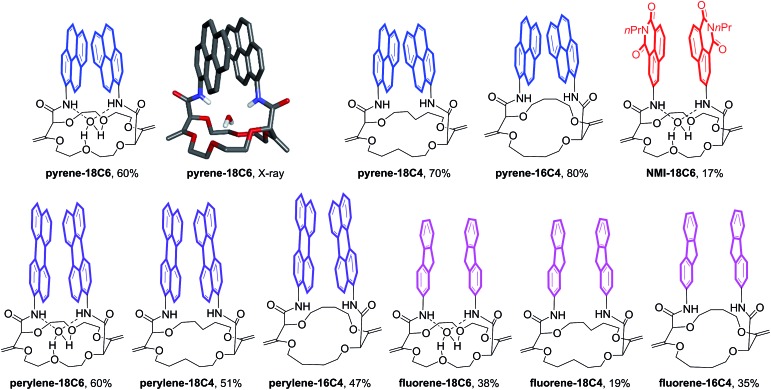
Functionalized macrocycles of study.

Unsaturated macrocycles **18C6**, **18C4** and **16C4** (Scheme 1) were selected as building blocks. The synthesis of **18C6** was achieved on multigram scale by Rh(ii)-catalyzed (0.001 mol%) decomposition of α-diazo-β-keto ester **1** in 1,4-dioxane as solvent and reactant (Scheme S1†).22*a* Application of this procedure to the gram scale preparation of **18C4** and **16C4** was possible with a larger amount of catalyst (0.01 mol%) and THP/THF as cyclic ethers respectively (Scheme S1†).^27^ Treatment of the three derivatives with aromatic amines (3.0 equiv.)^28^ under strongly basic conditions (*t*-BuOK, 4.0 equiv.) afforded the corresponding functionalized macrocycles in moderate to good yields (17–80%, Fig. 2 and 3).^23^,25b In this manner, 1-amino-pyrene, 3-amino-NMI, 2-amino-fluorene^29^ and 1-amino-perylene were introduced effectively. In all cases, the diastereoselectivity of the reaction was excellent (d.r. > 49 : 1) in favor of the chiral (racemic) diastereomers.

With the ten compounds in hand, the CSP-HPLC resolution was performed. A general and practical protocol was developed using eluents in which the macrocyclic derivatives are (highly) soluble to render the semi-preparative enantiomer separation operational. It was found that CHIRALPAK® IG as CSP and a mixture of CH_2_Cl_2_ (+0.1% Et_2_NH) and CH_3_CN (+0.1% Et_2_NH) as mobile phase was particularly efficient.^30^ Good selectivity factors (*α*) and pure samples of both enantiomers were always obtained (see Table S1†).

### UV-Vis absorption and switching of ECD properties

**Fig. 4:**
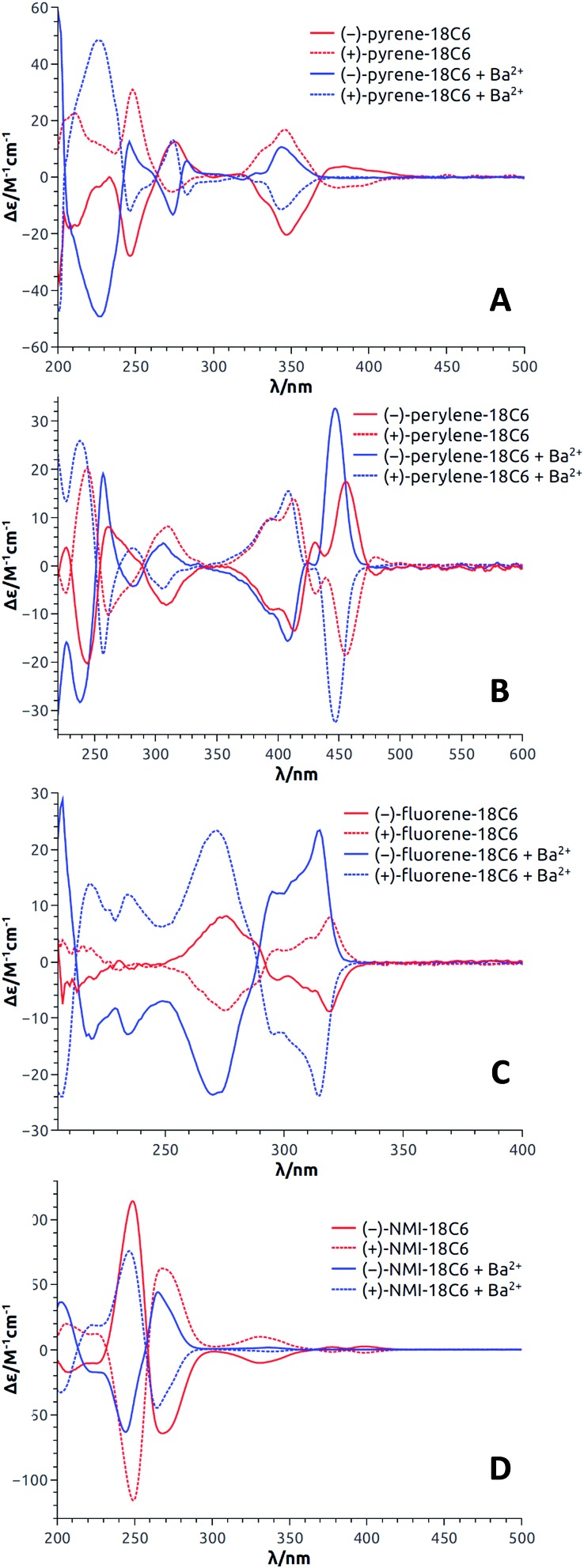
ECD spectra (CH_3_CN) of both enantiomers of **pyrene-18C6** (A), **perylene-18C6** (B), **fluorene-18C6** (C) and **NMI-18C6** (D) without (red) and with (blue) Ba(ClO_4_)_2_.

With enantiopure materials in hand, the UV-Vis absorption and ECD spectra were recorded in CH_3_CN for **18C6**-based macrocycles and in CH_2_Cl_2_ for **18C4**- and **16C4**-based derivatives (Fig. 4 and S5–S17†); the reason for the solvent swap will become clear later in the manuscript. The **perylene** and **fluorene** series (Fig. 4B and C, S16B and C and S17B and C†) displayed strong exciton couplets (bisignate ECD features) in correspondence with their most red-shifted absorption band, which is broad and intense in both cases. The exciton couplets are symmetric (the two component bands have similar integrals) and show clear vibrational sub-structures. Interestingly and somewhat surprisingly, the ECD spectra of **pyrene** derivatives displayed only a monosignate signal for the first absorption band (Fig. 4A, S16A and S17A†). In fact, an exciton coupling was expected for this transition in view of the crystallographic geometry that indicates a spatial proximity and a skewed arrangement of the two aromatic moieties.^31^ For these derivatives, some discrepancy between solution and solid state structures is likely to occur, including the possibility of a dynamic reorientation of the aromatic rings. Finally, **NMI-18C6** displayed intense exciton couplets in the region between 230 and 300 nm (Fig. 4D), where strong absorptions occur, and very weak ECD bands on the right edge of the spectrum, in correspondence with weaker absorption bands.

ECD spectra of **18C6** macrocycles were simulated by means of time-dependent density functional theory (TDDFT) calculations^32^ with the aim of interpreting the observed chiroptical properties and possibly establishing the absolute configuration of the macrocycles with respect to the elution order. Input geometries were generated starting from the available X-ray structure of **pyrene-18C6** with (*S*,*S*) configuration (bound to one water molecule), and optimized with DFT using the M06-2X functional with D3 dispersion correction,^33^ to properly describe intermolecular π-stacking. TDDFT calculations were run with different combinations of functionals, basis sets and environment description (see ESI†). From the start, the attempted accurate simulation of the ECD spectra of the functionalized **18C6**-based macrocycles was a formidable task because of (a) the molecular size, associated with the complexity of the involved chromophores, and (b) the conformational ambiguity. In fact, even assuming a strong preference for π-stacked structures in solution, the two *N*-aryl amide moieties can assume several reciprocal arrangements, by varying the orientation of the macrocycle–C(=O) and *N*–aryl bonds, which are likely to produce very different ECD spectra. This is illustrated in Fig. S36† for **fluorene-18C6**. The most immediate result was that, for any given input structure, a strong exciton-coupled ECD spectrum was calculated in each case. This is easily appreciable in the DFT-optimized geometries for **fluorene-18C6** in which the aromatic rings are partially stacked over each other defining a negative or positive exciton chirality, depending on the reciprocal arrangement of the fluorene rings, associated with strong negative and positive ECD couplet, respectively (Fig. S36†).

If the relative energies from DFT optimizations are entirely trusted, the Boltzmann-weighted calculated ECD spectrum for (*S*,*S*)-**fluorene-18C6** consists into a negative exciton couplet at long wavelength, as found experimentally for the 1^st^ eluted enantiomer (Fig. 4C). When a clear-cut couplet is not apparent from experimental spectra, like for the red-shifted bands of **pyrene-18C6** or **NMI-18C6** (Fig. 4A and D), the reason must be sought in the coexistence of multiple conformations with different arrangements of the aromatic rings which yield ECD spectra canceling each other in the red-shifted portion of the spectrum, as illustrated in Fig. S37.† The calculated ECD profiles suggest that for **pyrene**-, **perylene**- and **NMI-18C6**, the 2^nd^ eluted enantiomers have (*S*,*S*) configuration.

In the presence of monovalent or divalent cations, very different spectroscopic results were observed. Experimentally, it was necessary to use two different sets of conditions depending on the nature of the macrocyclic cores. Within the **18C6** series, studies were conducted in acetonitrile with Ba(ClO_4_)_2_ as metal ion source.25*a* With **18C4**- and **16C4**-based macrocycles, conditions had to be changed to (soluble) NaBAr_F_ in CH_2_Cl_2_ to achieve a modulation of the optical properties.^34^ While little effect was observed in UV-Vis absorption, strong variations of the signals were obtained in ECD in the presence of Na^+^ or Ba^2+^ ions; the chiroptical spectroscopy being particularly sensitive to conformational rearrangements.^35^ All changes were quantified using eqn (1), in which Δ*ε*_(cation)_ and Δ*ε*_(without)_ represent the normalized ECD intensity (in Δ*ε*) of the compound in presence or absence of tested metal ions, respectively. The δΔ*ε* values are summarized in Table 1.

1 δΔ*ε* = |Δ*ε*_(cation)_ – Δ*ε*_(without)_|

**Table 1:** Chiroptical properties (*g*_abs_, δΔ*ε* and *g*_lum_), without and with cations^*a*^ ^,^^*b*^

Compound	Enantiomer	*λ*_abs_ (nm)	*g*_abs_ (10^–4^)		δΔε^*c*^ (M^–1^ cm^–1^)	*λ*_em_ (nm)	*g*_lum_ (10^–2^)	
			Without M^*n*+^	With M^*n*+^			Without M^*n*+^	With M^*n*+^^*d*^
**Pyrene-18C6**	(–)^*e*^	345	–3.9	+1.6	30	490	+0.9	n.d.
	(+)^*f*^		+3.9	–1.6			–0.88	n.d.
**Pyrene-18C4**	(–)^*e*^	348	–10	+5.2	45	481	–1.0	n.d.
	(+)^*f*^		+9.2	–5.4			+0.99	n.d.
**Pyrene-16C4**	(–)^*e*^	342	–0.83	+4.6	11	491	+1.7	n.d.
	(+)^*f*^		+0.6	–4.6			–1.8	n.d.
**Perylene-18C6**	(–)^*e*^	446	+1.2	+5.5	21	543^*g*^	+0.3	+0.05
	(+)^*f*^		–1.3	–5.5			–0.3	–0.05
**Perylene-18C4**	(+)^*e*^	446	+5.2	+23	12	536^*g*^	+0.6	+0.04
	(–)^*f*^		–5.4	–24			–0.6	–0.04
**Perylene-16C4**	(+)^*e*^	446	+1.6	+3.3	8	544^*g*^	–0.4	+0.02
	(–)^*f*^		–1.8	–3.6			+0.4	–0.02
**Fluorene-18C6**	(–)^*e*^	314	–3.6	+5.6	23	337	—	—
	(+)^*f*^		+3.5	–5.6			—	—
**Fluorene-18C4**	(–)^*e*^	315	<0.1	+6.3	6	338	—	—
	(+)^*f*^		<0.1	–6.7			—	—
**Fluorene-16C4**	(+)^*e*^	316	+3.3	+6.3	6	336	—	—
	(–)^*f*^		–3.5	–6.5			—	—
**NMI-18C6**	(–)^*e*^	398	+2.6	+0.2	3	485	+0.82	n.d.
	(+)^*f*^		–2.5	–0.2			–0.86	n.d.

^*a*^Ba(ClO_4_)_2_/CH_3_CN system used for **18C6** derivatives, NaBAr_F_/CH_2_Cl_2_ system used for **18C4** and **16C4** compounds, *g*_abs_ and *g*_lum_ are calculated on reported *λ*_abs_ and *λ*_em_ respectively (unless otherwise stated).

^*b*^n.d. = not detected.

^*c*^As defined by eqn (1).

^*d*^Determined on the monomer emission band.

^*e*^First eluted.

^*f*^Second eluted.

^*g*^ *λ* associated with excimer emission.

The results with **pyrene-18C6**, **perylene-18C6**, **fluorene-18C6** and **NMI-18C6** are first discussed; the spectra for each enantiomeric series being presented in Fig. 4. Importantly and interestingly, for each compound, transition(s) can always be found for which the sign of the Cotton effect switches from positive to negative or *vice versa*. In the case of **pyrene-18C6**, the spectra undergo remarkable ECD sign reversal for almost all the observed transitions in the 200–500 nm range (Fig. 4A). With **perylene-18C6**, the ECD switch is less dramatic but still significant at 446 nm (Fig. 4B). It might be due to the bulkier nature of the perylene moiety, which does not allow for the necessary conformational freedom to attain a complete rearrangement upon addition of the barium salt. However, the **fluorene-18C6** derivative presents not only the sign reversal in the exciton couplet region but also a strong increase of the intensity (Fig. 4C). For **NMI-18C6**, sign inversion is observed at a higher energy (around 260 nm) for the exciton coupling-like feature (Fig. 4D). For completeness and as control experiments, the ECD behavior was also monitored in the presence of NaBAr_F_ in CH_2_Cl_2_; variations upon addition of Na^+^ were very similar to that with Ba^2+^ in this **18C6** series (Fig. S15†). With compounds **pyrene-18C4**, **perylene-18C4** and **fluorene-18C4**, similar experiments were conducted but only with NaBAr_F_ (Fig. S16†). This was also the case for **pyrene-16C4**, **perylene-16C4** and **fluorene-16C4** (Fig. S17†). Globally, the trends detailed above are reproduced for the macrocycles with all-carbon links between the polar units. A small difference is displayed by **fluorene-16C4** which shows a couplet of different sign with respect to compounds **fluorene-18C6** and **fluorene-18C4**. The intensity of this couplet increases but it does not change sign upon Na^+^ addition.

### Switching of fluorescence and CPL properties

**Fig. 5:**
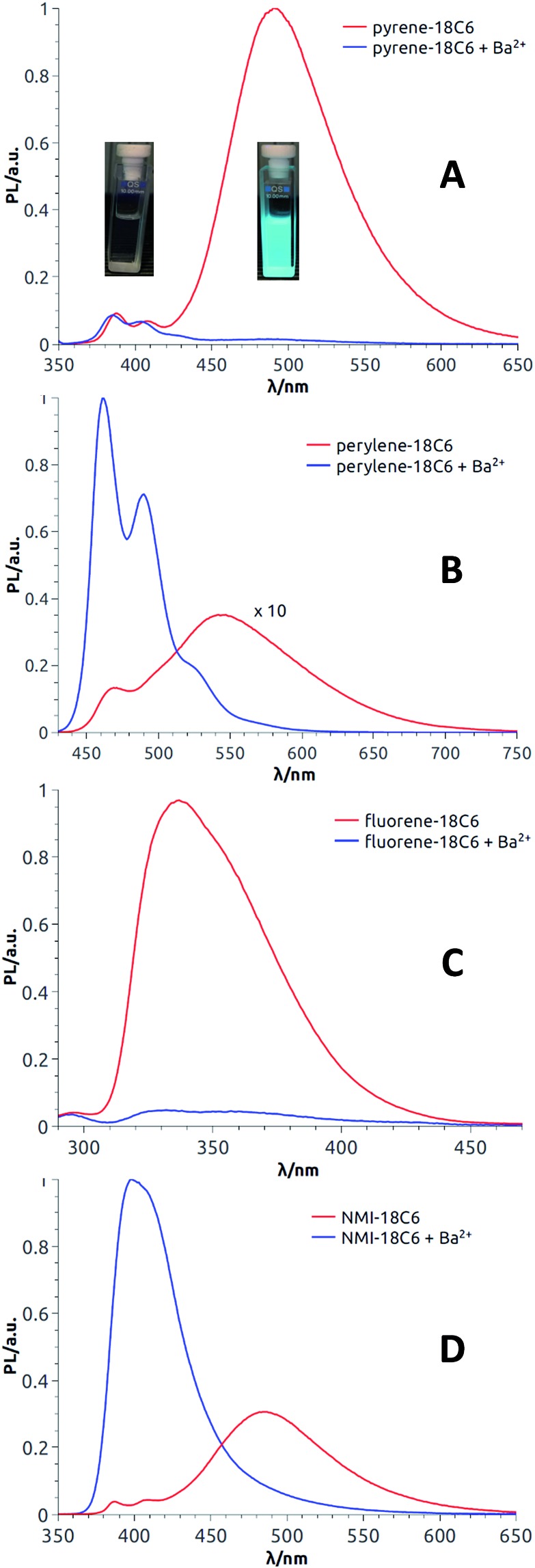
Fluorescence spectra (CH_3_CN) of **pyrene-18C6** (A), **perylene-18C6** (B), **fluorene-18C6** (C) and **NMI-18C6** (D) without (red) and with (blue) Ba(ClO_4_)_2_. In panel A: picture of **pyrene-18C6** without (left) and with (right) Ba^2+^ under UV irradiation (366 nm).

**Fig. 6:**
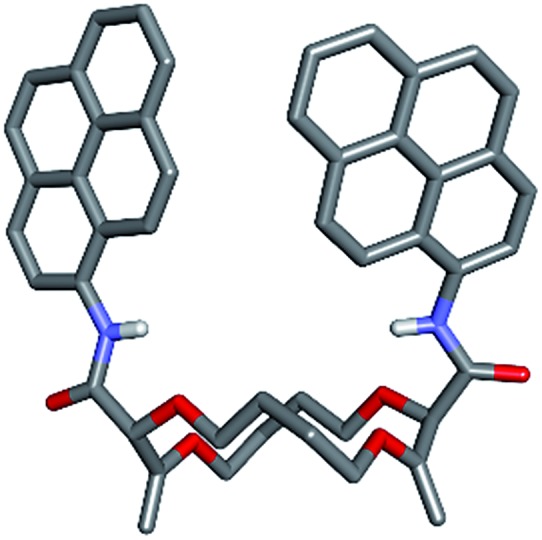
Stick view of the crystal structure of **pyrene-16C4**.

Satisfactorily, all functionalized macrocycles displayed excimer fluorescence (Fig. 5 and S5–S14†). As expected, the emissions are red shifted compared to “monomer” emissions; a series of simple neopentylcarboxamides being used as references (see the ESI† for the synthesis and characterizations). In the **pyrene** series, the excimer band is particularly intense at 490 nm (*λ*_max_) and, for the **18C4** moiety specifically, the interaction is so strong that the emission band of the monomer is no longer observable. With compounds carrying **perylene** moieties, a weaker and broad EF is observed around 540 nm. **Fluorene** derivatives present very strong EF (337 nm) in comparison to the almost non-detectable monomer band. Finally, **NMI-18C6** presents similar characteristics to **perylene-18C6** although with a more intense excimer band (485 nm) in respect to the monomer. Globally, with this family of compounds, the excimer fluorescence spans a spectral window from UV to green-yellow (*ca.* 300 nm to 650 nm). Also, while **18C6** and **18C4** derivatives display strong EF contribution with respect to the monomer fluorescence, the effect is less pronounced with **16C4** analogues. Actually, for the smaller ring size compounds, solid-state analysis indicates a non-parallel arrangement of the aromatic moieties (Fig. 6) and hence the possible reduction in EF efficiency.

Upon addition of either Na^+^ or Ba^2+^ cations, full quenching of the excimer fluorescence is observed, and this with all fluorophores, independently of their macrocycle size and nature. Only the sharper monomer emissions remain. This observation is in agreement with the proposed conformational rearrangement upon cation complexation (*vide supra*, Fig. 2). Emission maxima (*λ*_max_) and fluorescence quantum yields (*φ*) of all compounds are reported in Table S3.†

**Fig. 7:**
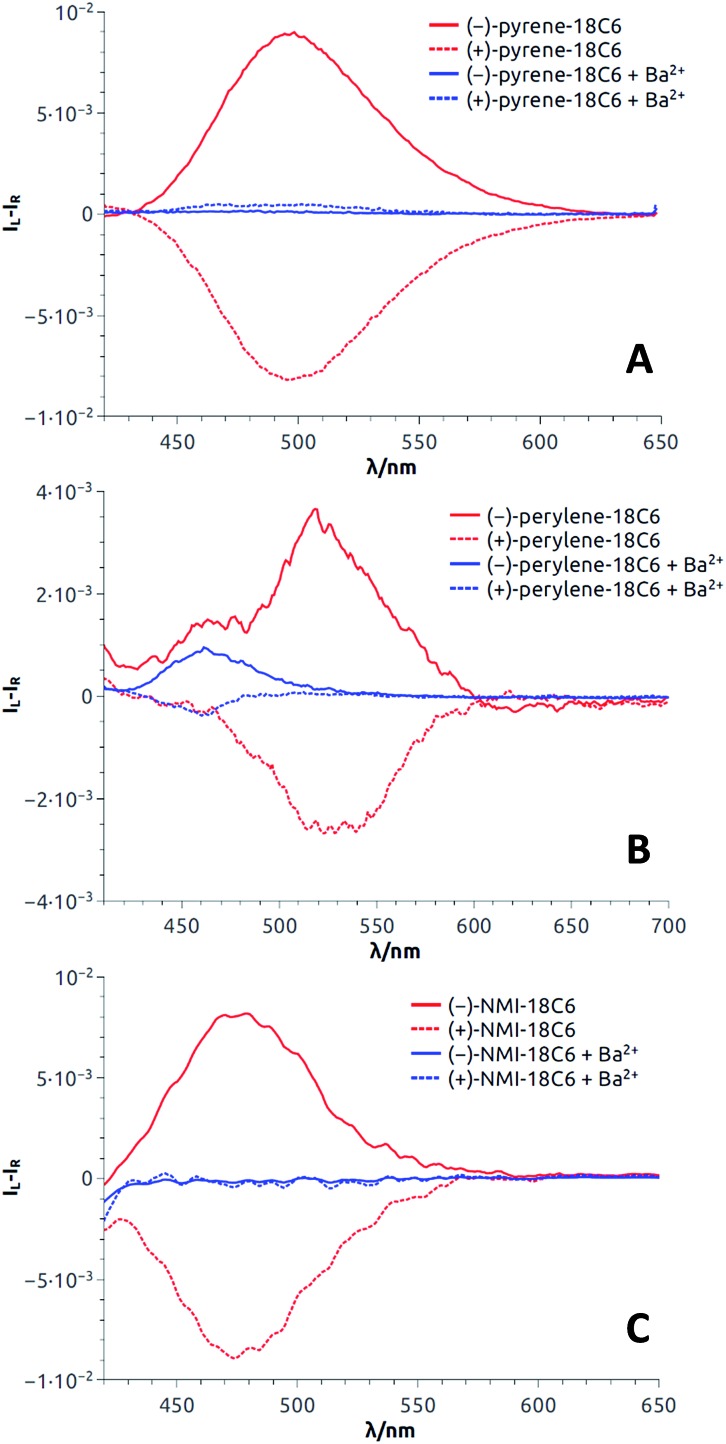
CPL spectra (CH_3_CN) of both enantiomers of **pyrene-18C6** (A), **perylene-18C6** (B) and **NMI-18C6** (C) without (red) and with (blue) Ba(ClO_4_)_2_.

With these results in hand demonstrating (very) effective EF, circularly polarized luminescence studies were started expecting, in accordance with previously reported examples,^18^,^20^,^36^,^37^ efficient CPL emission allied with the excimer band. In fact and as expected, **pyrene**, **perylene** and **NMI** derivatives presented strong excimer-associated CPL (Fig. 7, S18 and S19†); a weak contribution from the monomer circularly polarized emission being observed only in the **perylene** series (Fig. 7B, S18B and S19B†). In the **fluorene** series, due to the technical limitations of the CPL apparatus in the UV region, CPL spectra could not be measured. To quantify the circular polarization degree of the emission, the luminescence dissymmetry factor *g*_lum_ was used as defined by eqn (2)
2

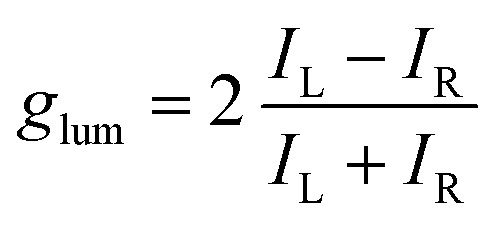

where *I*_L_ and *I*_R_ correspond to left and right circularly polarized component of the emission respectively. The *g*_lum_ factors for all tested compounds are reported in Table 1.

For most compounds, the luminescence dissymmetry factors calculated on the CPL/fluorescence maxima are around 10^–2^. These *g*_lum_ values are in the upper range recorded for single (*i.e.* non-aggregated) organic molecules,^38^ and in line with that of excimer bands formed by pyrenes,^18^,^20^,^39^ naphthalimides^40^ and naphthalene diimides^41^ or perylene bisimides.^42^ In the **perylene** series, *g*_lum_ values not higher than 10^–3^ were obtained. The decrease of the observed dissymmetry factor is possibly due to the contribution of the weakly polarized perylene monomer emission (Table 1).^43^

Since, in these systems, CPL stems from the excimer, a photophysical state formed only in the excited state, the *g*_lum_ factor does not need to be related to the ECD dissymmetry factor (*g*_abs_ = Δ*ε*/*ε*) measured on the first Cotton effect, as it would be expected if the same electronic states were involved in absorption and emission.^44^ Indeed, in the present case, *g*_abs_ values are in the 10^–3^ to 10^–4^ range while *g*_lum_ factors, calculated on the CPL/fluorescence maxima, are higher by at least one order of magnitude (10^–2^).

Then, as it could be expected from the EF experiments, full quenching of CPL signals was achieved for **pyrene** and **NMI** derivatives upon addition of either Na^+^ or Ba^2+^ cations (Fig. 7A and C, S18A and S19A†). In these cases, the monomer bands are too weak to be detected. However, with **perylene** compounds (Fig. 7B, S18B and S19B†), it was possible to measure a weak CPL signal allied to the strong monomer emission (*g*_lum_ up to 5 × 10^–4^), in the expected *g*_lum_ range for (weakly) chirally-perturbed fluorophores.

### ECD/CPL reversibility

**Scheme 2:**
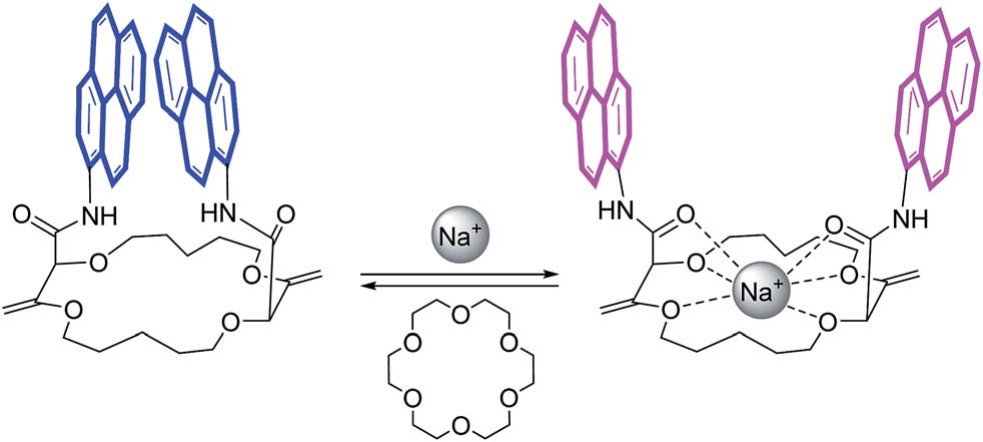
Model for reversible switching.

All these experiments bode well for the development of effective +/– ECD and on/off CPL switches upon the establishment of reversible metal complexation conditions with the compounds at hand. In view of the strong binding of barium cations to **18C6** derivatives in acetonitrile, care was taken to study the reversibility of the complexation of monovalent cations instead.25*a* For practical reasons, we selected the conditions already established, *i.e*. NaBAr_F_ in CH_2_Cl_2_ and decided to use regular 18-Crown-6 as a cation scavenger (Scheme 2).^45^

**Fig. 8:**
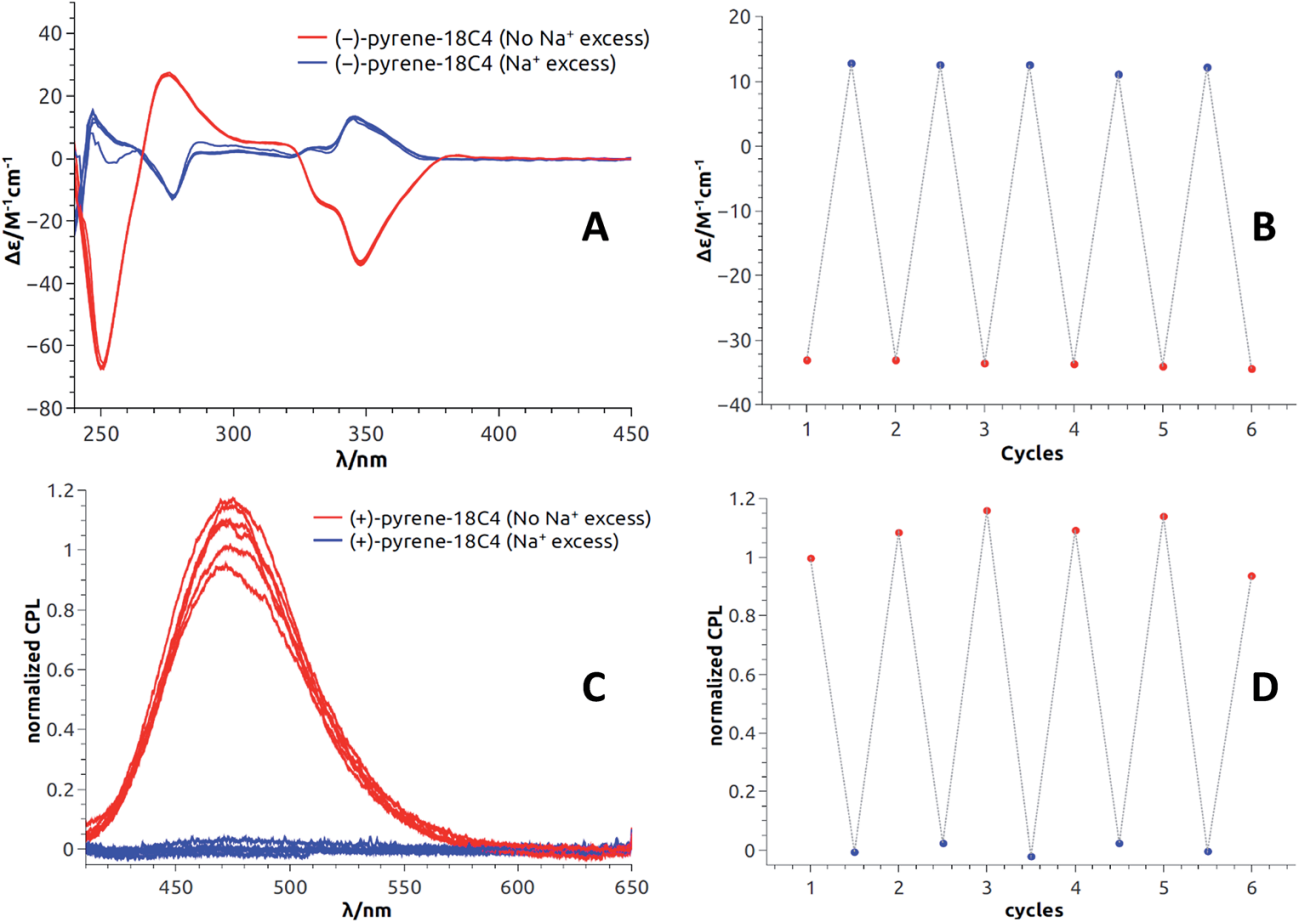
**Pyrene-18C4** (CH_2_Cl_2_): Reversible ECD ((A) first eluted (–)-enantiomer) and intensities at 348 nm (B). Reversible CPL ((C) second eluted (+)-enantiomer) and normalized intensities at 480 nm (D).

Equimolar amounts of NaBAr_F_ and 18-Crown-6 solutions were thus successively added to dichloromethane solutions of the **pyrene**, **perylene**, **fluorene** and **NMI** fluorophores. After each addition, ECD, then fluorescence and finally CPL spectra were recorded. Data for the **pyrene-18C4** derivative, selected as a representative example, are displayed in Fig. 8. Remaining data are reported in ESI (Fig. S20–S35).† As desired, upon addition of commercial 18-Crown-6 to the Na^+^ adduct, it was possible to completely switch back the system and recover the ECD signals of the uncomplexed material (Fig. 8A). The switch experiment could be repeated over several cycles without significant signal loss or modification (*e.g.*, Fig. 8B at 348 nm). Using the same **18C4** model, almost complete reversible switching – from excimer to monomer – was monitored by fluorescence (Fig. S30†). In CPL, these conditions led also to a very good recovery of the signal, over several cycles (Fig. 8C and D). **Pyrene-18C4** may therefore be considered as a completely reversible +/– ECD and on/off CPL switch.

Appreciatively, using the same reagent combination, all pyrene and fluorene macrocycles behave as fully reversible ECD switches over several cycles (Fig. S20–S21 and S25–S27†). In CPL, **pyrene-18C6** demonstrated reversibility in spite of an attenuation of the excimer luminescence intensity over the cycles (Fig. S34†). This trend is even more acute with analogous **pyrene-16C4** that showed reversibility over only two cycles – presumably due to the strong photobleaching occurring under the CPL measurement conditions for this compound (Fig. S35†). Finally, for **perylene** and **NMI-18C6** derivatives, signal recovery to the expected intensity and shape could not be achieved over successive additions of NaBAr_F_ and 18-Crown-6 (Fig. S22–S24 and S28†). The reversibility of the CPL signal was hence not studied for these cases.

## Conclusions

In summary, we presented a series of readily prepared functionalized macrocycles showing efficient excimer luminescence – in different spectral regions – due to the spatial proximity of fluorophores held in place by the constrained macrocyclic structures. Using enantiopure materials, obtained through CSP-HPLC resolution, a sign inversion of ECD signal(s) was demonstrated in all cases upon addition of cations like Na^+^ or Ba^2+^. In addition, these molecules display a highly circularly polarized luminescence associated with the excimer fluorescence with *g*_lum_ values up to 10^–2^, which can be quenched upon cation binding. For several systems, a complete recovery of ECD/CPL signals over several cycles was observed after reversible complexation of the cation, Na^+^ specifically together with commercial 18-Crown-6 as sequester, giving rise to rare examples of allied +/– ECD and on/off CPL switches.

## Conflicts of interest

There are no conflicts to declare.

## Abbreviation

CPL: Circularly polarized luminescence
ECD: Electronic circular dichroism
EF: Excimer fluorescence
NMI: *N*-Propyl-1,8-naphthalene monoimide

## Supplementary Material

Supplementary information

Crystal structure data
