# Supplementary material for: Combined reversible switching of ECD and quenching of CPL with chiral fluorescent macrocycles
Source: Chem Sci. 2018 Aug 1;9(35):7043–52. doi: 10.1039/c8sc02935k (PMC6137439; doi:10.1039/c8sc02935k)
Supplement: Supplementary file 1 [file SC-009-C8SC02935K-s001.pdf]

Electronic Supplementary Material (ESI) for Chemical Science.
This journal is © The Royal Society of Chemistry 2018

Combined reversible switching of ECD and quenching of CPL with chiral fluorescent macrocycles

Supporting Information

Alexandre Homberg,[1] Elodie Brun,[1] Francesco Zinna,[1] Simon Pascal,[1] Marcin Górecki,[2] Luc Monnier,[1] Céline Besnard,[3] Gennaro Pescitelli,[2] Lorenzo Di Bari,[2] Jérôme Lacour[1]*

[1] Department of Organic Chemistry, University of Geneva, Quai Ernest Ansermet 30, 1211 Geneva 4, Switzerland.

[2] Dipartimento di Chimica e Chimica Industriale, Università di Pisa, Via Moruzzi 13, 56124 Pisa, Italy.

[3] Laboratoire de Cristallographie, University of Geneva, Quai Ernest Ansermet 24, 1211 Geneva 4, Switzerland.

Table of Content

1. General Information and Materials S4
1.1. Synthesis and characterization S4
1.2. CSP-HPLC S4
1.3. Optical properties S5
2. Synthesis and characterization of organic compounds S6
2.1. Synthesis of anilines S6
2.1.1. Synthesis of 3-nitro-perylene S6
2.1.2. Synthesis of 3-amino-perylene S6
2.2. Synthesis of monomeric amides S7
2.2.1. General procedure for the synthesis of monomeric amides S7
2.2.2. Pyrene monomer S8
2.2.3. Perylene monomer S9
2.2.4. Fluorene monomer S10
2.2.5. NMI monomer S11
2.3. Synthesis of polyether macrocycles **16C4** and **18C4** S12

2.3.1. Synthesis of polyether macrocycle **16C4** S12
2.3.2. Synthesis of polyether macrocycle **18C4** S13
2.4. Synthesis of functionalized macrocycles S14
2.4.1. General procedure for the synthesis of macrocycles S14
2.4.2. Perylene-18C6 ..... S15
2.4.3. Perylene-18C4 ..... S16
2.4.4. Perylene-16C4 ..... S17
2.4.5. Fluorene-18C6 ..... S18
2.4.6. Fluorene-18C4 ..... S19
2.4.7. Fluorene-16C4 ..... S20
2.4.8. NMI-18C6 ..... S21
2.5. Monomeric amides NMR spectra S22
2.5.1. Pyrene monomer S22
2.5.2. Perylene monomer S23
2.5.3. Fluorene monomer S24
2.5.4. NMI monomer S25
2.6. Polyether macrocycles **16C4** and **18C4** NMR spectra S26
2.6.1. Polyether macrocycles **16C4** S26
2.6.2. Polyether macrocycles **18C4** S27
2.7. Functionalized polyether macrocycles NMR spectra S28
2.7.1. Perylene-18C6 ..... S28
2.7.2. Perylene-18C4 ..... S29
2.7.3. Perylene-16C4 ..... S30
2.7.4. Fluorene-18C6 ..... S31
2.7.5. Fluorene-18C4 ..... S32
2.7.6. Fluorene-16C4 ..... S33
2.7.7. NMI-18C6 ..... S34
3. CSP-HPLC separation S35
3.1. General remarks S35
3.2. Summary of separation data S36
3.3. HPLC traces S36
3.3.1. Pyrene-18C6 ..... S37
3.3.2. Pyrene-18C4 ..... S38
3.3.3. Pyrene-16C4 ..... S39

3.3.4. Perylene 18C6 .... S40
3.3.5. Perylene-18C4 .... S41
3.3.6. Perylene-16C4 .... S42
3.3.7. Fluorene-18C6 .... S43
3.3.8. Fluorene-18C4 .... S44
3.3.9. Fluorene-16C4 .... S45
3.3.10. NMI-18C6 .... S46
4. Spectroscopic data S47
4.1. UV-Vis absorption and fluorescence spectra S47
4.1.1. Procedure S47
4.1.2. Summary of the optical properties S47
4.1.3. Spectra of the monomeric amides without and with $Ba(ClO_4)_2$ S48
4.1.4. Spectra of the macrocycles without and with cations S50
4.2. ECD spectra S55
4.2.1. General procedure S55
4.2.2. ECD spectra without and with $NaBAr_F$ S56
4.3. CPL spectra S59
4.3.1. General procedure S59
4.3.2. CPL spectra without and with $NaBAr_F$ S59
4.4. Reversibility S60
4.4.1. Procedure S60
4.4.2. ECD reversibility S60
4.4.3. Fluorescence reversibility S64
4.4.4. CPL reversibility S66
5. Computational Section S68
5.1. General remarks S68
5.2. Calculation results S69
6. Crystallographic Data S72
7. References S74

1. General Information and Materials

1.1. Synthesis and characterization

All reactions involving air sensitive compounds were carried out under N_2 or argon by means of an inert gas/vacuum double manifold line and standard Schlenk techniques using dry solvents (CH_2Cl_2, tetrahydrofuran, tetrahydropyran, 1,4-dioxane and Et_3N). Reactions involving oxygen sensitive reagents were performed using degassed solvents. Unless otherwise stated, all reagents were purchased from commercial sources and used without further purification (*e.g.* 2-amino-fluorene, 1-amino-pyrene, 3-nitro-1,8-naphthalic anhydride, perylene, *tert*-butyl acetyl chloride). *t*-BuOK was sublimed prior to use.

Analytical thin-layer chromatographies (TLC) were performed with Silicagel 60 F_{254} aluminium sheets from Merck. Flash column chromatographies were performed with Silica SiliaFlash P60, 40-63 µm (230-400 mesh) and Aluminium oxide (neutral, Brockmann I, 50-200 µm, 60 Å).
NMR spectra were recorded on a Bruker AVANCE III HD-NanoBay 400 MHz spectrometer, equipped with a 5 mm CryoProbe Prodigy, or on a Bruker II 500 MHz spectrometer, equipped with a 5 mm Cryogenic DCH ($^1H/^{13}C$) probe at 298 K. ^1H NMR chemical shifts are given in ppm relative to Me_4Si using solvent resonances as internal standards ($CDCl_3$ δ = 7.26 ppm, DMSO-d_6 δ = 2.50 ppm, CD_2Cl_2 δ = 5.32 ppm). Data were reported as follows: chemical shift (δ) in ppm, multiplicity (s = singulet, d = doublet, t = triplet, dd = doublet of doublet, q = quartet and m = multiplet), coupling constant (Hz) and integration. ^{13}C-NMR chemicals shifts were given in ppm relative to Me_4Si with solvent resonances used as internal standards ($CDCl_3$ δ = 77.16 ppm, DMSO-d_6 δ = 39.52 ppm, CD_2Cl_2 δ = 53.84 ppm). IR spectra were recorded with a Perkin-Elmer 100 FT-IR spectrometer using a diamond ATR Golden Gate sampling and are reported in wave numbers (cm^{-1}). Melting points (m.p.) were measured in open capillary tubes with a Büchi B-550 melting point apparatus and were uncorrected. Low resolution electrospray mass spectra (LR-ESI-MS) were obtained on an API 150EX (AB/MDS Sciex) spectrometer. High resolution mass spectra (HR-MS) were recorded on a QSTAR Pulsar (AB/MDS Sciex) spectrometer by the Department of Mass Spectroscopy at the University of Geneva. Optical rotations were measured in a thermostated (20 °C) 10 cm long microcell at 589 nm (Na lamp).

1.2. CSP-HPLC

Enantiomers of compounds **pyrene-18C6**, **pyrene-18C4**, **pyrene-16C4**, **perylene-18C6**, **perylene-18C4**, **perylene-16C4**, **fluorene-18C6**, **fluorene-18C4**, **fluorene-16C4** and **NMI-18C6** were resolved by chiral stationary phase HPLC on an Agilent 1260 Infinity II apparatus (quaternary pump, auto sampler, column thermostat and diode array detector) using a semi-preparative CHIRALPAK® IG column (250 x 10 mm, 5 mic). Mixtures of HPLC grade CH_2Cl_2 and acetonitrile (with 0.1% diethylamine as additive) were used as mobile phase. Samples were injected as solutions in pure CH_2Cl_2.

1.3. Optical properties

Optical properties were recorded in analytical grade solvents (CH_2Cl_2, acetonitrile). UV-Vis absorption spectra were recorded on a JASCO V-650 spectrophotometer at 20 °C. Electronic circular dichroism (ECD) spectra were recorded on a Jasco J-815 spectropolarimeter at 20 °C in a 1 cm-cuvette.

Fluorescence spectra were measured using a Varian Cary 50 Eclipse spectrophotometer. All fluorescence spectra were corrected for the wavelength-dependent sensitivity of the detection. Fluorescence quantum yields ϕ were measured in diluted solutions (at least 5 different concentrations for each sample) with an optical density lower than 0.1 using the following equation:

$$\frac{\Phi_x}{\Phi_\mathrm{r}} = \left(\frac{A_r(\lambda)}{A(_x\lambda)}\right)\left(\frac{n_x^2}{n_r^2}\right)\left(\frac{D_x}{D_r}\right)$$

where A is the absorbance at the excitation wavelength (λ), n the refractive index and D the integrated intensity. "r" and "x" stand for reference and sample respectively. The fluorescence quantum yields were measured in acetonitrile relative to phenanthrene (ϕ = 12.5% in EtOH), anthracene (ϕ = 27% in EtOH) or coumarine 153 (ϕ = 38% in EtOH). Excitation of reference and sample compounds was performed at the same wavelength.

The circularly polarized luminescence (CPL) spectra were recorded with the home-made spectrofluoropolarimeter previously described,[1] the samples were excited with an UV (365 nm) LED, using a 90° geometry between excitation and detection.

$Ba(ClO_4)_2$ and $NaBAr_F$ salts used for titration experiments were purchased from commercial sources and used without purification.

2. Synthesis and characterization of organic compounds

2.1. Synthesis of anilines

3-Amino-perylene was synthesized in two steps from commercially available perylene using procedures adapted from the literature.[2]
3-Amino-NMI (NMI: *N*-propyl-1,8-naphthalene monoimide) was prepared according to a previously described procedure starting from commercially available 3-nitro-1,8-naphthalic anhydride.[3]

2.1.1. Synthesis of 3-nitro-perylene

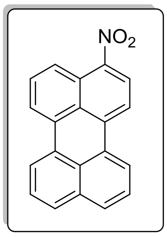

To a solution of perylene (500 mg, 1.98 mmol) in ethyl acetate (30 mL) were added Ac_2O (0.51 mL, 5.4 mmol, 2.7 equiv) and $Cu(NO_3)_2·2.5H_2O$ (690 mg, 2.97 mmol, 1.5 equiv). The mixture was stirred at 25 °C for 15 hours. The conversion was followed by TLC analysis. After completion of the reaction, H_2O (30 mL) and CH_2Cl_2 (30 mL) were added, the layers were separated and the aqueous layer was extracted with CH_2Cl_2 (3 x 50 mL). The combined organic layers were washed with H_2O (3 x 30 mL), dried over anhydrous Na_2SO_4, filtered and concentrated under vacuum. The residue was purified by column chromatography (SiO_2, pentane/CH_2Cl_2 gradient: 8:2, 7:3, 6:4) to yield 500 mg (1.68 mmol, 297.31 g/mol, 85%) of 3-nitro-perylene as a red solid.

Spectral data match those reported in the literature.[2a]

2.1.2. Synthesis of 3-amino-perylene

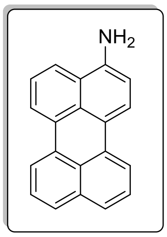

To a suspension of 3-nitro-perylene (480 mg, 1.61 mmol) in EtOH (6 mL) were added Pd/C (10% in weight, 171 mg, 0.161 mmol, 0.1 equiv) and hydrazine (9.7 mL, 1M in THF, 9.7 mmol, 6 equiv). The mixture was heated at 80 °C for 15 h, then cooled to room temperature, filtered through a pad of Celite® and concentrated under vacuum to yield 430 mg (1.61 mmol, 267.33 g/mol, quant.) of 3-amino-perylene as a bright orange solid.

Spectral data match those reported in the literature.[2b]

2.2. Synthesis of monomeric amides

Neopentylcarboxamides, bearing only one fluorophore, were synthesized and used as monomeric references for the analyses and for comparison with the optical properties of macrocyclic compounds.

Pyrene monomer was used as a monomeric reference for **pyrene-18C6**, **pyrene-18C4** and **pyrene-16C4** compounds.

Perylene monomer was used as a monomeric reference for **perylene-18C6**, **perylene-18C4** and **perylene-16C4** compounds.

Fluorene monomer was used as a monomeric reference for **fluorene-18C6**, **fluorene-18C4** and **fluorene-16C4** compounds.

NMI monomer was used as a monomeric reference for **NMI-18C6** derivative.

2.2.1. General procedure for the synthesis of monomeric amides

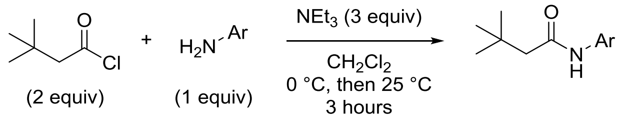

(S1)

In a 10 mL flask under nitrogen atmosphere, dry CH_2Cl_2 (0.1 M) and dry NEt_3 (3 equivalents) were added to 1 equivalent of the aromatic amine. The mixture was cooled down to 0 °C (ice bath) and *tert*-butyl acetyl chloride (2 equivalents) was added in one portion. The cooling bath was removed and the reaction was allowed to reach 25 °C on its own and stirred for an additional 3 hours. The conversion was followed by TLC analysis. After completion, the reaction was quenched by the addition of water and the mixture was extracted three times with CH_2Cl_2. The combined organic layers were dried over anhydrous Na_2SO_4 and concentrated under vacuum. The pure monomeric amide was obtained after purification by column chromatography.

2.2.2. Pyrene monomer

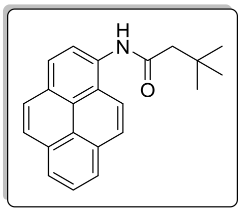

According to the general procedure, 43 mg (0.2 mmol) of 1-amino-pyrene, 56 µL (54 mg, 0.4 mmol) of *tert*-butyl acetyl chloride, 84 µL (61 mg, 0.6 mmol) of dry NEt_3 and 2 mL of dry CH_2Cl_2 yielded 58 mg (0.18 mmol, 315.42 g/mol, 92%) of **pyrene monomer** as an off-white solid.

Purification conditions: Column eluent (SiO_2): CH_2Cl_2/methanol gradient (99.5:0.5, 99:1, 98:2).

R_f = 0.8 (SiO_2, CH_2Cl_2/methanol 98:2)
m.p.: 207 °C - 209 °C
^{1}H-NMR (500 MHz, $CDCl_3$): δ/ppm = 1.25 (s, 9H, $-CH_3$), 2.48 (s, 2H, $-CH_2-$), 7.71 (s, 1H, NH), 7.99 - 8.19 (m, 8H, aromatics), 8.41 (d, 1H, *J* = 8.2 Hz, aromatic).
^{13}C-NMR (126 MHz, $CDCl_3$): δ/ppm = 30.2 (3 $-CH_3$), 31.6 (1 C), 51.8 (1 $-CH_2-$), 120.3 (1 CH aromatic), 122.6 (1 CH aromatic), 123.6 (1 C aromatic), 124.9 (1 C aromatic), 125.2 (1 CH aromatic), 125.3 (1 CH aromatic), 125.3 (1 C aromatic), 125.3 (1 CH aromatic), 125.6 (1 CH aromatic), 127.0 (1 CH aromatic), 127.5 (1 CH aromatic), 128.1 (1 CH aromatic), 129.2 (1 C aromatic), 130.5 (1 C aromatic), 130.9 (1 C aromatic), 131.5 (1 C aromatic), 170.8 (1 C=O).
IR (neat): $\tilde{\nu}$/cm^{-1} 3283, 3034, 2950, 2866, 1650, 1598, 1555, 1521, 1485, 1393, 1364, 1340, 1307, 1271, 1236, 1108, 1040, 961, 913, 841, 822, 794, 755, 709.
HR MS (ESI) $[M+H]^+$ *m/z* calculated for $C_{22}H_{22}NO$ 316.1696, observed 316.1697 (0.2 ppm).

2.2.3. Perylene monomer

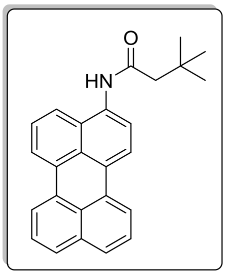

According to the general procedure, 27 mg (0.1 mmol) of 3-aminoperylene, 28 µL (27 mg, 0.2 mmol) of *tert*-butyl acetyl chloride, 42 µL (30 mg, 0.3 mmol) of dry NEt_3 and 2 mL of dry CH_2Cl_2 yielded 31 mg (0.085 mmol, 365.48 g/mol, 85%) of **perylene monomer** as a yellow-orange solid.
Purification conditions: Column eluent (SiO_2): CH_2Cl_2/methanol (98:2).

R_f = 0.4 (SiO_2, CH_2Cl_2/methanol 98:2)
m.p.: 240 °C - 243 °C
^{1}H-NMR (400 MHz, DMSO-d_6): δ/ppm = 1.11 (s, 9H, -CH_3), 2.39 (s, 2H, -CH_2-), 7.53 - 7.57 (m, 2H, aromatics), 7.61 (t, 1H, *J* = 8.0 Hz, aromatic), 7.76 - 7.81 (m, 3H, aromatics), 7.95 (d, 1H, *J* = 8.8 Hz, aromatic), 8.32 (d, 1H, *J* = 7.4 Hz, aromatic), 8.37 (t, 2H, *J* = 8.3 Hz, aromatics), 8.41 (d, 1H, *J* = 7.0 Hz, aromatic), 9.81 (s, 1H, NH).
^{13}C-NMR (126 MHz, DMSO-d_6): δ/ppm = 29.8 (3 -CH_3), 31.0 (1 -CH_2-), 49.1 (1 C), 120,5 (1 C aromatic), 120.7 (1 C aromatic), 120.9 (1 C aromatic), 121.0 (1 C aromatic), 122.5 (1 C aromatic), 122.7 (1 C aromatic),126.6 (1 C aromatic), 126.9 (1 C aromatic), 127.0 (1 C aromatic), 127.4 (1 C aromatic), 127.5 (1 C aromatic), 127.7 (1 C aromatic), 128.0 (1 C aromatic), 128.5 (1 C aromatic), 128.6 (1 C aromatic), 129.7 (1 C aromatic), 130.5 (1 C aromatic), 130.8 (1 C aromatic), 133.7 (1 C aromatic), 134.3 (1 C aromatic), 170.6 (1 C=O).
IR (neat): ṽ/cm^{-1} 3279, 2957, 2876, 1651, 1535, 1526, 1391, 1366, 1340, 1270, 1260, 1195, 1150, 1051, 989, 834, 814, 767.
HR MS (ESI) $[M+H]^+$ *m/z* calculated for $C_{26}H_{24}NO$ 366.1852, observed 366.1848 (-1.1 ppm).

2.2.4. Fluorene monomer

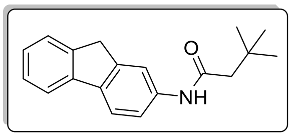

According to the general procedure, 36 mg (0.2 mmol) of 2-amino-fluorene, 56 µL (54 mg, 0.4 mmol) of *tert*-butyl acetyl chloride, 84 µL (61 mg, 0.6 mmol) of dry NEt_3 and 2 mL of dry CH_2Cl_2 yielded 56 mg (0.2 mmol, 279.38 g/mol, 99%) of **fluorene monomer** as an off-white solid.

Purification conditions: Column eluent (SiO_2): CH_2Cl_2/methanol gradient (99:1, 98:2).

R_f = 0.8 (SiO_2, CH_2Cl_2/methanol 98:2)

m.p.: 176 °C - 177 °C

^{1}H-NMR (500 MHz, $CDCl_3$): δ/ppm = 1.13 (s, 9H, $-CH_3$), 2.25 (s, 2H, $-CH_2-$), 3.89 (s, 2H, $-CH_2-$), 7.12 (s, 1H, NH), 7.25 - 7.29 (m, 1H, aromatic), 7.32 (dd, 1H, *J* = 8.1 Hz, 2.0 Hz, aromatic), 7.36 (t, 1H, *J* = 7.5 Hz, aromatic), 7.52 (d, 1H, *J* = 7.5 Hz, aromatic), 7.70 (d, 1H, *J* = 8.1 Hz, aromatic), 7.72 (dd, 1H, *J* = 7.5 Hz, 2.0 Hz, aromatic), 7.93 (s, 1H, aromatic).

^{13}C-NMR (126 MHz, $CDCl_3$): δ/ppm = 30.0 (3 $-CH_3$), 31.5 (1 C), 37.2 (1 $-CH_2-$), 51.9 (1 $-CH_2-$), 117.1 (1 CH aromatic), 118.7 (1 CH aromatic), 119.7 (1 CH aromatic), 120.2 (1 CH aromatic), 125.1 (1 CH aromatic), 126.4 (1 CH aromatic), 126.9 (1 CH aromatic), 136.8 (1 C aromatic), 138.2 (1 C aromatic), 141.5 (1 C aromatic), 143.3 (1 C aromatic), 144.5 (1 C aromatic), 170.2 (1 C=O).

IR (neat): $\tilde{\nu}/cm^{-1}$ 3283, 2951, 2862, 1650, 1614, 1596, 1532, 1460, 1416, 1364, 1346, 1327, 1307, 1256, 1232, 1201, 1143, 1127, 876, 826, 710.

HR MS (ESI) $[M+H]^+$ *m/z* calculated for $C_{19}H_{22}NO$ 280.1696, observed 280.1686 (-3.7 ppm).

2.2.5. NMI monomer

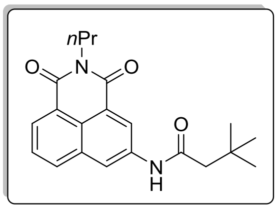

According to the general procedure, 51 mg (0.2 mmol) of 3-amino-NMI, 56 µL (54 mg, 0.4 mmol) of *tert*-butyl acetyl chloride, 84 µL (61 mg, 0.6 mmol) of dry NEt_3 and 2 mL of dry CH_2Cl_2 yielded 69 mg (0.2 mmol, 352.43 g/mol, 98%) of **NMI monomer** as an off-white solid.
Purification conditions: Column eluent (SiO_2): CH_2Cl_2/methanol (99:1).

R_f = 0.7 (SiO_2, CH_2Cl_2/methanol 98:2)
m.p.: 226 °C - 227 °C
^{1}H-NMR (500 MHz, $CDCl_3$): δ/ppm = 1.01 (t, 3H, *J* = 7.4 Hz, -CH_3), 1.16 (s, 9H, -CH_3), 1.72 - 1.79 (m, 2H, -CH_2-), 2.34 (s, 2H, -CH_2-), 4.12 - 4.15 (m, 2H, -CH_2-), 7.42 (s, 1H, NH), 7.72 (dd, 1H, *J* = 8.2 Hz, 7.3 Hz, aromatic), 8.16 (dd, 1H, *J* = 8.2 Hz, 1.1 Hz, aromatic), 8.22 (d, 1H, *J* = 2.2 Hz, aromatic), 8.49 (dd, 1H, *J* = 7.3 Hz, 1.1 Hz, aromatic), 8.99 (d, 1H, *J* = 2.2 Hz, aromatic).
^{13}C-NMR (126 MHz, $CDCl_3$): δ/ppm = 11.7 (1 -CH_3), 21.5 (1 -CH_2-), 30.0 (3 -CH_3), 31.7 (1 C), 42.2 (1 -CH_2-), 51.9 (1 -CH_2-), 122.4 (1 CH aromatic), 122.6 (1 C aromatic), 123.6 (1 C aromatic), 124.0 (1 CH aromatic), 125.3 (1 C aromatic), 127.7 (1 CH aromatic), 130.1 (1 CH aromatic), 132.8 (1 C aromatic), 133.9 (1 CH aromatic), 136.5 (1 C aromatic), 164.0 (1 C=O), 164.3 (1 C=O), 170.8 (1 C=O).
IR (neat): $\tilde{\nu}$/cm^{-1} 3247, 3072, 2965, 2870, 1699, 1653, 1627, 1596, 1534, 1503, 1463, 1423, 1376, 1338, 1271, 1235, 1124, 1072, 903, 782, 739, 706, 658.
HR MS (ESI) $[M+H]^+$ *m/z* calculated for $C_{21}H_{25}N_2O_3$ 353.1860, observed 353.1855 (-1.3 ppm).

2.3. Synthesis of polyether macrocycles **16C4** and **18C4**

Polyether macrocycle **18C6** was synthesized according to previously reported literature.[4] This procedure was adapted to the synthesis of macrocycles **16C4** and **18C4** (See Scheme S1 below).

Scheme S1. Synthesis of Unsaturated Macrocycles **18C6**, **18C4** and **16C4**

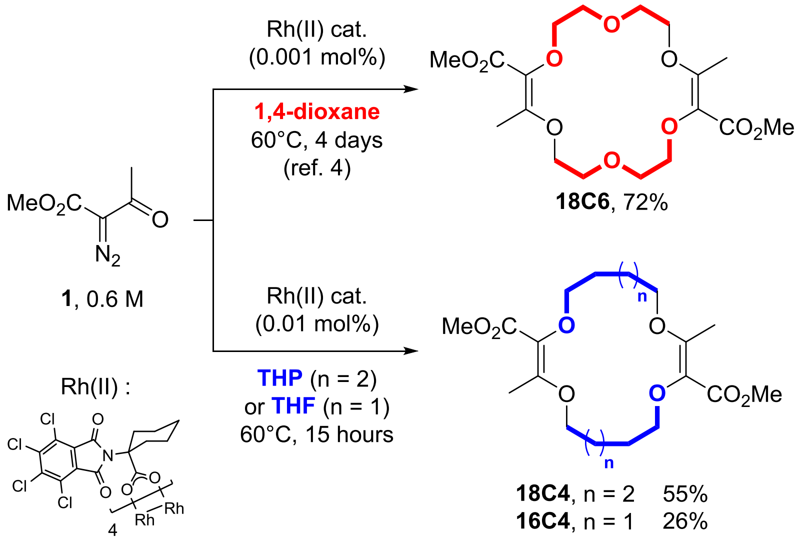

2.3.1. Synthesis of polyether macrocycle **16C4**

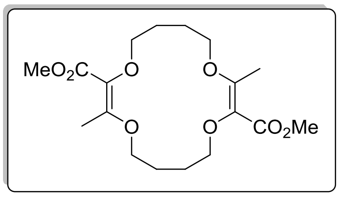

In a 5 mg vial, 1.42 mg (0.0007 mmol, 0.0001 equiv) of $Rh_2(TCPTCC)_4$ was dissolved in 1.0 mL of dry THF. In a flame dried Schlenk, adapted with a condenser, under nitrogen atmosphere, was introduced the rhodium solution and the vial was washed with 0.7 mL of dry THF. Then, 10.0 mL of THF (for a total of 11.7 mL) were added, followed by α-diazo-β-keto-methyl-ester **1** (1.00 g, 7 mmol, 1 equiv, c = 0.6 M). The solution was heated at 60 °C for 15 hours. The completion of the reaction was followed by TLC analysis and infrared spectroscopy (2146 cm^{-1}). The solvent was removed under reduced pressure. The mixture was filtered through a pad of neutral alumina (solid deposit,[5] EtOAc/CH_2Cl_2 1:1) and the solvent was evaporated. The residual solid was dissolved in 4 mL of CH_2Cl_2 and 100 mL of pentane were added to precipitate the macrocycle. The macrocycle was filtered on a filtration funnel and washed with 100 mL of Et_2O/pentane (1:9) to yield 338 mg (0.91 mmol, 372.41 g/mol, 26%) of **16C4** as a white solid.

R_f = 0.79 (SiO_2, CH_2Cl_2/methanol 90:10)
m.p.: 129 °C - 132 °C
^{1}H-NMR (500 MHz, $CDCl_3$): δ/ppm = 1.78 - 1.84 (m, 4H, $-CH_2-$), 1.89 - 1.95 (m, 4H, $-CH_2-$), 2.38 (s, 6H, $-CH_3$), 3.73 (t, 4H, J = 7.2 Hz, $-CH_2-$), 3.75 (s, 6H, $-CH_3$), 4.00 (t, 4H, J = 5.8 Hz, $-CH_2-$).
^{13}C-NMR (126 MHz, $CDCl_3$): δ/ppm = 14.4 (2 $-CH_3$), 26.2 (2 $-CH_2-$), 26.8 (2 $-CH_2-$), 51.5 (2 $-CH_3$), 67.7 (2 $-CH_2-$), 73.6 (2 $-CH_2-$), 130.1 (2 =C), 158.7 (2 =C), 166.9 (2 C=O).
IR (neat): ṽ/cm^{-1} 2944, 2886, 1689, 1604, 1439, 1379, 1304, 1266, 1207, 1190, 1157, 1083, 1034, 1013, 972, 776, 631.
HR MS (ESI) $[M+H]^+$ *m/z* calculated for $C_{18}H_{29}O_8$ 373.1857, observed 373.1856 (-0.2 ppm).

2.3.2. Synthesis of polyether macrocycle **18C4**

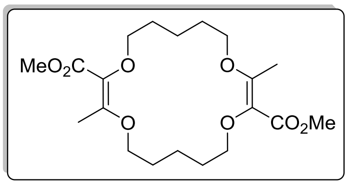

In a 3 mL vial, 1.42 mg (0.0007 mmol, 0.0001 equiv) of $Rh_2(TCPTCC)_4$ was dissolved in 1.0 mL of dry tetrahydropyran (THP). In a flame dried Schlenk, adapted with a condenser, under nitrogen atmosphere, was introduced the rhodium solution and the vial was washed with 0.7 mL of dry THP. Then, 10.0 mL of THP (for a total of 11.7 mL) were added, followed by α-diazo-β-keto-methyl-ester **1** (1.00 g, 7 mmol, 1 equiv, c = 0.6 M). The solution was heated at 60 °C for 15 hours. The completion of the reaction was followed by TLC analysis and infrared spectroscopy (2146 cm^{-1}). The solvent was removed under reduced pressure. The residual solid was dissolved in 3 mL of CH_2Cl_2 and 100 mL of pentane were added to precipitate the macrocycle. The macrocycle was filtered and washed with 100 mL of Et_2O/pentane (1:9) to yield 771 mg (1.93 mmol, 400.47 g/mol, 55%) of **18C4** as a white solid.

$\mathbf{R_f}$ = 0.83 (SiO_2, CH_2Cl_2/methanol 90:10)
m.p.: 95 °C - 96 °C
^{1}H-NMR (500 MHz, $CDCl_3$): δ/ppm = 1.65 - 1.76 (m, 12H, $-CH_2-$), 2.36 (s, 6H, $-CH_3$), 3.68 - 3.71 (m, 4H, $-CH_2-$), 3.74 (s, 6H, $-CH_3$), 3.99 - 4.01 (m, 4H, $-CH_2-$).
^{13}C-NMR (126 MHz, $CDCl_3$): δ/ppm = 14.4 (2 $-CH_3$), 23.4 (2 $-CH_2-$), 29.8 (2 $-CH_2-$), 30.1 (2 $-CH_2-$), 51.5 (2 $-CH_3$), 67.9 (2 $-CH_2-$), 72.8 (2 $-CH_2-$), 129.8 (2 =C), 158.7 (2 =C), 167.0 (2 C=O).
IR (neat): $\tilde{v}$/cm^{-1} 2939, 1700, 1607, 1461, 1435, 1403, 1374, 1285, 1248, 1191, 1162, 1124, 1082, 1020, 1003, 971, 932, 885, 774, 732, 688, 620, 558.
HR MS (ESI) $[M+H]^+$ *m/z* calculated for $C_{20}H_{33}O_8$ 401.2170, observed 401.2176 (1.5 ppm).

2.4. Synthesis of functionalized macrocycles

2.4.1. General procedure for the synthesis of macrocycles

In a one neck flask under nitrogen atmosphere, dry tetrahydrofuran (c = 0.1 M) was added to 1 equivalent of polyether macrocycle (**18C6, 18C4** or **16C4**) and 3 equivalents of aromatic amine. The mixture was cooled down to -100 °C (EtOH/liquid nitrogen bath). Then 4 equivalents of freshly sublimed *t*-BuOK were added in one portion. After stirring for 1-2 minutes at -100 °C, the cooling bath was removed and the reaction was allowed to reach 25 °C on its own and stirred for an additional 3 hours. The conversion was followed by TLC analysis and LR-ESI-MS. Upon completion, the reaction was quenched by adding a few drops of methanol and directly purified by column chromatography (SiO_2) without further treatment. A second column chromatography (Al_2O_3, neutral) could be required. Finally, the resulting oil or solid was purified by selective precipitation (dissolution in a minimal amount of CH_2Cl_2 or ethyl acetate required for solubility, followed by addition of a large excess of pentane) affording the desired chiral polyether macrocycle.

Pyrene-18C6, **pyrene-18C4** and **pyrene-16C4** were synthesized according to the previously reported procedure.[6]

2.4.2. Perylene-18C6

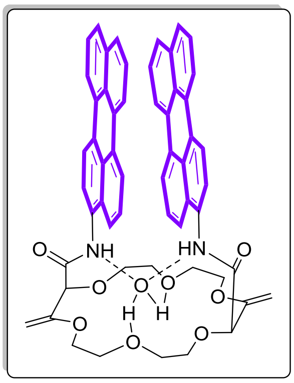

According to the general procedure, 100 mg (0.25 mmol) of **18C6**, 200 mg (0.75 mmol) of 3-amino-perylene, 2 mL of dry THF and 112 mg (1.0 mmol) of *t*-BuOK yielded 130 mg (0.15 mmol, 893.01 g/mol, 60%) of **perylene-18C6** (water adduct) as a yellow-orange solid.

Purification conditions:

Column eluent (SiO_2): CH_2Cl_2, then CH_2Cl_2/methanol gradient (98:2, 95:5, 90:10).

Precipitation: CH_2Cl_2 then pentane

R_f = 0.3 (SiO_2, CH_2Cl_2/methanol 95:5)

m.p.: 235 °C - 240 °C

^{1}H-NMR (500 MHz, $CDCl_3$): δ/ppm = 3.72 (m, 2H, -CH_2-), 3.80 (m, 2H, -CH_2-), 3.87 (m, 2H, -CH_2-), 3.95 - 4.13 (m, 10H, -CH_2-), 4.41 (d, 2H, *J* = 2.6 Hz, =CH_2), 4.47 (s, 2H, -CH-), 4.52 (d, 2H, *J* = 2.6 Hz, =CH_2), 7.04 - 7.11 (m, 6H, aromatics), 7.27 (d, 2H, *J* = 8.0 Hz, aromatics), 7.30 (d, 2H, *J* = 8.0 Hz, aromatics), 7.47 (d, 2H, *J* = 8.3 Hz, aromatics), 7.49 (d, 2H, *J* = 7.5 Hz, aromatics), 7.55 (d, 2H, *J* = 7.4 Hz, aromatics), 7.61 (d, 2H, *J* = 7.4 Hz, aromatics), 7.72 (d, 2H, *J* = 8.2 Hz, aromatics), 7.87 (d, 2H, *J* = 8.2 Hz, aromatics), 9.17 (s, 2H, NH).

^{13}C-NMR (126 MHz, $CDCl_3$): δ/ppm = 67.3 (2 -CH_2-), 68.7 (2 -CH_2-), 68.8 (2 -CH_2-), 70.3 (2 -CH_2-), 83.4 (2 -CH-), 89.7 (2 =CH_2), 119.5 (4 CH aromatics), 119.6 (2 CH aromatics), 119.7 (2 CH aromatics), 119.9 (2 CH aromatics), 120.2 (2 CH aromatics), 126.0 (2 CH aromatics), 126.1 (2 CH aromatics), 126.3 (2 CH aromatics), 127.0 (2 CH aromatics), 127.1 (2 C aromatics), 127.5 (2 CH aromatics), 127.8 (2 C aromatics), 128.3 (2 C aromatics), 128.5 (2 C aromatics), 130.6 (4 C aromatics), 131.0 (2 C aromatics), 131.4 (2 C aromatics), 134.2 (2 C aromatics), 156.3 (2 =C), 167.2 (2 C=O).

IR (neat): $\tilde{\nu}$/cm^{-1} 3366, 2924, 2874, 1693, 1542, 1509, 1389, 1324, 1288, 1099, 996, 919, 807, 765, 731.

HR MS (ESI) $[M+H]^+$ *m/z* calculated for $C_{56}H_{47}N_2O_8$ 875.3327, observed 875.3341 (1.6 ppm).

2.4.3. Perylene-18C4

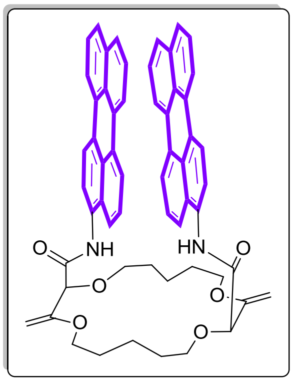

According to the general procedure, 50 mg (0.125 mmol) of **18C4**, 100 mg (0.375 mmol) of 3-amino-perylene, 1.5 mL of dry THF and 56 mg (0.5 mmol) of *t*-BuOK yielded 56 mg (0.064 mmol, 871.03 g/mol, 51%) of **perylene-18C4** as a yellow-brown solid.

Purification conditions:

Column 1 eluent (SiO_2): CH_2Cl_2, then CH_2Cl_2/methanol gradient (99:1, 98:2, 96:4).

Column 2 eluent (SiO_2): CH_2Cl_2, then CH_2Cl_2/methanol gradient (99.5:0.5, 99:1, 98:2).

Precipitation: CH_2Cl_2 then pentane

R_f = 0.4 (SiO_2, CH_2Cl_2/methanol 98:2)

m.p.: 246 °C - 248 °C

^{1}H-NMR (500 MHz, $CDCl_3$): δ/ppm = 1.54 (m, 2H, -CH_2-), 1.76 (m, 2H, -CH_2-), 1.84 (m, 2H, -CH_2-), 1.97 - 2.07 (m, 4H, -CH_2-), 2.40 (m, 2H, -CH_2-), 3.62 - 3.74 (m, 6H, -CH_2-), 4.02 (dt, 2H, *J* = 8.4 Hz, 3.8 Hz, -CH_2-), 4.37 (d, 2H, *J* = 2.4 Hz, =CH_2), 4.41 - 4.43 (m, 4H, -CH- and =CH_2), 6.98 (d, 2H, *J* = 7.7 Hz, aromatics), 7.18 (d, 2H, *J* = 7.4 Hz, aromatics), 7.23 (m, 2H, aromatics), 7.30 - 7.33 (m, 4H, aromatics), 7.36 (d, 2H, *J* = 8.1 Hz, aromatics), 7.40 (d, 2H, *J* = 8.1 Hz, aromatics), 7.43 - 7.47 (m, 4H, aromatics), 7.64 (d, 2H, *J* = 7.3 Hz, aromatics), 7.81 (d, 2H, *J* = 7.4 Hz, aromatics), 8.37 (s, 2H, NH).

^{13}C-NMR (126 MHz, $CDCl_3$): δ/ppm = 25.2 (2 -CH_2-), 29.8 (2 -CH_2-), 31.2 (2 -CH_2-), 68.5 (2 -CH_2-), 68.6 (2 -CH_2-), 83.3 (2 -CH-), 89.4 (2 -CH_2-), 119.5 (2 CH aromatics), 119.7 (4 CH aromatics), 120.0 (2 CH aromatics), 120.2 (2 CH aromatics), 122.3 (2 CH aromatics), 126.0 (2 CH aromatics), 126.1 (2 CH aromatics), 126.2 (2 CH aromatics), 127.1 (2 CH aromatics), 127.75 (2 CH aromatics), 127.82 (2 C aromatics), 128.5 (2 C aromatics), 128.6 (2 C aromatics), 128.9 (2 C aromatics), 130.2 (2 C aromatics), 130.7 (2 C aromatics), 130.8 (2 C aromatics), 131.4 (2 C aromatics), 134.2 (2 C aromatics), 156.3 (2 =C), 167.5 (2 C=O).

IR (neat): ṽ/cm^{-1} 3404, 2922, 2866, 1698, 1621, 1533, 1504, 1473, 1428, 1389, 1324, 1282, 1095, 1079, 1060, 994, 821, 806, 763.

HR MS (ESI) $[M+H]^+$ *m/z* calculated for $C_{58}H_{51}N_2O_6$ 871.3742, observed 871.3758 (1.8 ppm).

2.4.4. Perylene-16C4

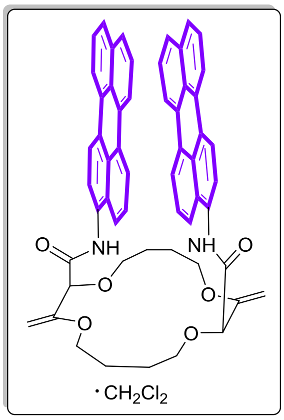

According to the general procedure, 47 mg (0.125 mmol) of **16C4**, 100 mg (0.375 mmol) of 3-amino-perylene, 1.5 mL of dry THF and 56 mg (0.5 mmol) of *t*-BuOK yielded 55 mg (0.059 mmol, 927.92 g/mol, 47%) of **perylene-16C4** (CH_2Cl_2 adduct) as a greenish-brown solid.

Purification conditions:

Column 1 eluent (SiO_2): CH_2Cl_2, then CH_2Cl_2/methanol gradient (98:2, 95:5, 90:10).

Column 2 eluent (SiO_2): CH_2Cl_2, then CH_2Cl_2/EtOAc gradient (98:2, 96:4, 94:6, 92:8).

Precipitation: CH_2Cl_2 then pentane

R_f = 0.4 (SiO_2, CH_2Cl_2/methanol 98:2)

m.p.: 249 °C - 250 °C

^{1}H-NMR (500 MHz, $CDCl_3$): δ/ppm = 1.79 - 1.90 (m, 4H, $-CH_2-$), 2.06 - 2.17 (m, 4H, $-CH_2-$), 3.64 (m, 2H, $-CH_2-$), 3.70 - 3.74 (m, 4H, $-CH_2-$), 3.93 (m, 2H, $-CH_2-$), 4.42 (d, 2H, *J* = 2.4 Hz, $=CH_2$), 4.46 (s, 2H, -CH-), 4.48 (d, 2H, *J* = 2.4 Hz, $=CH_2$), 7.37 - 7.44 (m, 6H, aromatics), 7.60 - 7.67 (m, 6H, aromatics), 8.02 - 8.10 (m, 6H, aromatics), 8.15 (s, 4H, aromatics), 9.06 (s, 2H, NH).

^{13}C-NMR (126 MHz, $CDCl_3$): δ/ppm = 24.5 (2 $-CH_2-$), 25.8 (2 $-CH_2-$), 66.5 (2 $-CH_2-$), 67.2 (2 $-CH_2-$), 82.7 (2 -CH-), 89.7 (2 $-CH_2-$), 119.8 (2 CH aromatics), 120.1 (2 CH aromatics), 120.2 (2 CH aromatics), 120.5 (4 CH aromatics), 120.6 (2 CH aromatics), 126.6 (2 CH aromatics), 126.8 (2 CH aromatics), 126.9 (2 CH aromatics), 127.6 (2 CH aromatics), 128.2 (2 CH + 2 C aromatics), 128.5 (2 C aromatics), 128.6 (2 C aromatics), 129.3 (2 C aromatics), 131.0 (2 C aromatics), 131.1 (2 C aromatics), 131.6 (2 C aromatics), 132.1 (2 C aromatics), 134.7 (2 C aromatics), 156.5 (2 =C), 167.2 (2 C=O).

IR (neat): $\tilde{\nu}/cm^{-1}$ 3401, 3377, 3052, 2914, 2873, 1689, 1627, 1591, 1535, 1501, 1387, 1293, 1095, 1030, 952, 829, 808, 767, 728, 690.

HR MS (ESI) $[M+H]^+$ *m/z* calculated for $C_{56}H_{47}N_2O_6$ 843.3429, observed 843.3423 (-0.6 ppm).

2.4.5. Fluorene-18C6

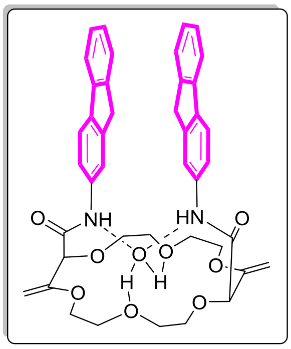

According to the general procedure, 101 mg (0.25 mmol) of **18C6**, 136 mg (0.75 mmol) of 2-amino-fluorene, 2.5 mL of dry and degassed THF and 112 mg (1.00 mmol) of *t*-BuOK yielded 52 mg (0.07 mmol, 720.82 g/mol, 38%) of **fluorene-18C6** (water adduct) as a yellow solid.

Purification conditions:

Column eluent (SiO_2): CH_2Cl_2/methanol gradient (100:0, 95:5, 90:10).

Precipitation: CH_2Cl_2then pentane

$\mathbf{R_f}$ = 0.5 (SiO_2, CH_2Cl_2/methanol 9:1)

m.p.: 104 °C - 108 °C

^{1}**H-NMR** (500 MHz, $CDCl_3$): δ/ppm = 3.58 - 3.66 (m, 5H, -CH_2-), 3.67 - 3.76 (m, 5H, -CH_2-), 3.79 - 3.83 (m, 2H, -CH_2-), 3.87 - 3.96 (m, 6H, -CH_2-), 4.02 - 4.07 (m, 2H, -CH_2-), 4.33 (d, 2H, *J* = 2.7 Hz, =CH_2), 4.39 (s, 2H, -CH-), 4.49 (d, 2H, *J* = 2.7 Hz, =CH_2), 7.15 - 7.22 (m, 4H, aromatics), 7.34 - 7.36 (m, 2H, aromatics), 7.44 - 7.46 (m, 4H, aromatics), 7.50 - 7.52 (m, 2H, aromatics), 7.94 (s, 2H, aromatics), 9.52 (s, 2H, NH).

^{13}**C-NMR** (126 MHz, $CDCl_3$): δ/ppm = 37.1 (2 -CH_2-), 66.9 (2 -CH_2-), 68.7 (2 -CH_2-), 69.2 (2 -CH_2-), 70.2 (2 -CH_2-), 83.4 (2 -CH-), 88.7 (2 =CH_2), 116.6 (2 CH aromatics), 118.6 (2 CH aromatics), 119.5 (2 CH aromatics), 120.1 (2 CH aromatics), 124.9 (2 CH aromatics), 126.1 (2 CH aromatics), 126.7 (2 CH aromatics), 137.0 (2 C aromatics), 137.9 (2 C aromatics), 141.4 (2 C aromatics), 143.3 (2 C aromatics), 144.2 (2 C aromatics), 156.7 (2 =C), 167.3 (2 C=O).

IR (neat): $\tilde{v}$/cm^{-1} 3316, 2921, 1681, 1593, 1534, 1492, 1457, 1424, 1352, 1290, 1094, 1074, 993, 948, 827, 766, 732, 702, 573.

HR MS (ESI) $[M+H]^+$ *m/z* calculated for $C_{42}H_{43}N_2O_8$ 703.3014, observed 703.3029 (2.1 ppm).

2.4.6. Fluorene-18C4

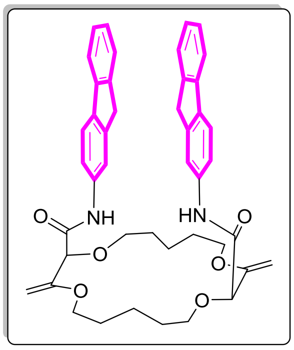

According to the general procedure, 100 mg (0.25 mmol) of **18C4**, 136 mg (0.75 mmol) of 2-amino-fluorene, 2.5 mL of dry and degassed THF and 112 mg (1.00 mmol) of *t*-BuOK yielded 33 mg (0.05 mmol, 698.86 g/mol, 19%) of **fluorene-18C4** as a yellow solid.

Purification conditions:

Column eluent (SiO_2): ethyl acetate (remaining aniline co-eluted with **fluorene-18C4**)

Precipitation: ethyl acetate then pentane (this step was repeated twice)

R_f = 0.7 (SiO_2, ethyl acetate)

m.p.: 176 °C - 177 °C

^{1}H-NMR (500 MHz, $CDCl_3$): δ/ppm = 1.66 - 1.84 (m, 12H, -CH_2-), 3.52 - 3.78 (m, 10H, -CH_2-), 3.88 - 3.92 (m, 2H, -CH_2-), 4.27 (s, 2H, -CH-), 4.33 (d, 2H, *J* = 2.4 Hz, =CH_2), 4.36 (d, 2H, *J* = 2.4 Hz, =CH_2), 7.21 - 7.26 (m, 4H, aromatics), 7.32 - 7.34 (m, 2H, aromatics), 7.40 - 7.42 (m, 2H, aromatics), 7.48 - 7.46 (m, 2H, aromatics), 7.51 - 7.53 (m, 2H, aromatics), 7.90 (s, 2H, aromatics), 8.53 (s, 2H, NH).

^{13}C-NMR (126 MHz, $CDCl_3$): δ/ppm = 24.4 (2 -CH_2-), 28.8 (2 -CH_2-), 30.0 (2 -CH_2-), 37.1 (2 -CH_2-), 68.2 (2 -CH_2-), 69.7 (2 -CH_2-), 82.9 (2 -CH-), 88.5 (2 -CH_2-), 116.7 (2 CH aromatics), 118.5 (2 CH aromatics), 119.7 (2 CH aromatics), 120.3 (2 CH aromatics), 125.0 (2 CH aromatics), 126.4 (2 CH aromatics), 126.8 (2 CH aromatics), 136.3 (2 C aromatics), 138.2 (2 C aromatics), 141.3 (2 C aromatics), 143.3 (2 C aromatics), 144.5 (2 C aromatics), 156.9 (2 =C), 167.3 (2 C=O).

IR (neat): ṽ/cm^{-1} 3386, 2921, 2870, 1693, 1637, 1590, 1524, 1492, 1459, 1424, 1298, 1217, 1089, 998, 1217, 1089, 998, 947, 823, 766, 733, 566.

HR MS (ESI) $[M+H]^+$ *m/z* calculated for $C_{44}H_{47}N_2O_6$ 699.3429, observed 699.3439 (1.4 ppm).

2.4.7. Fluorene-16C4

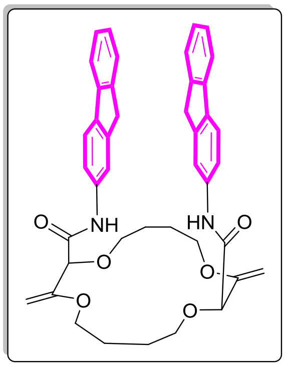

According to the general procedure, 93 mg (0.25 mmol) of **16C4**, 136 mg (0.75 mmol) of 2-amino-fluorene, 2.5 mL of dry and degassed THF and 112 mg (1.00 mmol) of *t*-BuOK yielded 59 mg (0.09 mmol, 670.81 g/mol, 35%) of **fluorene-16C4** as a yellow solid.

Purification conditions:

Column eluent (SiO_2): ethyl acetate (remaining aniline co-eluted with **fluorene-16C4**)

Precipitation: ethyl acetate then pentane (this step was repeated twice)

R_f = 0.6 (SiO_2, ethyl acetate)

m.p.: 159 °C - 161 °C

^{1}H-NMR (500 MHz, $CDCl_3$): δ/ppm = 1.69 - 1.75 (m, 4H, -CH_2-), 1.94 - 2.01 (m, 4H, -CH_2-), 3.53 - 3.66 (m, 6H, -CH_2-), 3.80 - 3.89 (m, 6H, -CH_2-), 4.30 (s, 2H, -CH-), 4.35 (d, 2H, *J* = 2.4 Hz, =CH_2), 4.39 (d, 2H, *J* = 2.4 Hz, =CH_2), 7.26 - 7.29 (m, 2H, aromatics), 7.34 - 7.37 (m, 2H, aromatics), 7.47 - 7.53 (m, 4H, aromatics), 7.71 - 7.73 (m, 4H, aromatics), 7.98 (s, 2H, aromatics), 8.59 (s, 2H, NH).

^{13}C-NMR (126 MHz, $CDCl_3$): δ/ppm = 24.2 (2 -CH_2-), 25.5 (2 -CH_2-), 37.2 (2 -CH_2-), 66.4 (2 -CH_2-), 67.6 (2 -CH_2-), 82.3 (2 -CH-), 89.3 (2 =CH_2), 116.7 (2 CH aromatics), 118.6 (2 CH aromatics), 119.7 (2 CH aromatics), 120.2 (2 CH aromatics), 125.1 (2 CH aromatics), 126.5 (2 CH aromatics), 126.9 (2 CH aromatics), 136.4 (2 C aromatics), 138.3 (2 C aromatics), 141.5 (2 C aromatics), 143.3 (2 C aromatics), 144.5 (2 C aromatics), 156.6 (2 =C), 166.9 (2 C=O).

IR (neat): $\tilde{\nu}$/cm^{-1} 3362, 3048, 2888, 1685, 1620, 1591, 1529, 1492, 1458, 1425, 1351, 1290, 1211, 1093, 1037, 951, 815, 762, 724, 570.

HR MS (ESI) $[M+H]^+$ *m/z* calculated for $C_{42}H_{43}N_2O_6$ 671.3116, observed 671.3116 (0.1 ppm).

2.4.8. NMI-18C6

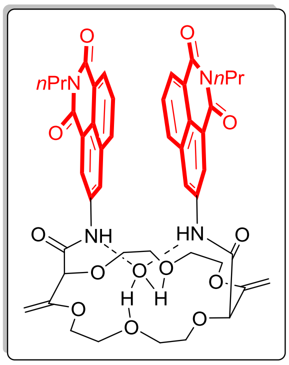

According to the general procedure, 101 mg (0.25 mmol) of **18C6**, 191 mg (0.75 mmol) of 3-amino-NMI, 2.5 mL of dry THF and 112 mg (1.00 mmol) of *t*-BuOK yielded 37 mg (0.043 mmol, 866.92 g/mol, 17%) of **NMI-18C6** (water adduct) as a yellow solid.

Purification conditions:

Column 1 eluent (SiO_2): CH_2Cl_2, then CH_2Cl_2/methanol gradient (98:2, 95:5, 90:10).

Column 2 eluent (Al_2O_3, neutral): CH_2Cl_2 for the packing of the column, then CH_2Cl_2/methanol gradient (99:1, 98:2, 97:3).

Preparative TLC (SiO_2): CH_2Cl_2/methanol (95:5)

R_f = 0.5 (SiO_2, CH_2Cl_2/methanol 9:1)

m.p.: 213 °C - 218 °C

^{1}H-NMR (500 MHz, $CDCl_3$): δ/ppm = 0.92 (t, 6H, *J* = 7.4 Hz, -CH_3), 1.54 - 1.62 (m, 4H, -CH_2-), 3.64 - 3.69 (m, 4H, -CH_2-), 3.73 - 3.77 (m, 2H, -CH_2-), 3.82 - 3.98 (m, 8H, -CH_2-), 4.04 - 4.07 (m, 2H, -CH_2-), 4.13 - 4.23 (m, 4H, -CH_2-), 4.40 (d, 2H, *J* = 2.8 Hz, =CH_2), 4.41 (s, 2H, -CH-), 4.51 (d, 2H, *J* = 2.8 Hz, =CH_2), 7.48 (dd, 2H, *J* = 8.3 Hz, 7.2 Hz, aromatics), 7.79 (dd, 2H, *J* = 8.3 Hz, 1.1 Hz, aromatics), 8.13 (d, 2H, *J* = 2.1 Hz, aromatics), 8.16 (dd, 2H, *J* = 7.2 Hz, 1.1 Hz, aromatics), 8.67 (d, 2H, *J* = 2.1 Hz, aromatics), 9.85 (s, 2H, NH).

^{13}C-NMR (126 MHz, $CDCl_3$): δ/ppm = 11.5 (2 -CH_3), 21.2 (2 -CH_2-), 41.8 (2 -CH_2-), 65.7 (2 -CH_2-), 68.7 (2 -CH_2-), 69.0 (2 -CH_2-), 69.1 (2 -CH_2-), 83.1 (2 -CH-), 88.9 (2 =CH_2), 121.4 (2 CH aromatics), 121.8 (2 C aromatics), 122.7 (2 C aromatics), 123.8 (2 CH aromatics), 124.4 (2 C aromatics), 126.9 (2 CH aromatics), 129.4 (2 CH aromatics), 131.9 (2 C aromatics), 133.2 (2 CH aromatics), 136.5 (2 C aromatics), 156.5 (2 C=), 163.0 (2 C=O), 163.6 (2 C=O), 168.0 (2 C=O).

IR (neat): $\tilde{\nu}$/cm^{-1} 3315, 2926, 1691, 1658, 1592, 1537, 1463, 1427, 1377, 1336, 1293, 1233, 1130, 1090, 1073, 991, 926, 885, 930, 778, 737, 666.

HR MS (ESI) $[M+H]^+$ *m/z* calculated for $C_{46}H_{49}N_4O_{12}$ 849.3342, observed 849.3340 (-0.2 ppm).

2.5. Monomeric amides NMR spectra

2.5.1. Pyrene monomer

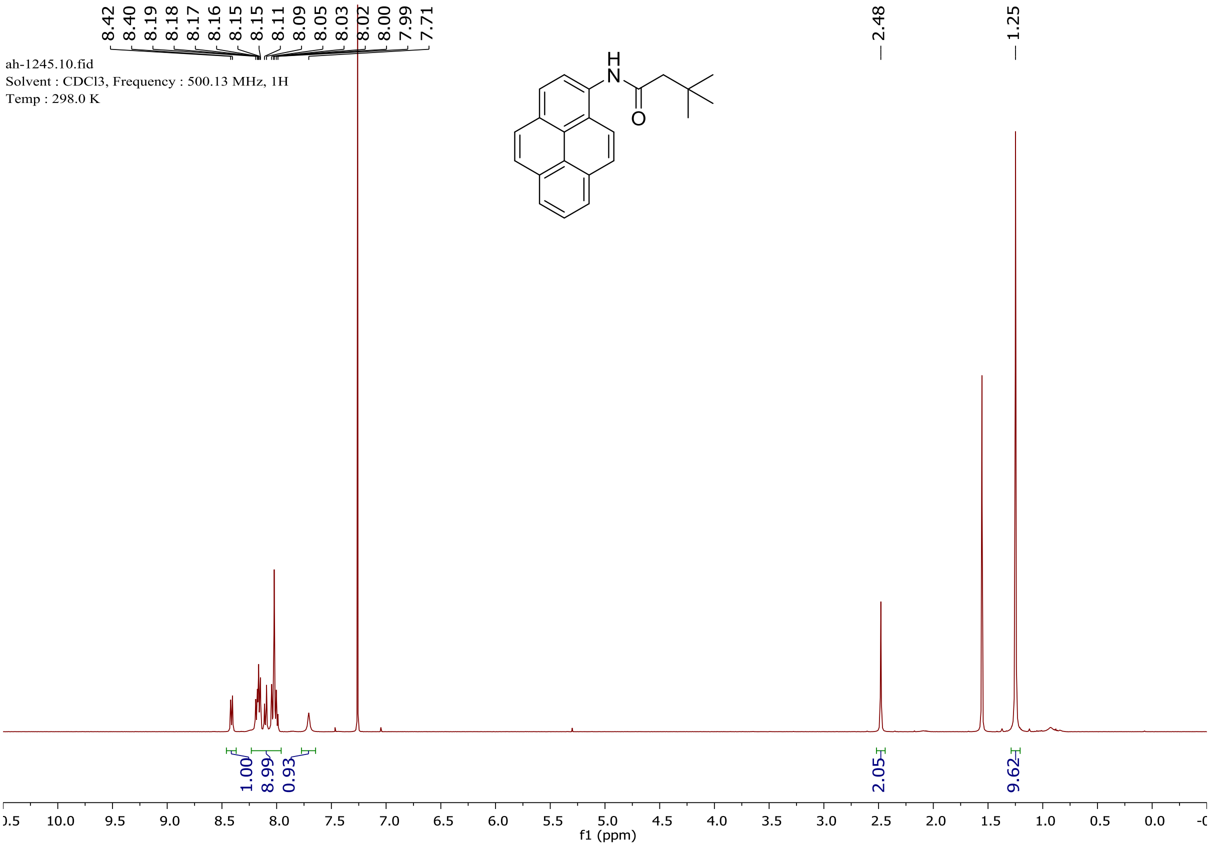

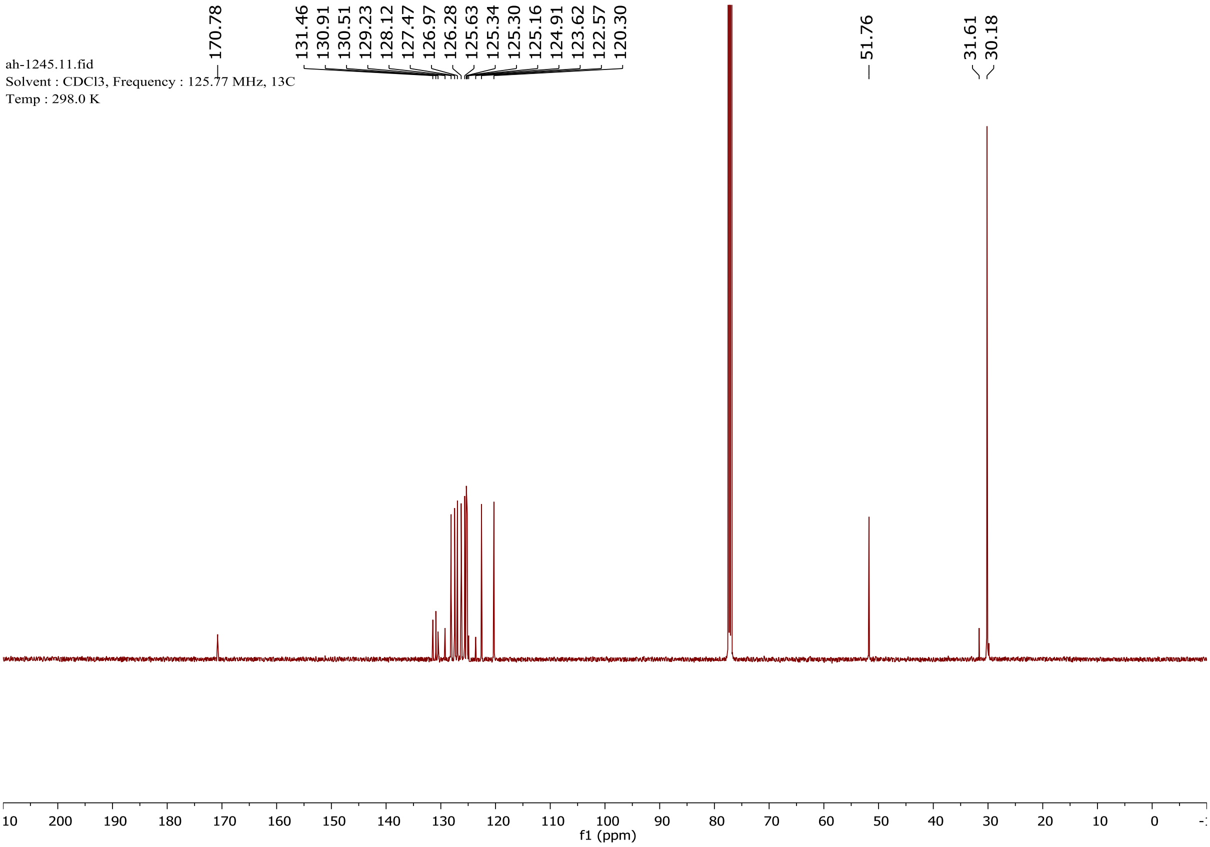

2.5.2. Perylene monomer

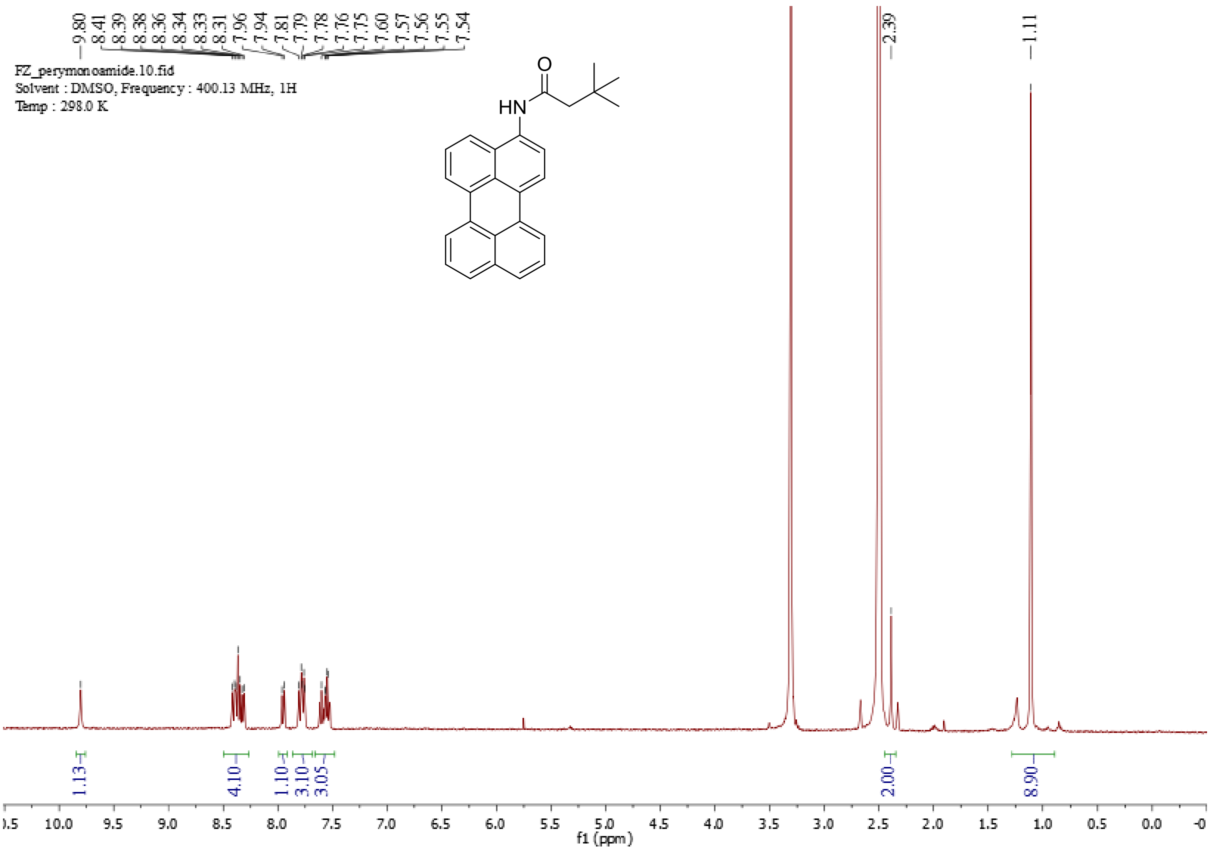

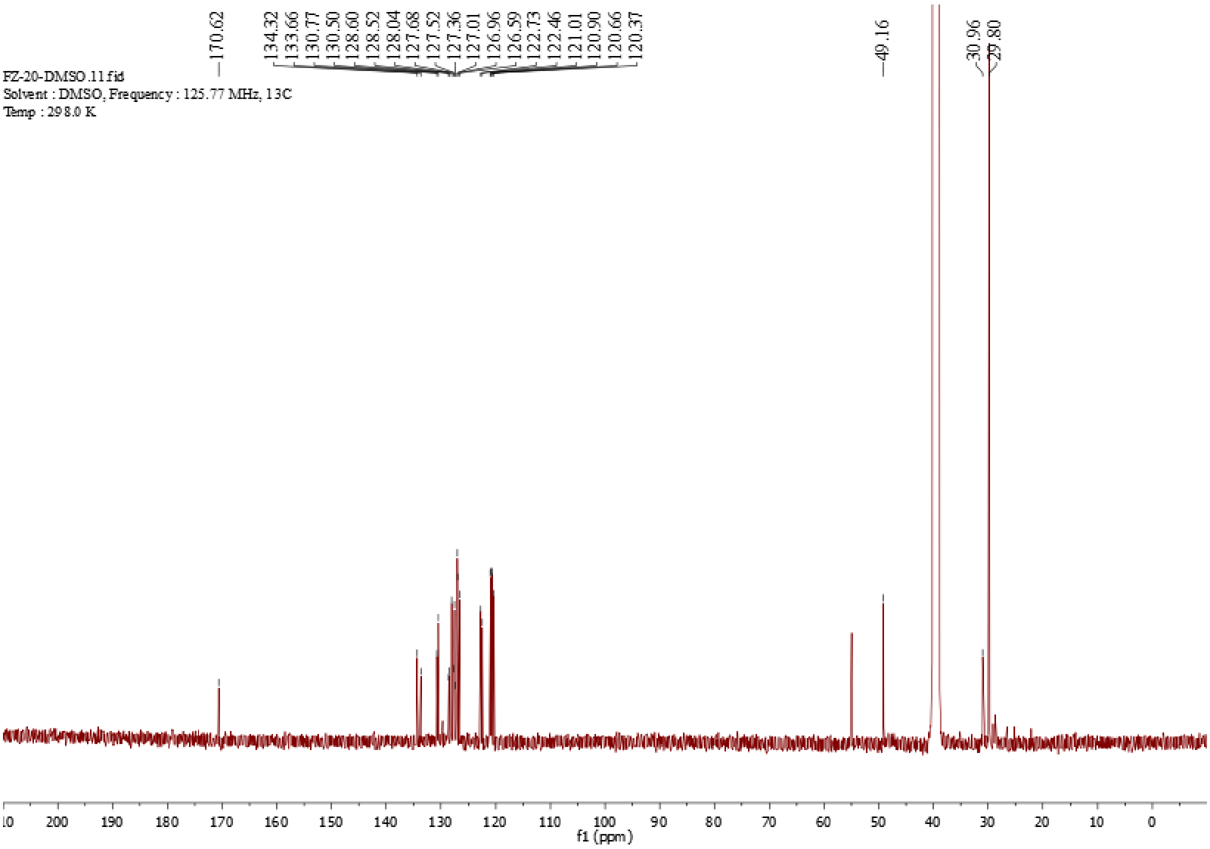

2.5.3. Fluorene monomer

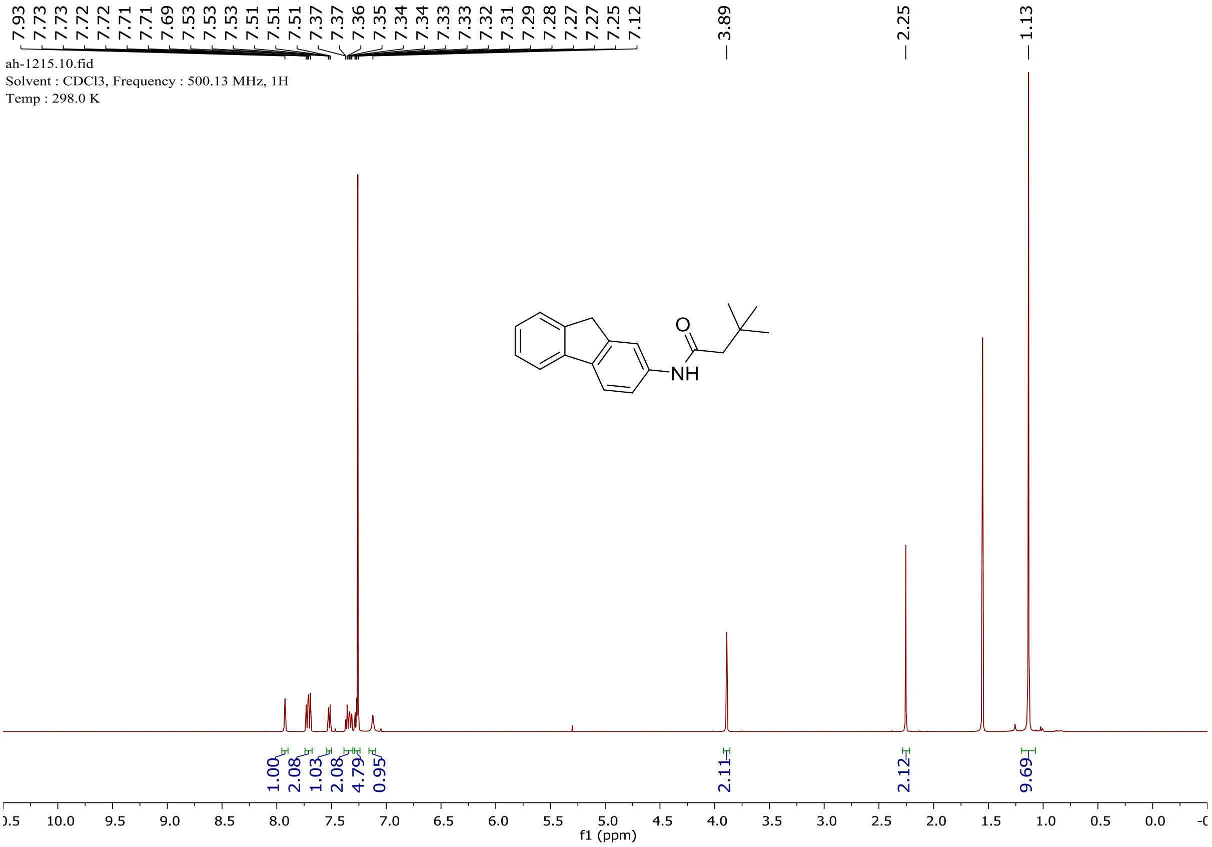

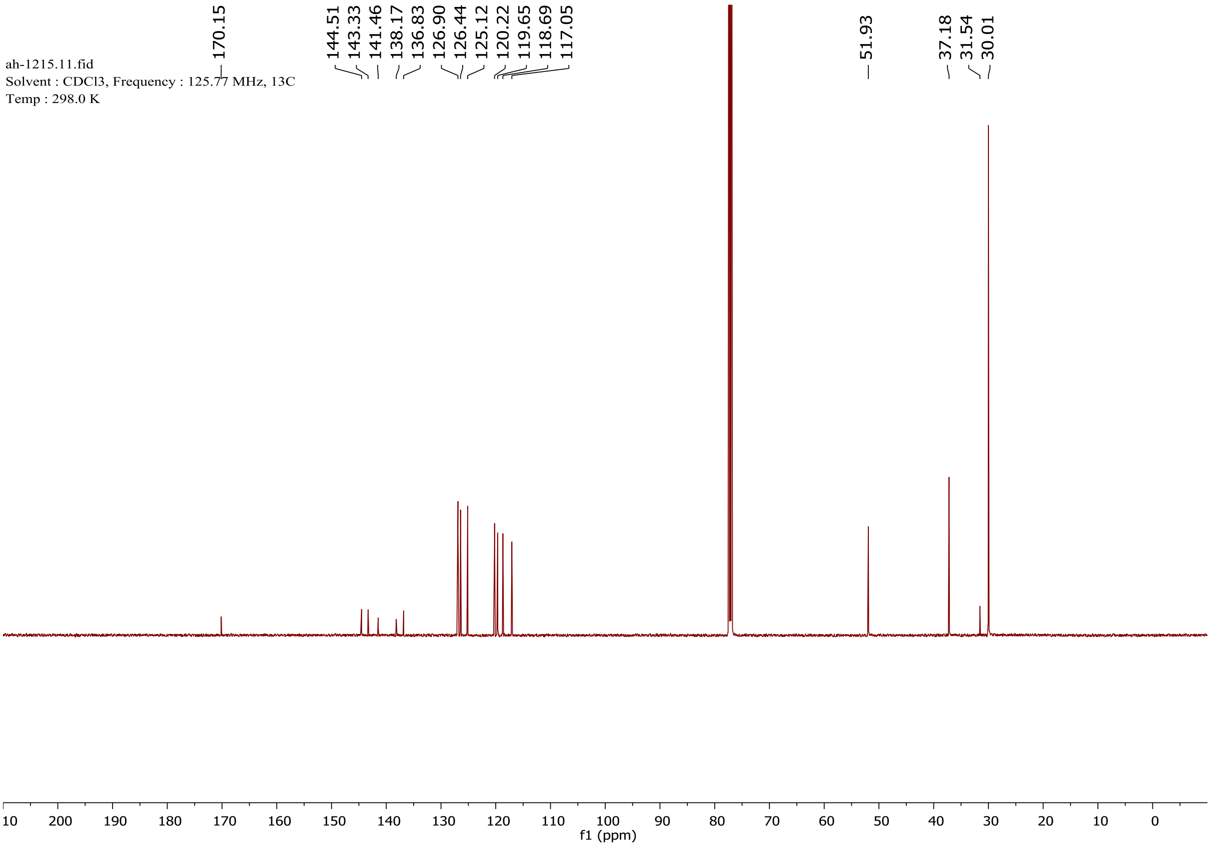

2.5.4. NMI monomer

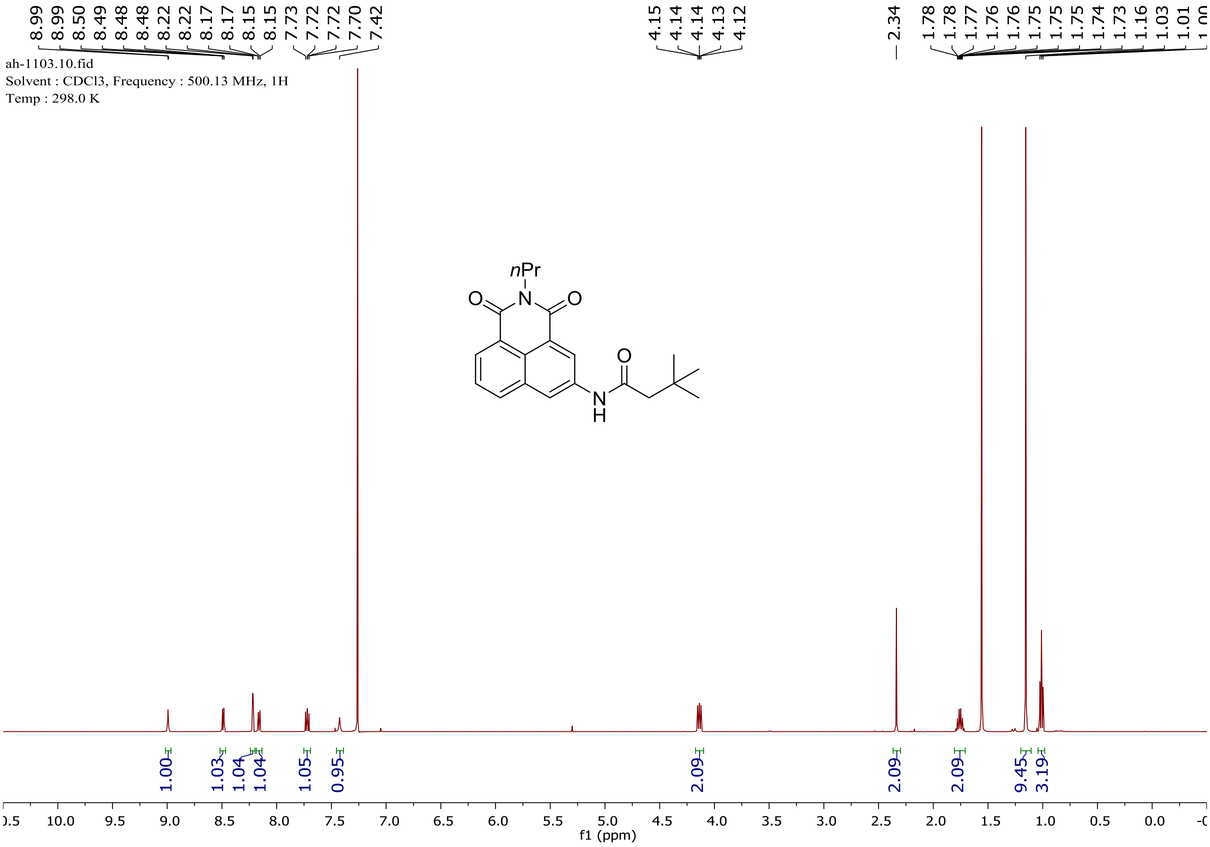

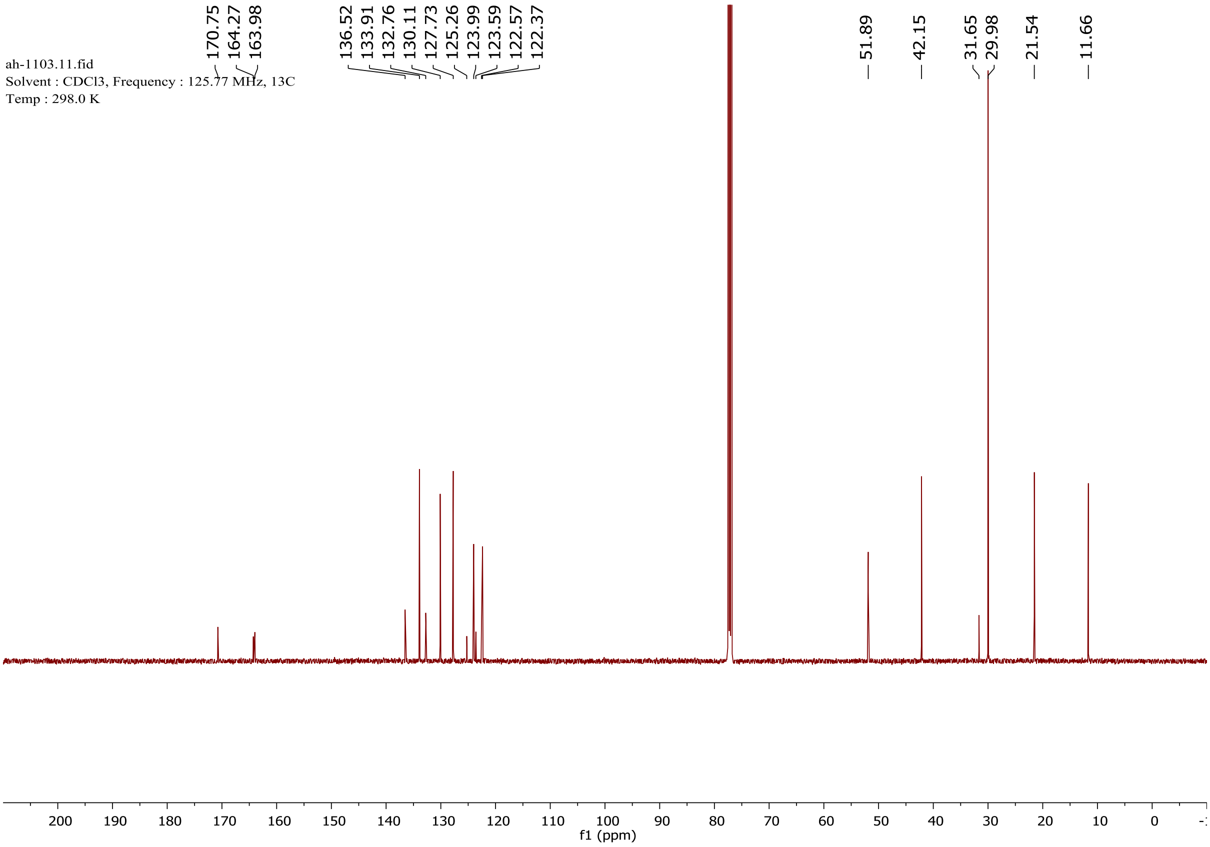

2.6. Polyether macrocycles **16C4** and **18C4** NMR spectra

2.6.1. Polyether macrocycles **16C4**

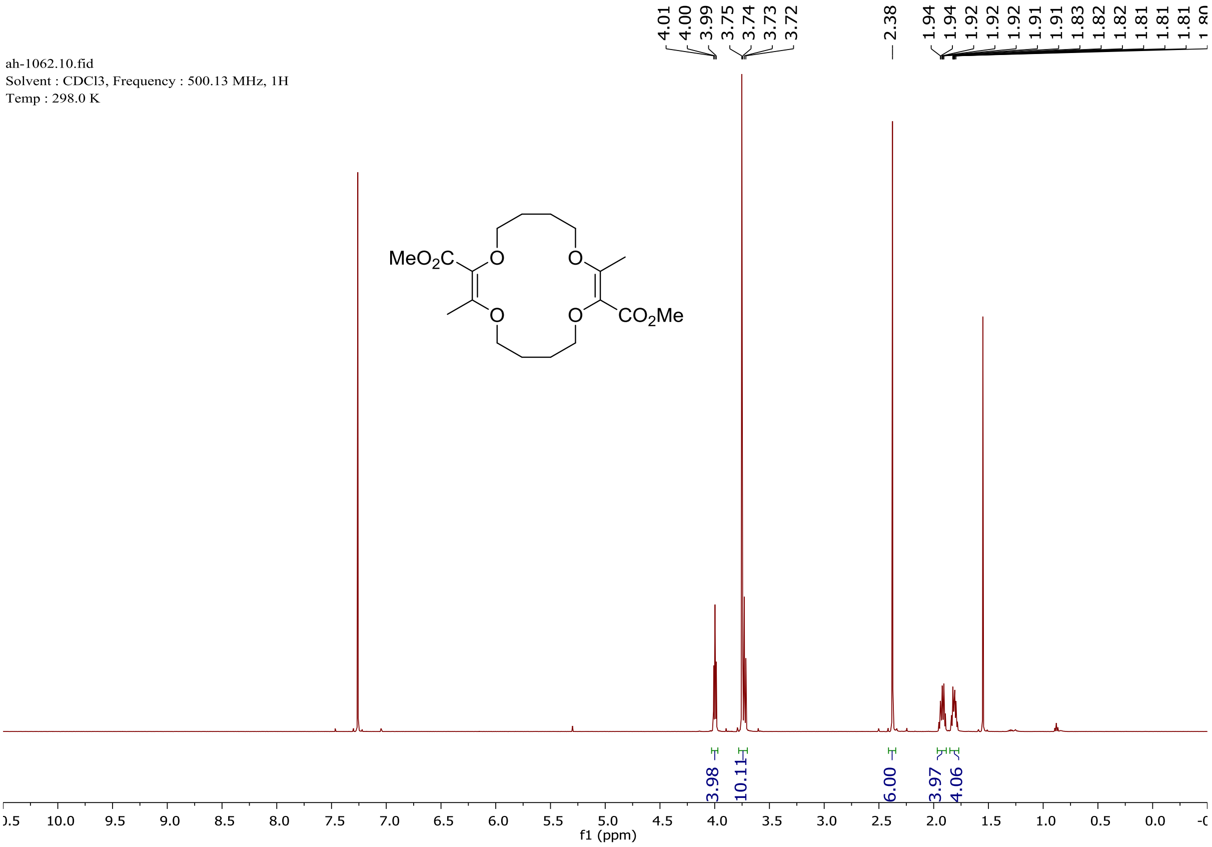

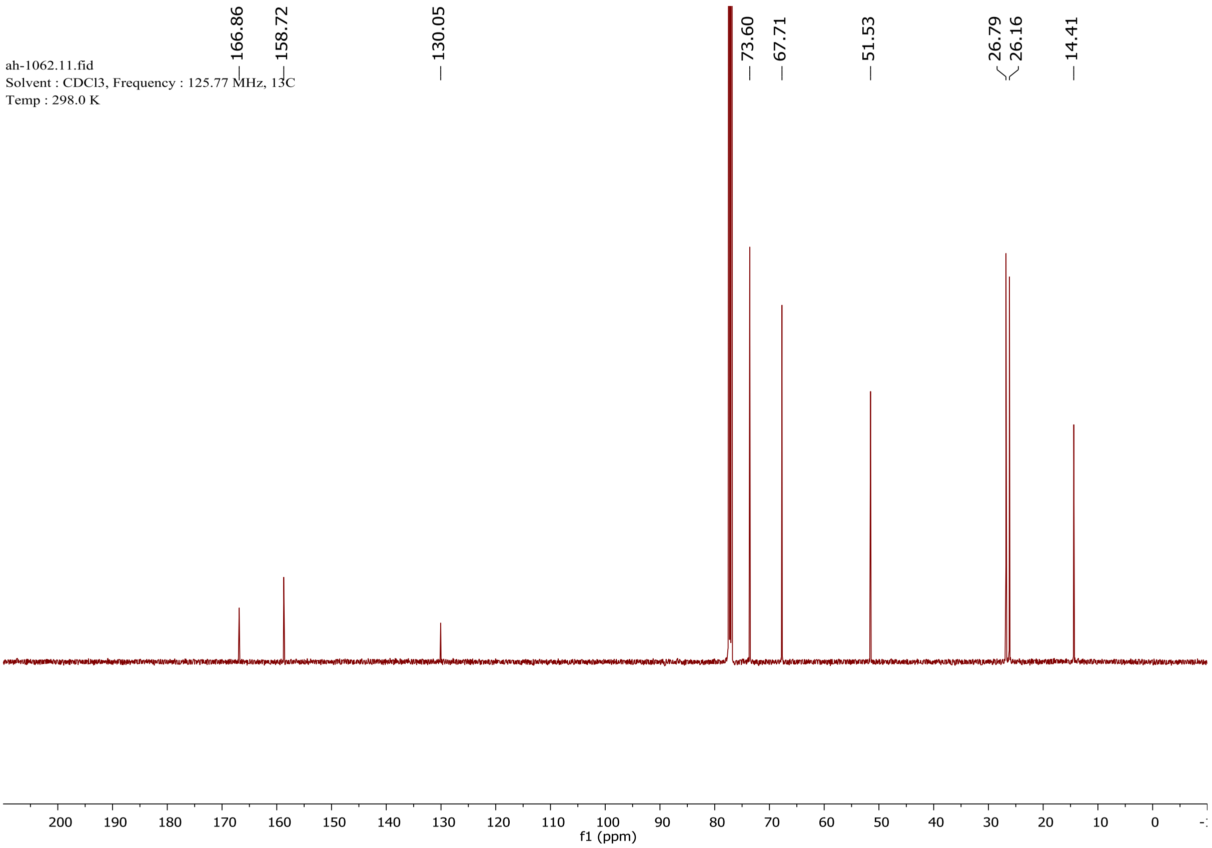

2.6.2. Polyether macrocycles **18C4**

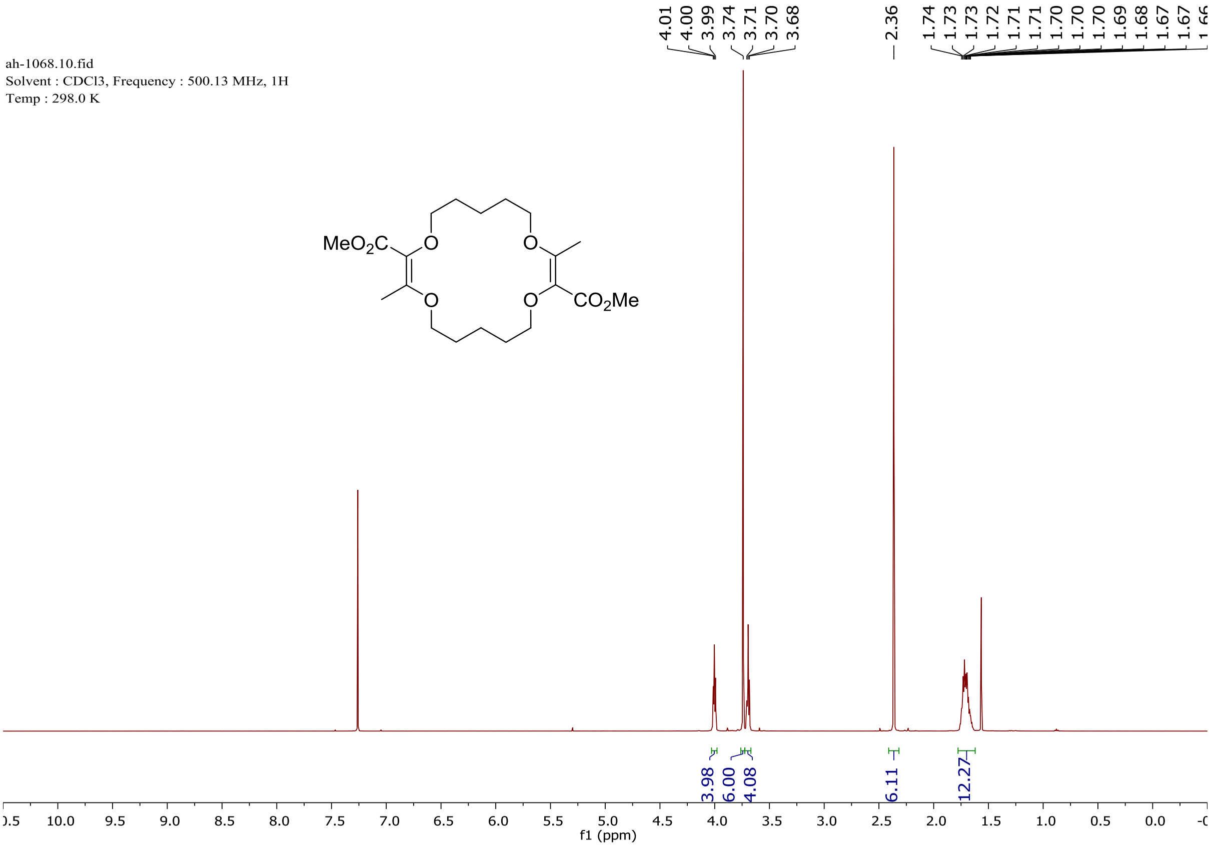

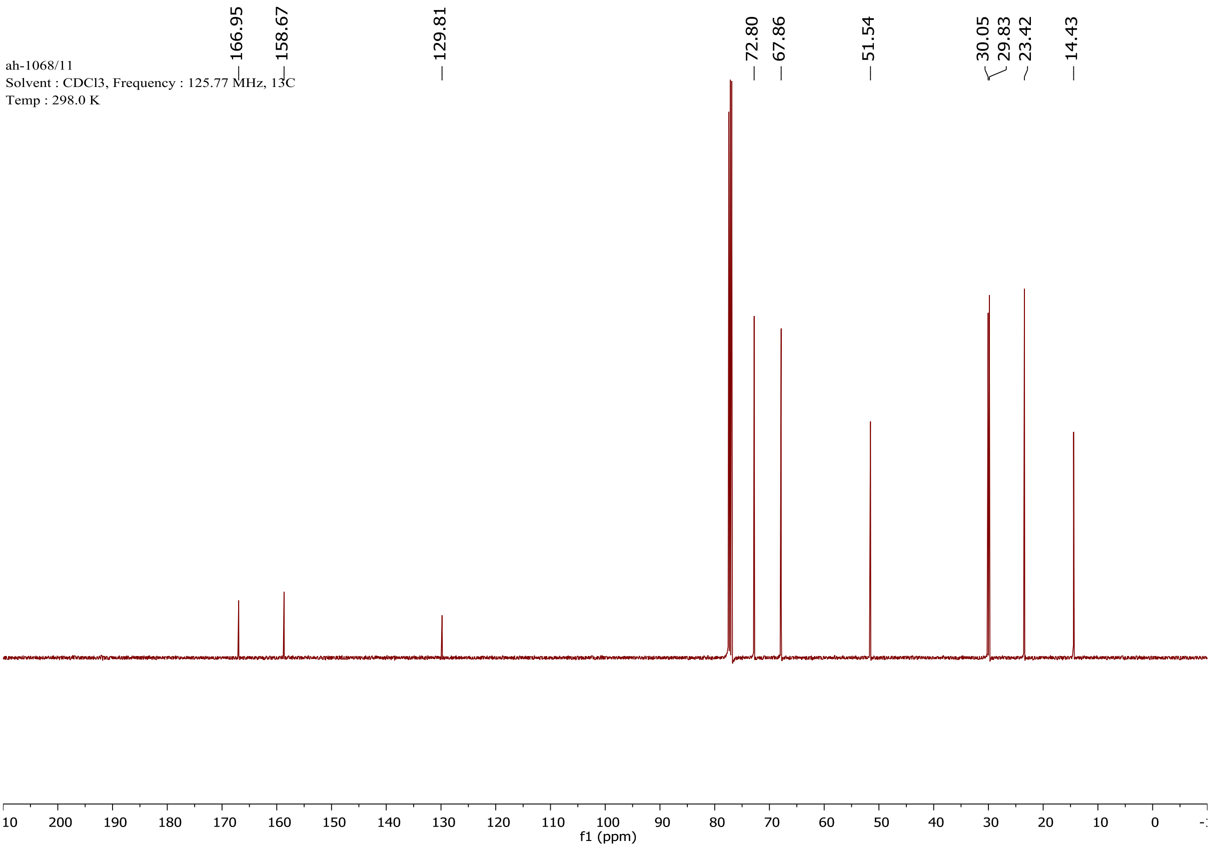

2.7. Functionalized polyether macrocycles NMR spectra

2.7.1. Perylene-18C6

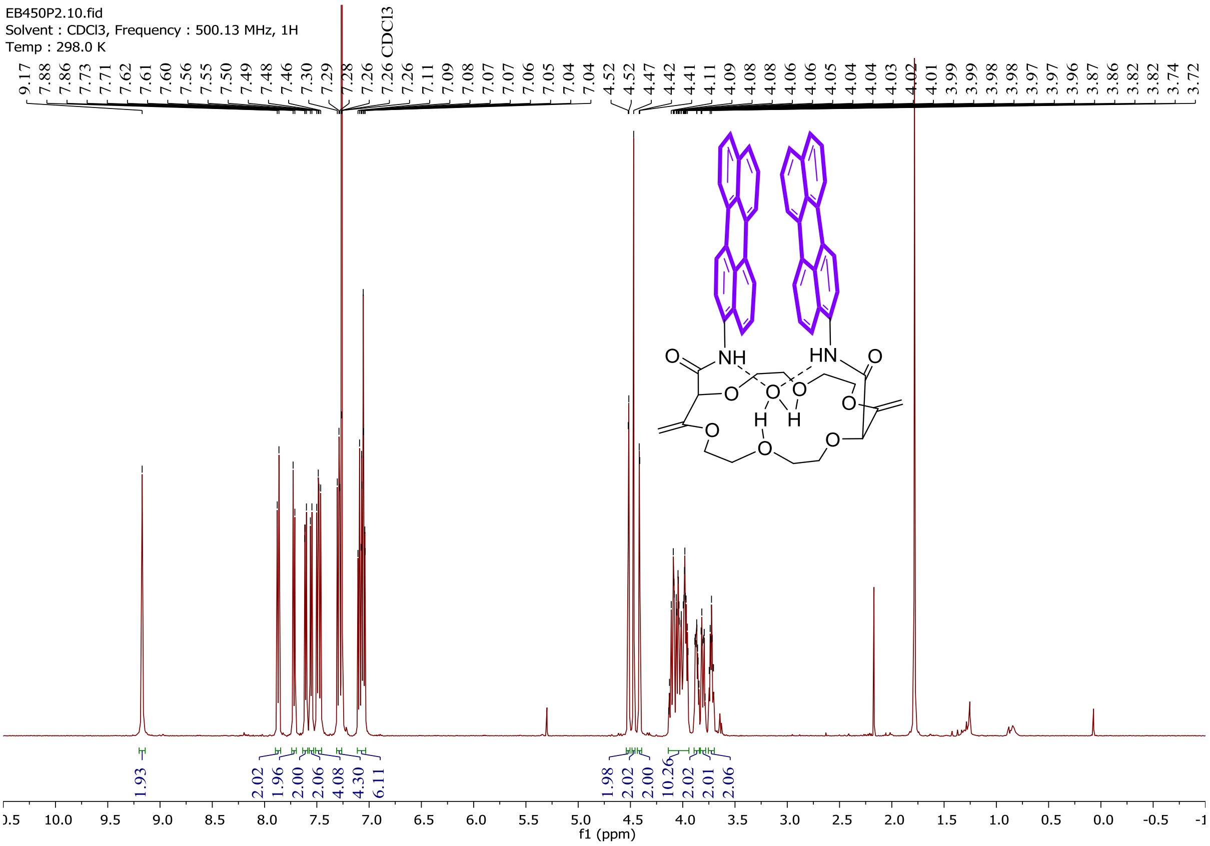

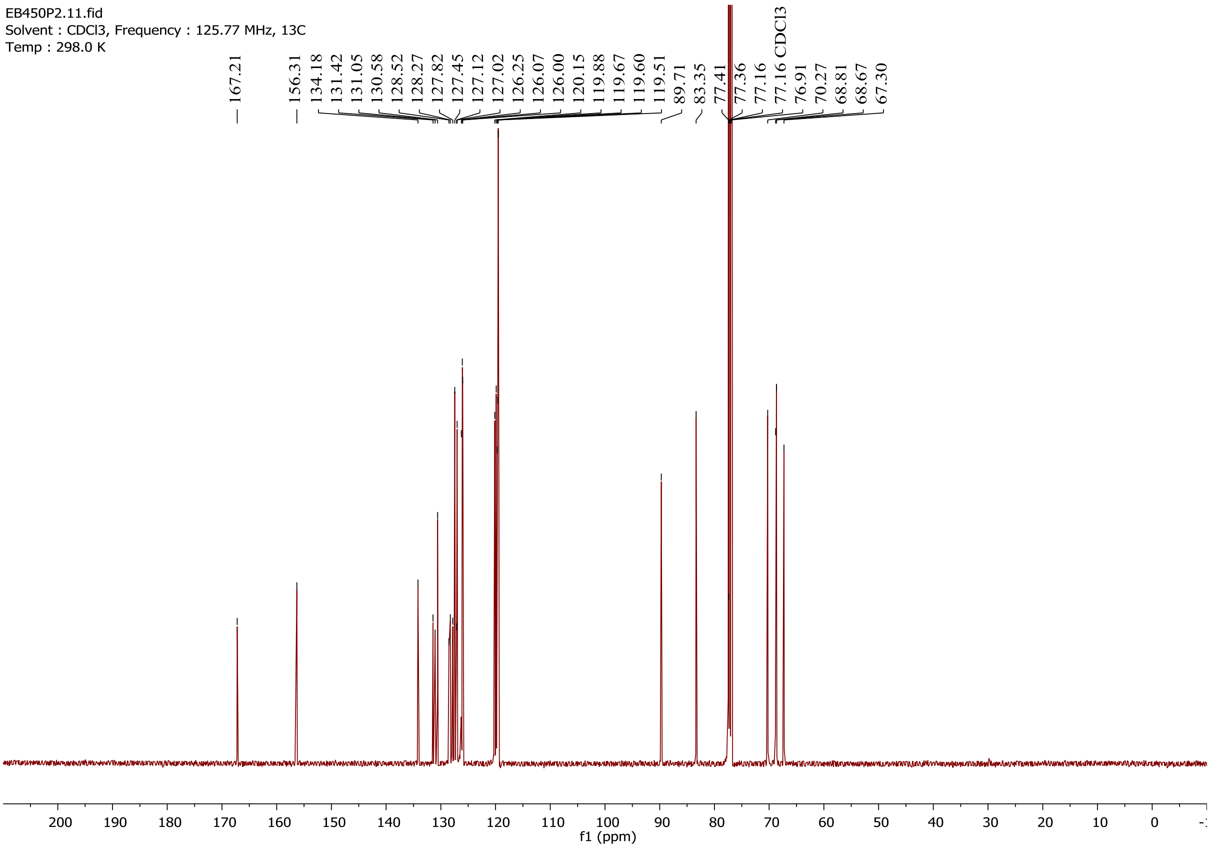

2.7.2. Perylene-18C4

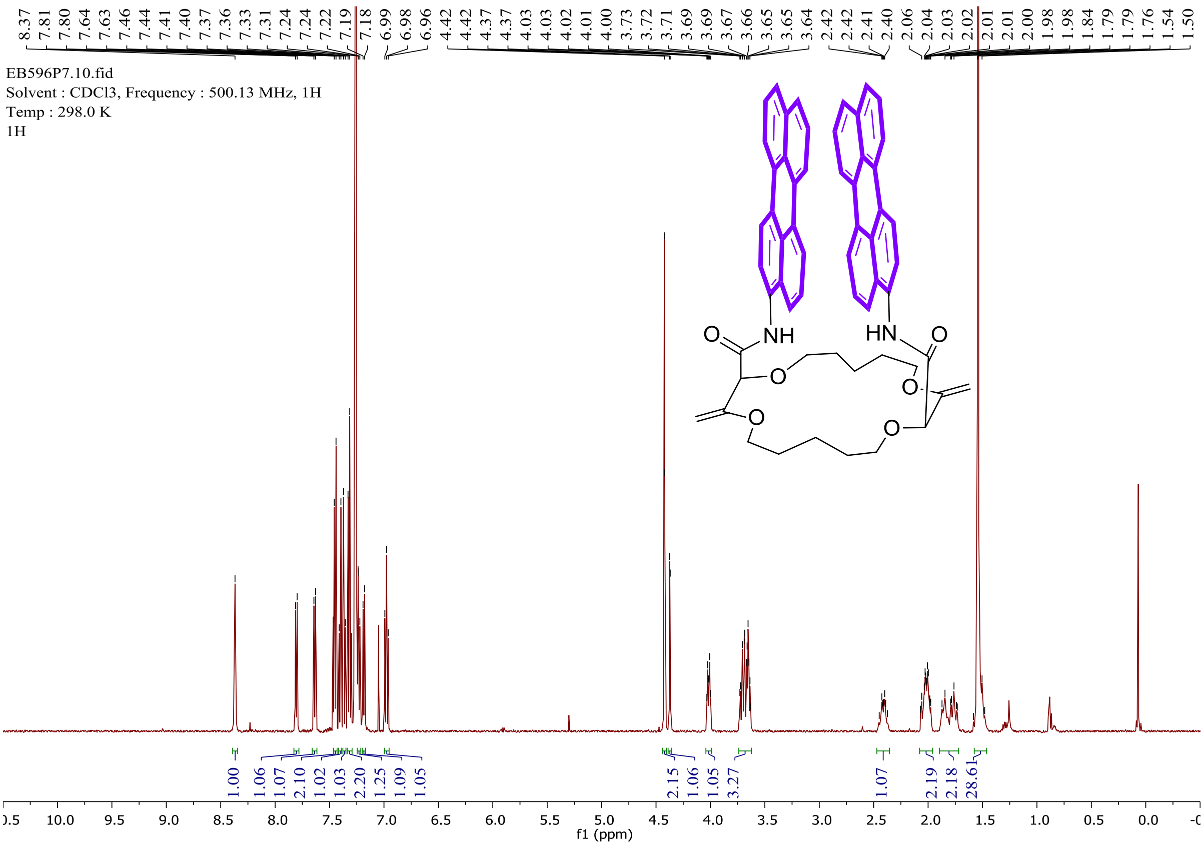

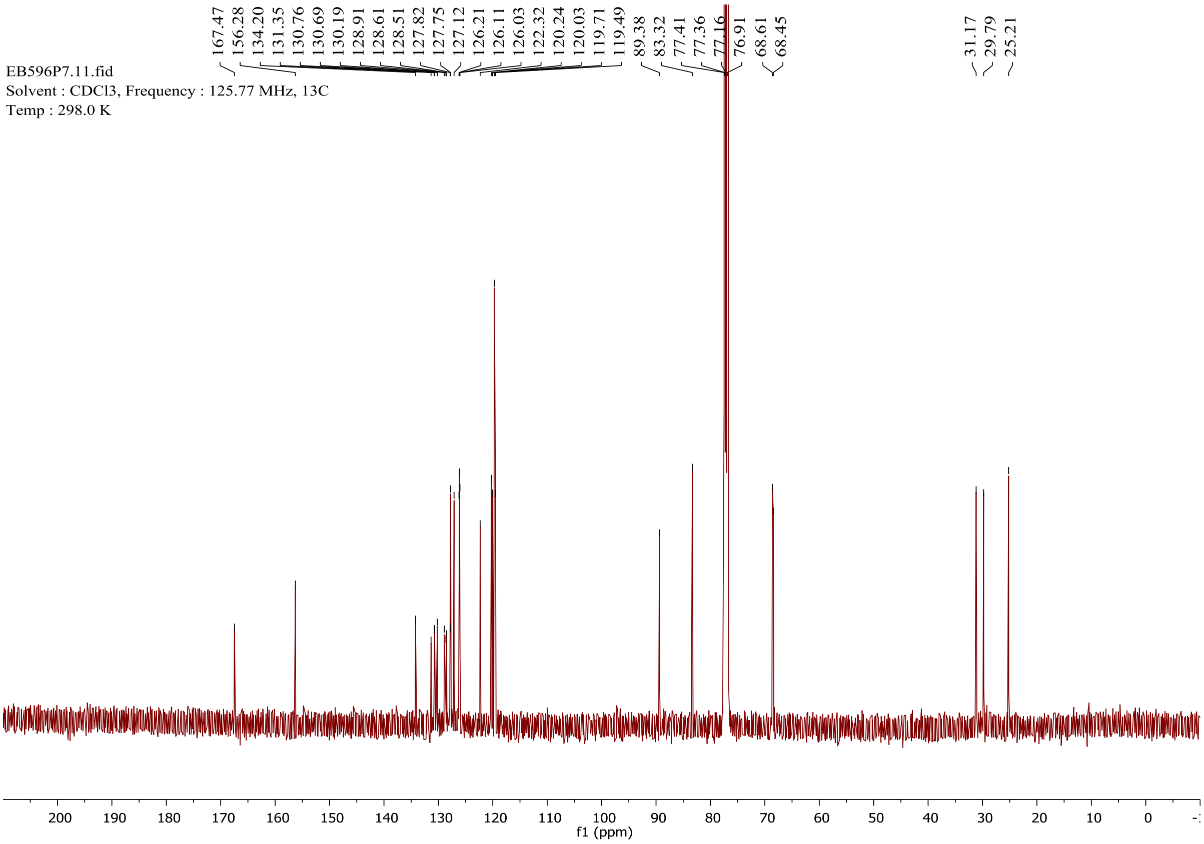

2.7.3. Perylene-16C4

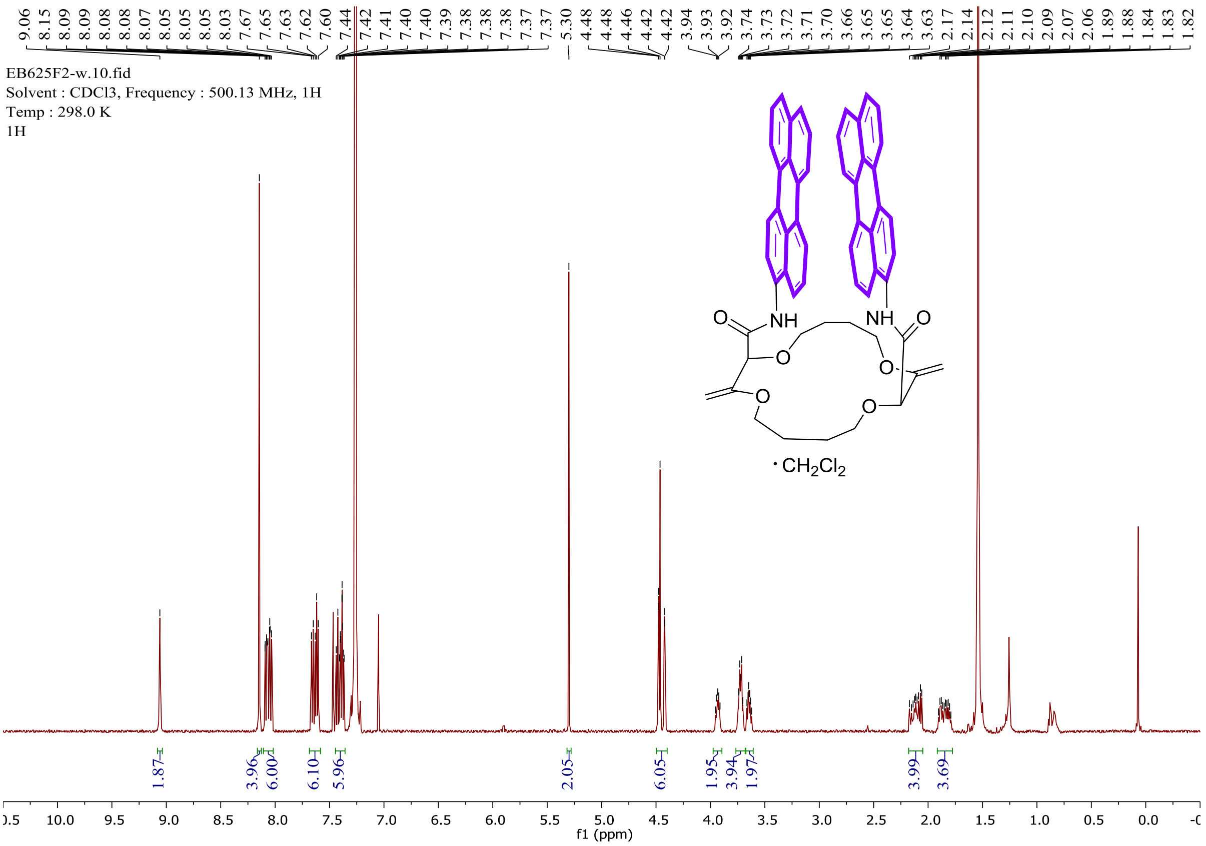

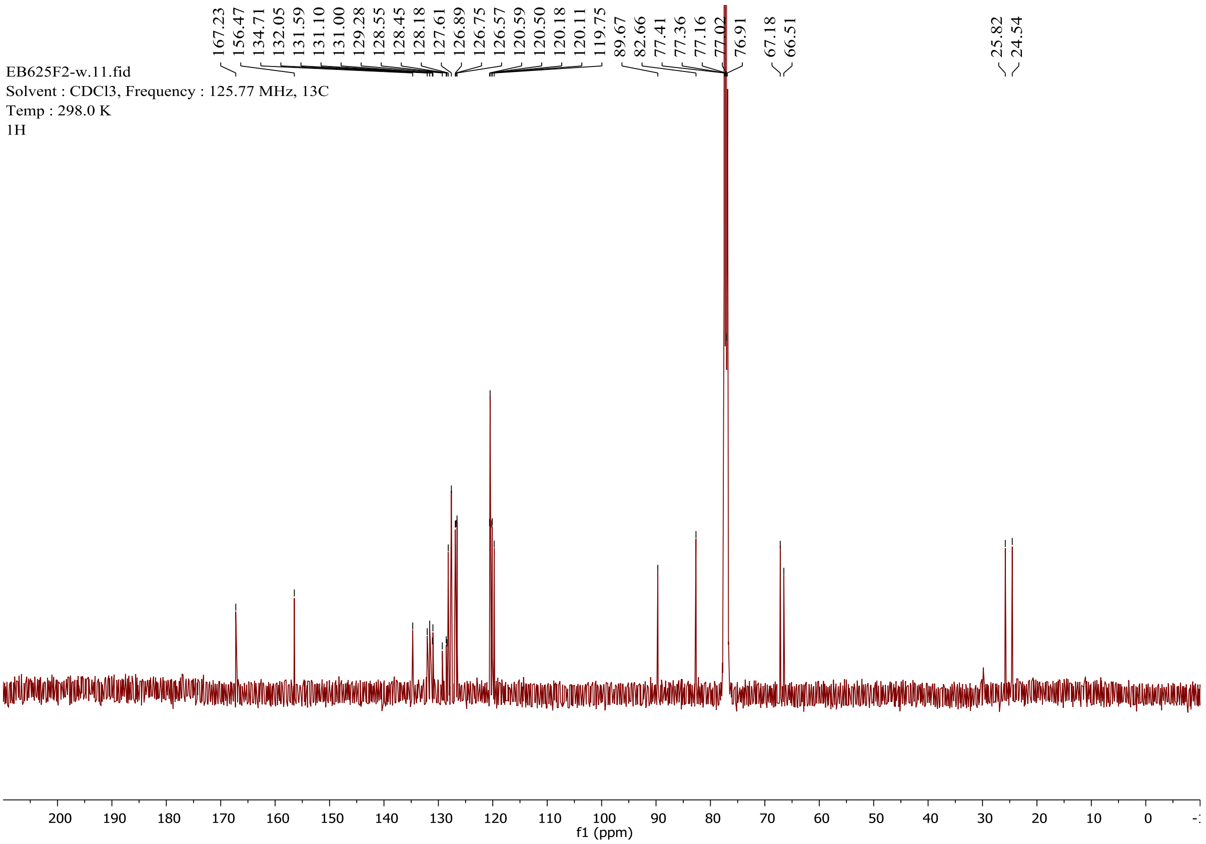

2.7.4. Fluorene-18C6

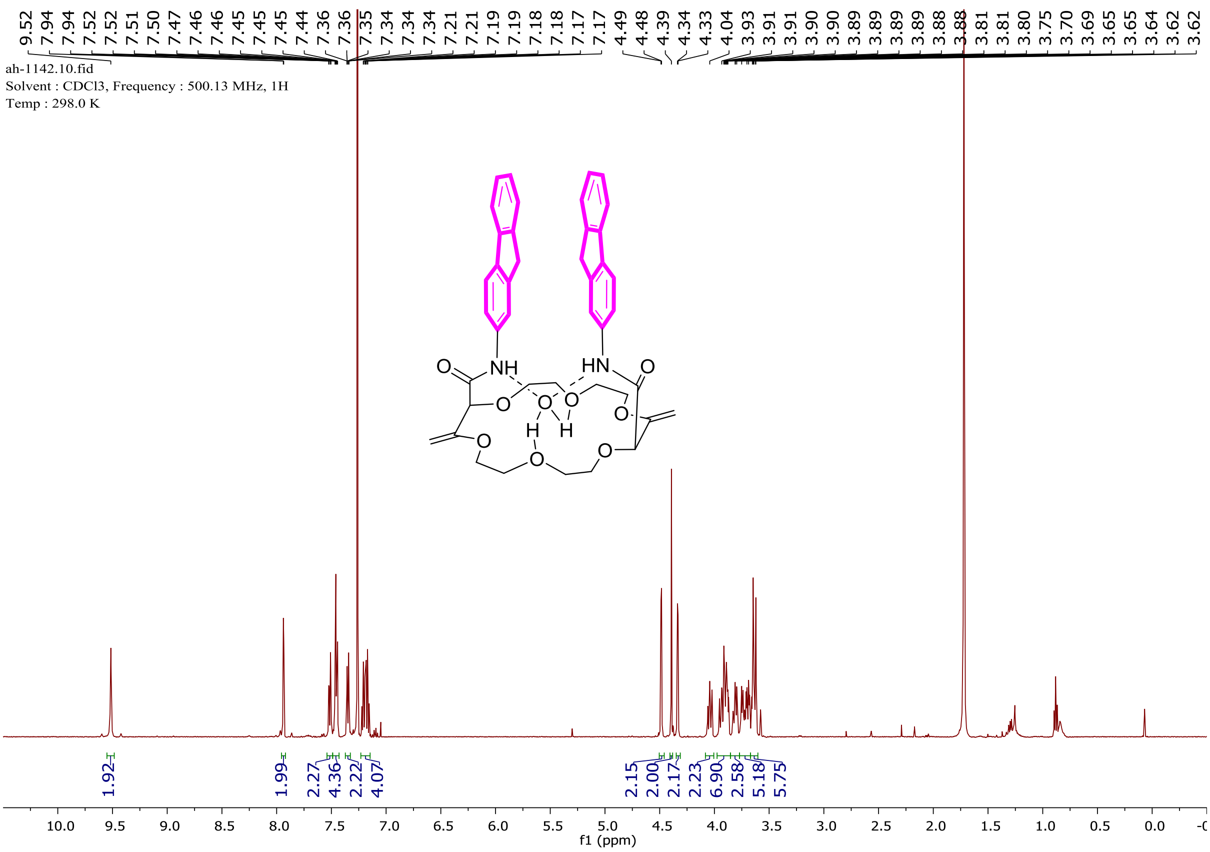

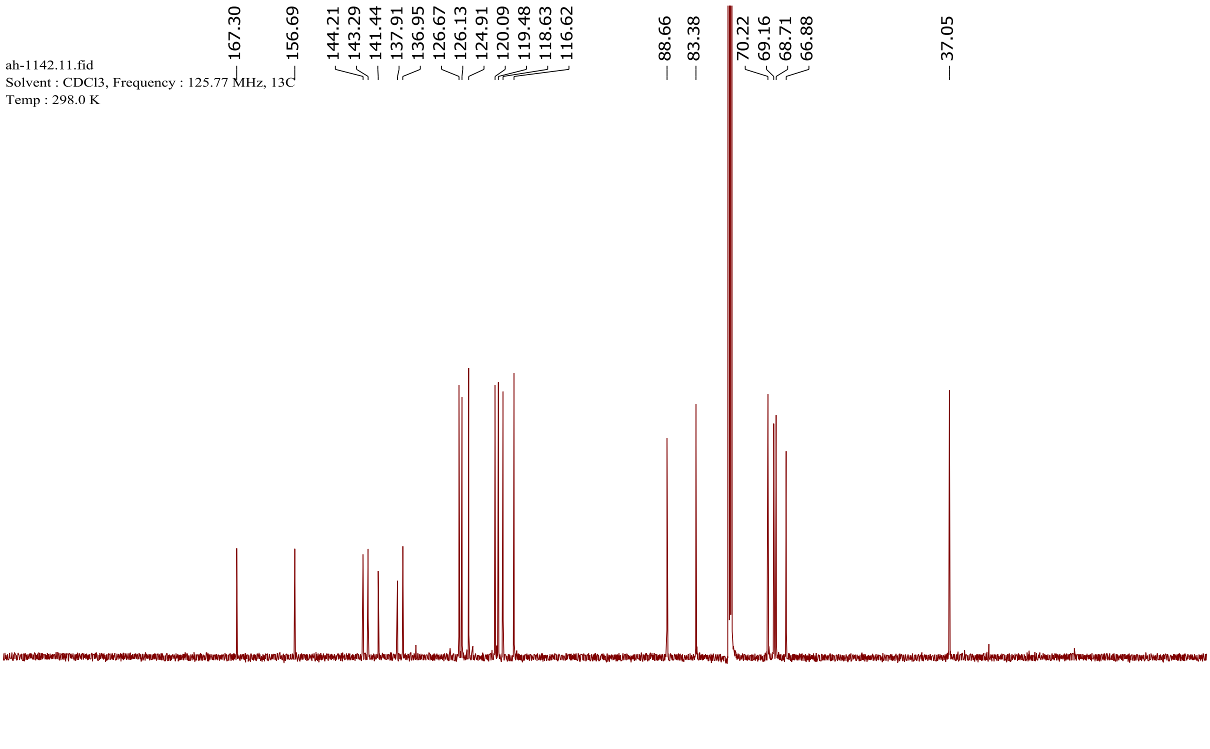

2.7.5. Fluorene-18C4

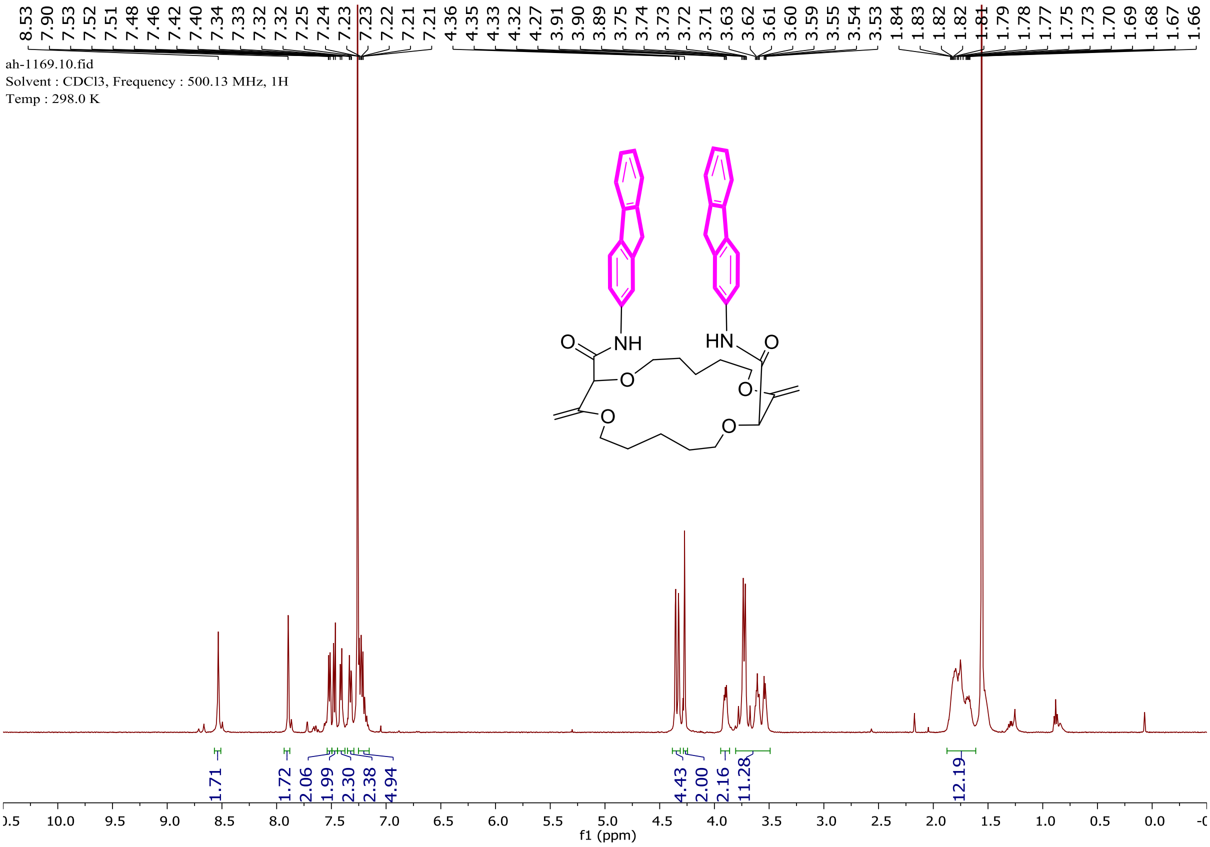

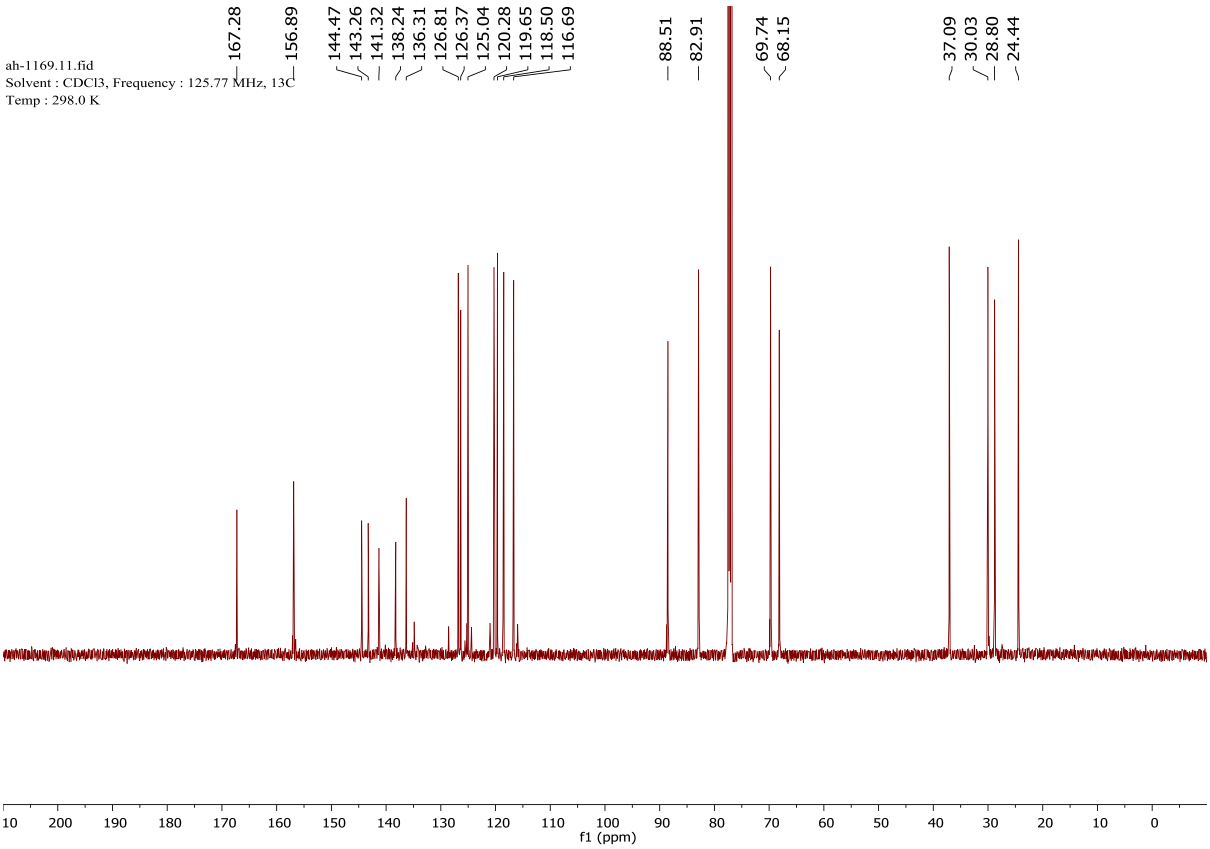

2.7.6. Fluorene-16C4

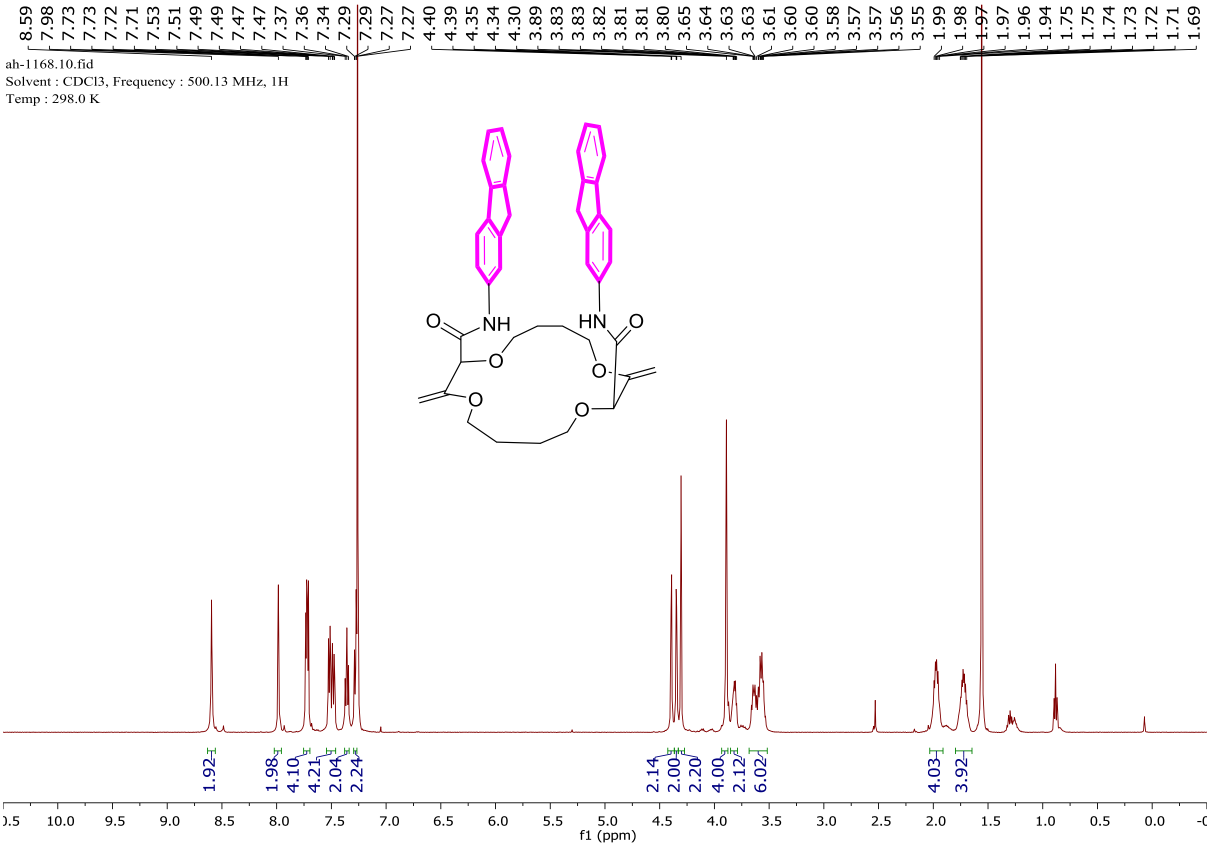

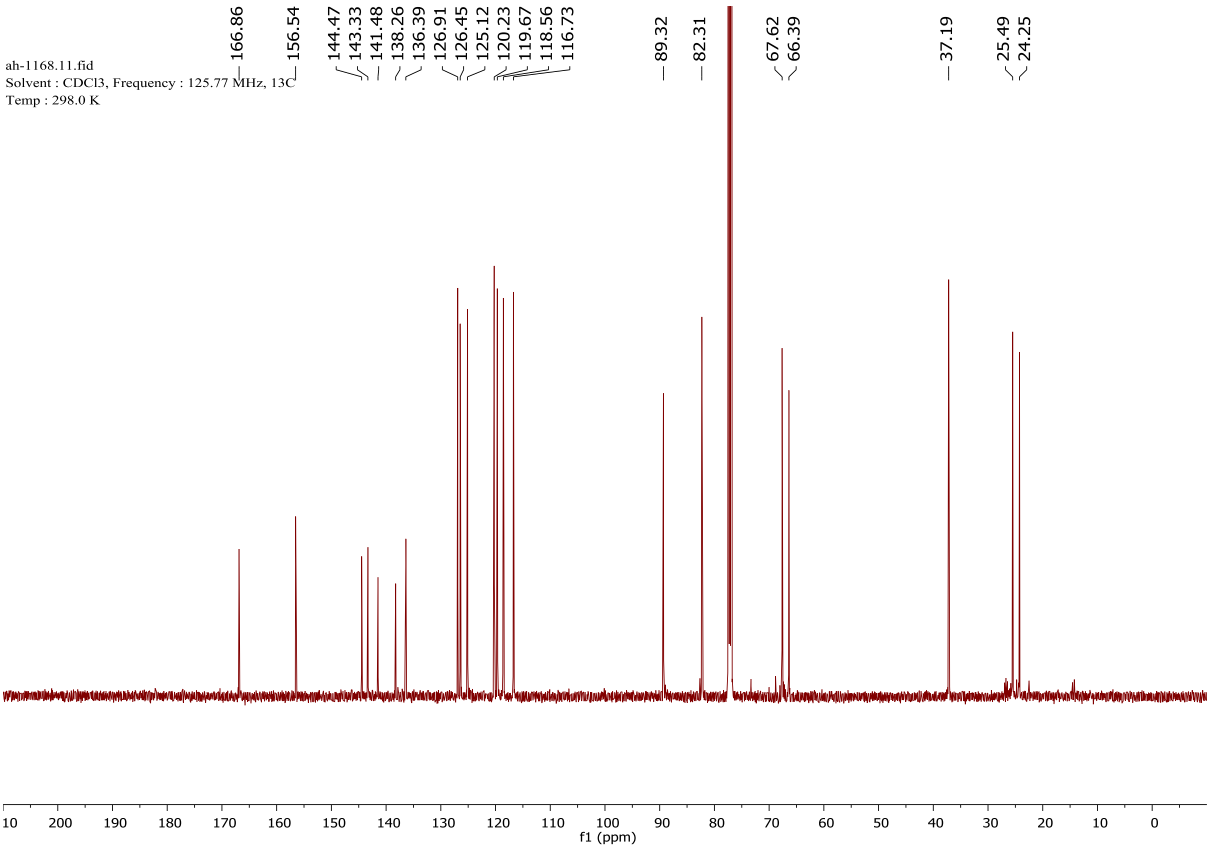

2.7.7. NMI-18C6

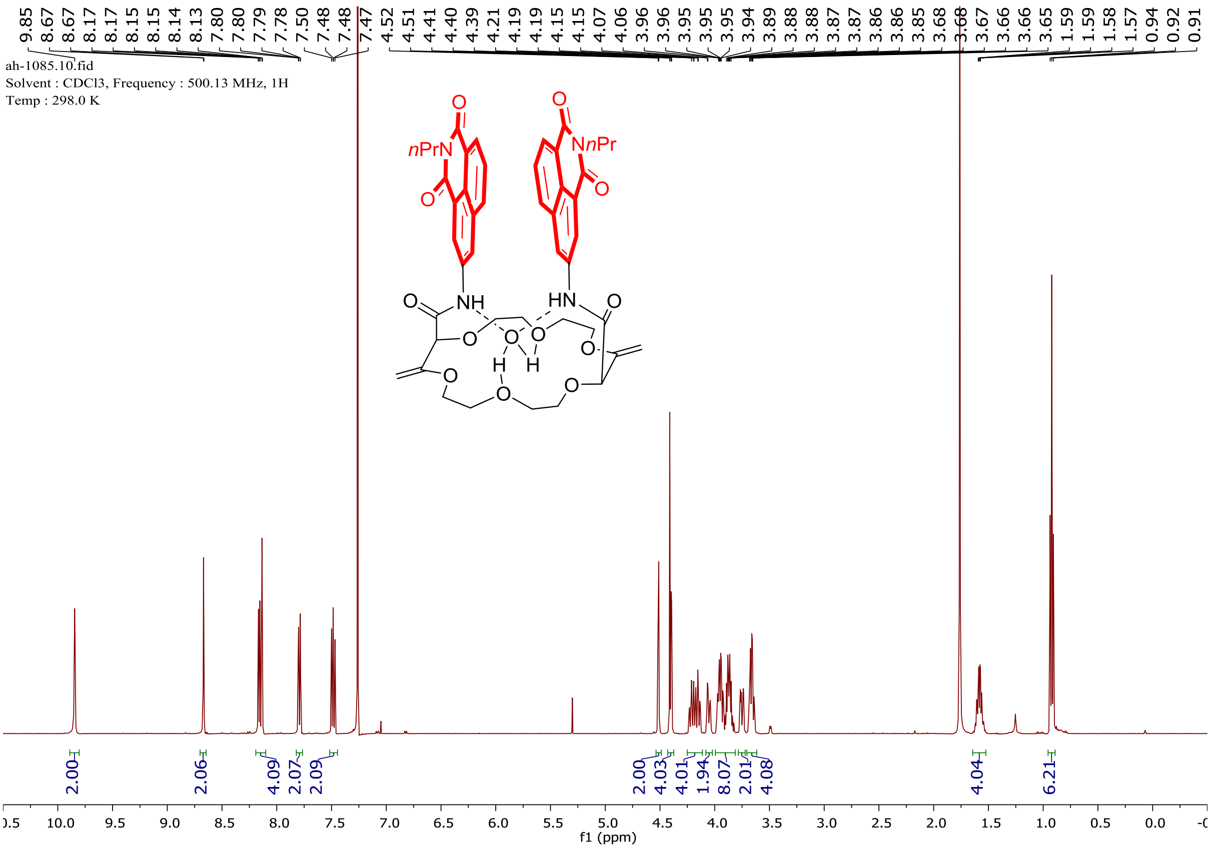
9.85 8.67 8.67 8.17 8.17 8.15 8.15 8.14 8.13 7.80 7.79 7.78 7.50 7.48 7.48 7.47 4.52 4.51 4.41 4.40 4.39 4.21 4.19 4.19 4.15 4.15 4.07 4.06 3.96 3.96 3.95 3.95 3.94 3.89 3.88 3.87 3.87 3.86 3.86 3.85 3.68 3.68 3.67 3.66 3.66 3.65 1.59 1.59 1.58 1.57 0.94 0.92 0.91
ah-1085.10.fid
Solvent : CDCl3, Frequency : 500.13 MHz, 1H
Temp : 298.0 K
2.00
2.06
4.09
2.07
2.09
2.00
4.03
4.01
1.94
8.07
2.01
4.08
4.04
6.21
f1 (ppm)

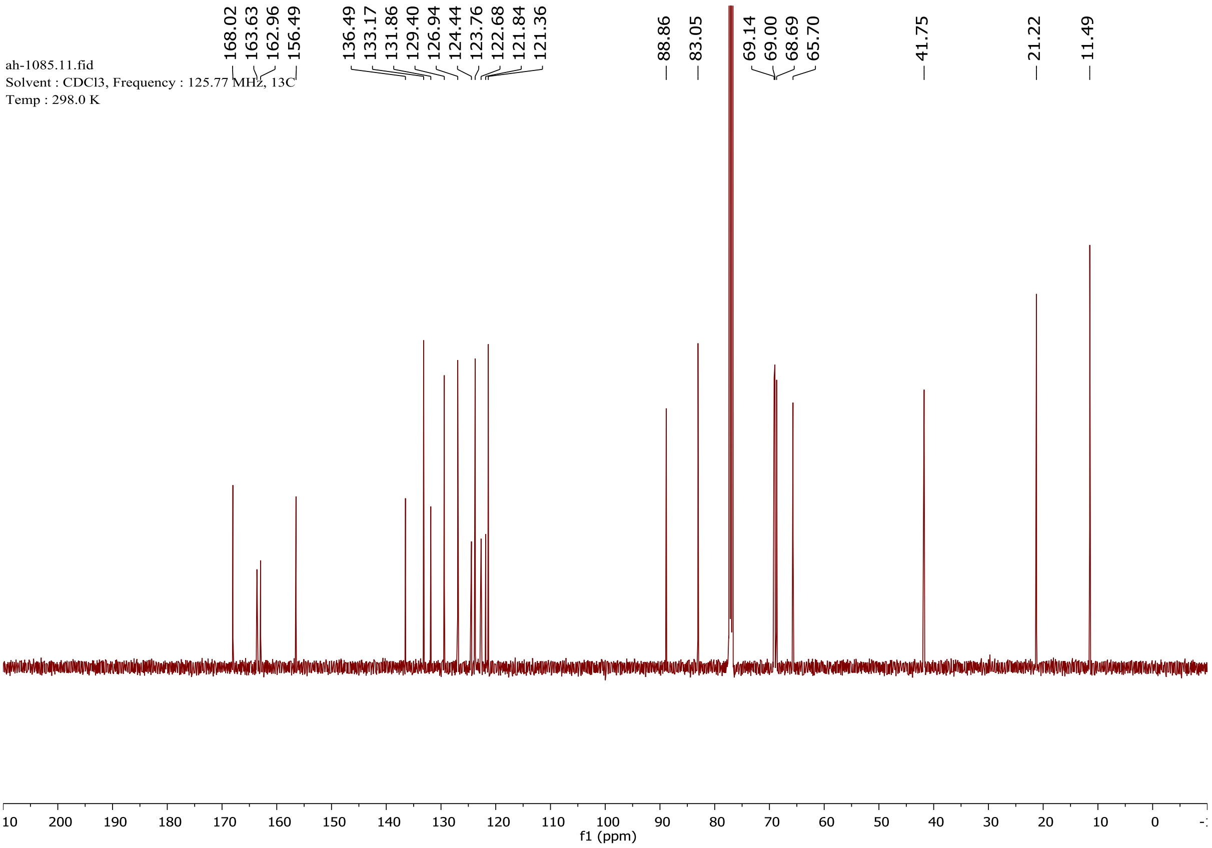
168.02 163.63 162.96 156.49 136.49 133.17 131.86 129.40 126.94 124.44 123.76 122.68 121.84 121.36 88.86 83.05 69.14 69.00 68.69 65.70 41.75 21.22 11.49
ah-1085.11.fid
Solvent : CDCl3, Frequency : 125.77 MHz, 13C
Temp : 298.0 K
f1 (ppm)

3. CSP-HPLC separation

3.1. General remarks

Compounds were resolved by chiral stationary phase HPLC using a semi-preparative CHIRALPAK® IG column. It is worth mentioning that it is necessary to remove traces of $Et_2NH.HCl$ present in the separated compounds. The residue was thus dissolved in CH_2Cl_2, the organic phase was washed three times with H_2O, dried over anhydrous Na_2SO_4, filtered and concentrated under vacuum to afford the pure products.

3.2. Summary of separation data

The separation data are summarized in the following table:

Table S1. Enantiomer separations by CSP HPLC: mobile phase, retention times and α

Entry	Compound	Mobile phase[a]	retention time (min)		α
			1st eluted	2nd eluted	
1	**Pyrene-18C6**	7:3	13.57	21.84	1.81
2	**Pyrene-18C4**	7:3	12.06	26.18	2.53
3	**Pyrene-16C4**	8:2[b]	9.40	34.93	6.22
4	**Perylene-18C6**	9:1[b]	13.36	51.37	5.32
5	**Perylene-18C4**	8:2[b]	6.07	12.95	3.41
6	**Perylene-16C4**	8:2[b]	9.97	55.46	7.71
7	**Fluorene-18C6**	7:3	11.12	24.17	2.69
8	**Fluorene-18C4**	7:3[b]	7.55	16.07	3.80
9	**Fluorene-16C4**	7:3[b]	8.92	26.56	4.99
10	**NMI-18C6**	9:1	8.64	14.12	2.05

[a] CH_2Cl_2+0.1% Et_2NH/CH_3CN+0.1% Et_2NH, flow = 2 mL/min, 20 °C; [b] flow = 3 mL/min.

α is calculated using the following formula: $\alpha = \frac{t_{r2}-t_0}{t_{r1}-t_0}$

where t_{r1}/t_{r2} are the retention time of the first and the second eluted enantiomer and t_0 is the dead time.

The optical rotation of the enantiopure fractions are collected in the following table:

Table S2. Optical rotation of enantiopure compounds

Entry	Compound	First eluted enantiomer		Second eluted enantiomer	
		concentration (g/mL)	$[\alpha]_D^{20}$	concentration (g/mL)	$[\alpha]_D^{20}$
1	**Pyrene-18C6**[a]	$8.74 \cdot 10^{-6}$	-400	$6.03 \cdot 10^{-6}$	+365
2	**Pyrene-18C4**[b]	$1.6 \cdot 10^{-4}$	-275	$1.2 \cdot 10^{-4}$	+223
3	**Pyrene-16C4**[b]	$1.0 \cdot 10^{-4}$	-144	$1.3 \cdot 10^{-4}$	+112
4	**Perylene-18C6**[b]	$2.5 \cdot 10^{-4}$	-184	$2.0 \cdot 10^{-4}$	+163
5	**Perylene-18C4**[c]	$4.50 \cdot 10^{-5}$	+133	$3.47 \cdot 10^{-5}$	-98
6	**Perylene-16C4**[c]	$4.06 \cdot 10^{-6}$	+271	$6.63 \cdot 10^{-6}$	-316
7	**Fluorene-18C6**[b]	$7.0 \cdot 10^{-5}$	-37	$7.0 \cdot 10^{-5}$	+31
8	**Fluorene-18C4**[b]	$3.5 \cdot 10^{-4}$	-23	$2.5 \cdot 10^{-4}$	+20
9	**Fluorene-16C4**[b]	$1.2 \cdot 10^{-4}$	+17	$1.3 \cdot 10^{-4}$	-15
10	**NMI-18C6**[a]	$1.40 \cdot 10^{-4}$	-64	$1.02\ 10^{-4}$	+43

[a] in acetonitrile; [b] in $CHCl_3$; [c] in CH_2Cl_2.

3.3. HPLC traces

For each compound, the data are reported as follows: separation conditions, analytical HPLC trace of the racemate, analytical HPLC trace of the first eluted enantiomer, analytical HPLC trace of the second eluted enantiomer and semi-preparative HPLC trace.

3.3.1. Pyrene-18C6

CSP-HPLC: semi-preparative IG, CH_2Cl_2 (+0.1% Et_2NH)/CH_3CN (+0.1% Et_2NH) = 70:30, 2 mL/min, 20 °C, sample: 23 mg/1.5 mL of CH_2Cl_2, injection 500 µL.

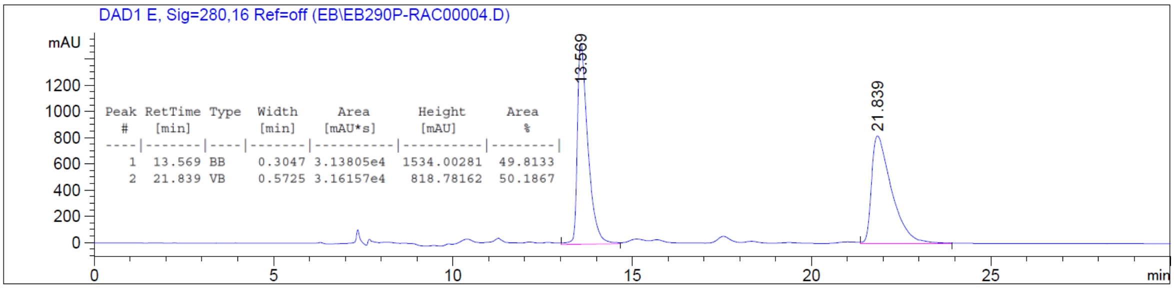

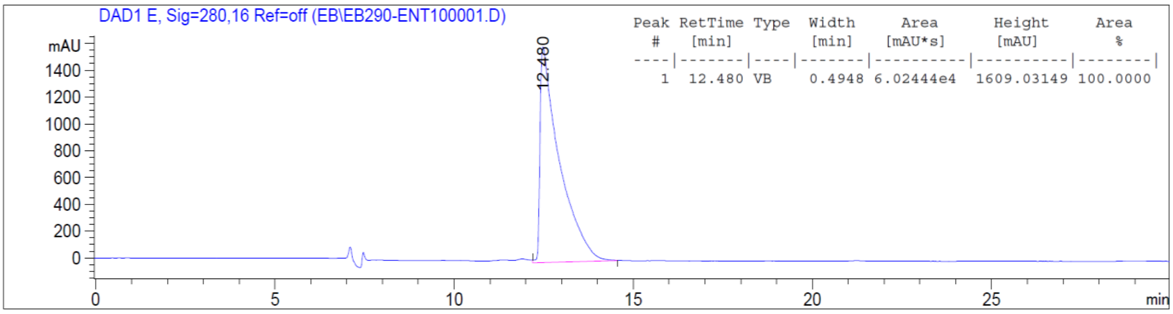

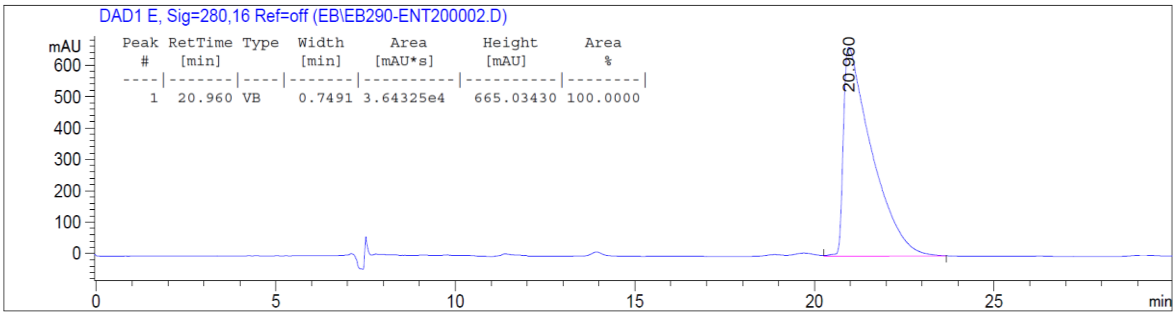

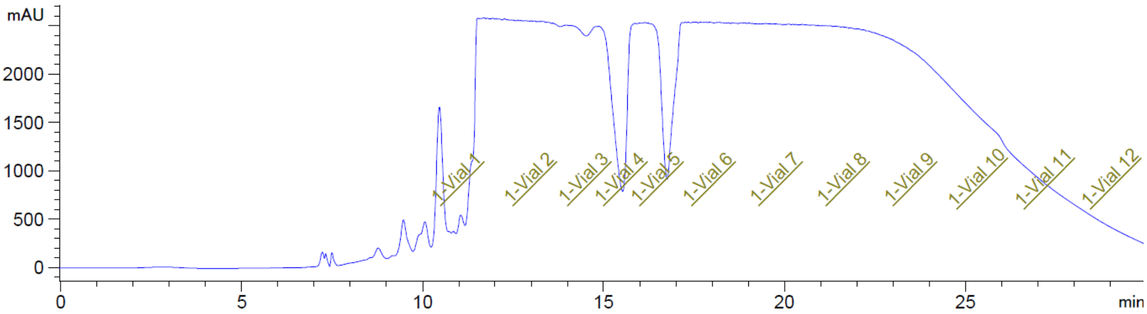

1st enantiomer: vials 1 to 4 were collected
2nd enantiomer: vials 5 to 12 were collected

3.3.2. Pyrene-18C4

CSP-HPLC: semi-preparative IG, CH_2Cl_2 (+0.1% Et_2NH)/CH_3CN (+0.1% Et_2NH) = 70:30, 2 mL/min, 20 °C, sample: 25 mg/1.5 mL of CH_2Cl_2, injection 500 µL.

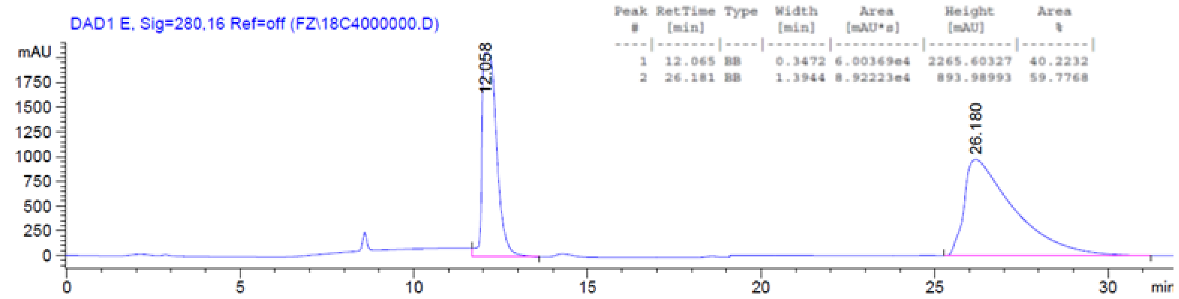

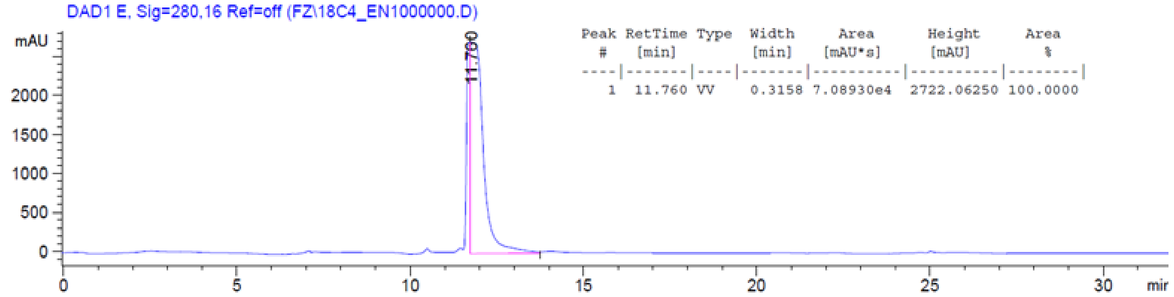

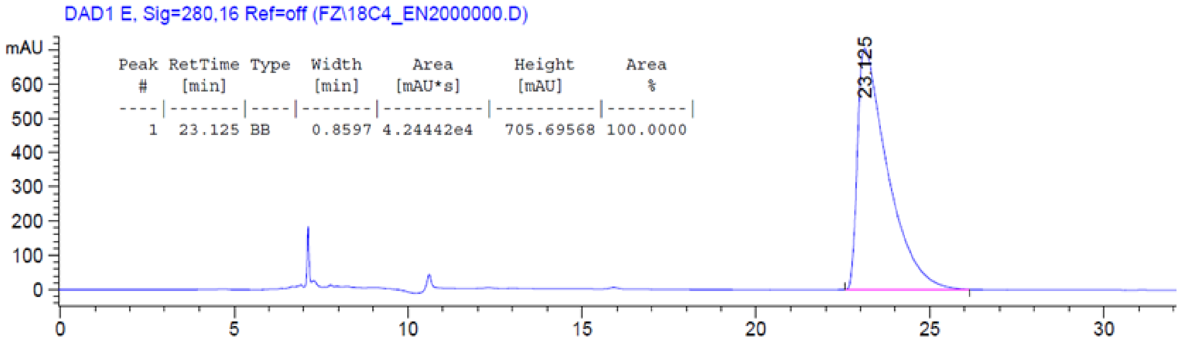

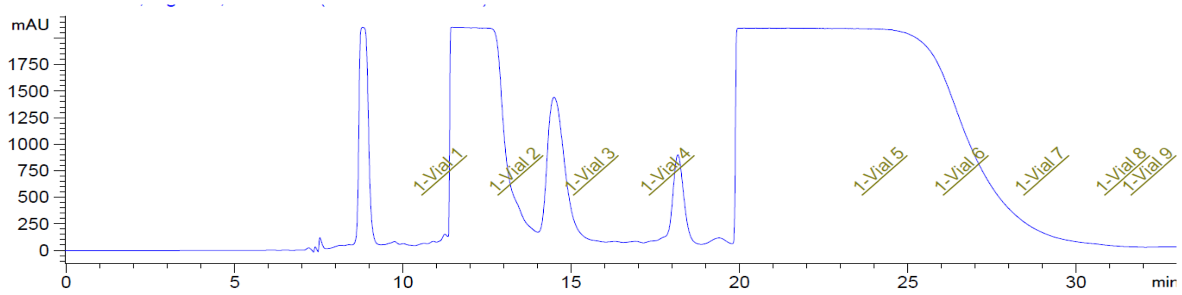

1st enantiomer: vials 1 and 2 were collected
2nd enantiomer: vials 4 to 7 were collected

3.3.3. Pyrene-16C4

CSP-HPLC: semi-preparative IG, CH_2Cl_2 (+0.1% Et_2NH)/CH_3CN (+0.1% Et_2NH) = 80:20, 3 mL/min, 20 °C, sample: 16 mg/1.0 mL of CH_2Cl_2, injection 500 µL.

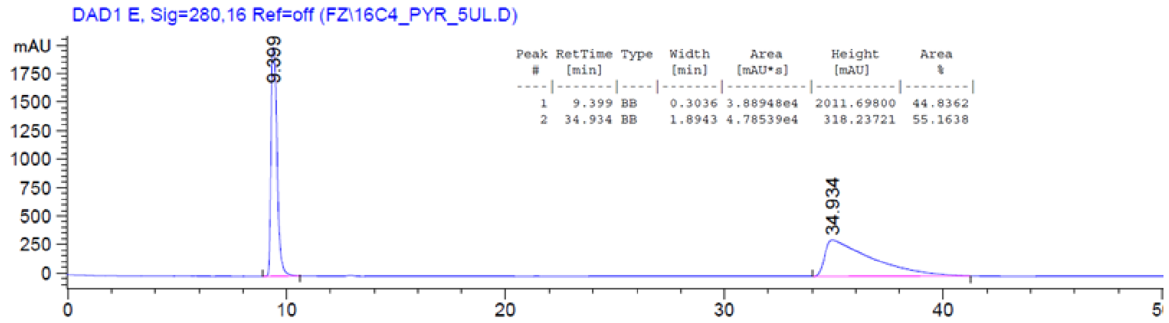

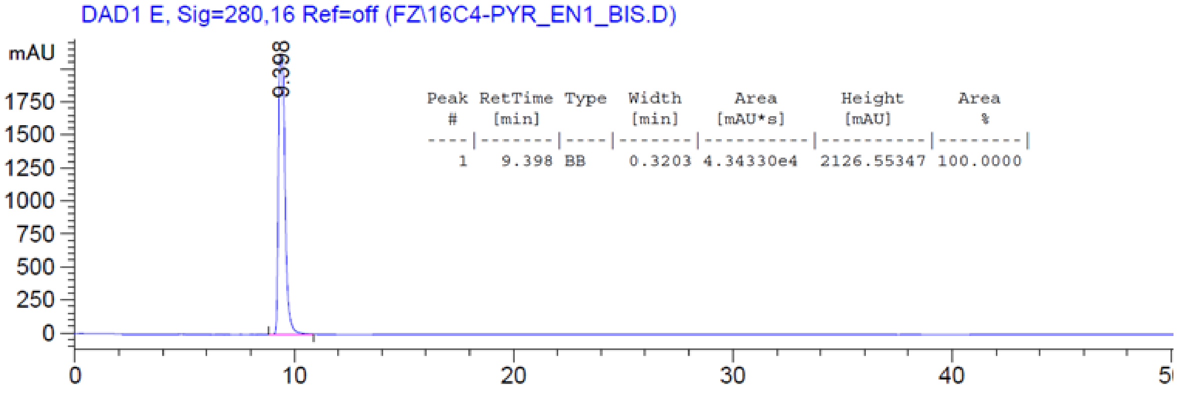

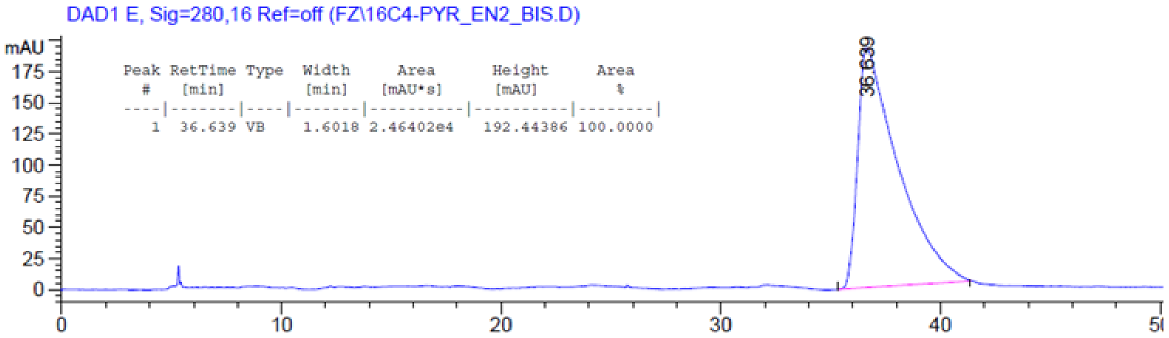

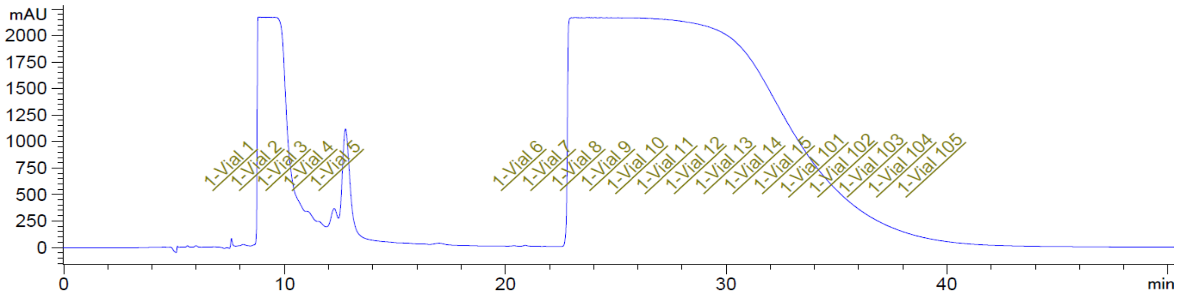

1st enantiomer: vials 1 to 4 were collected
2nd enantiomer: vials 7 to 105 were collected

3.3.4. Perylene 18C6

CSP-HPLC: semi-preparative IG, CH_2Cl_2 (+0.1% Et_2NH)/CH_3CN (+0.1% Et_2NH) = 90:10, 3 mL/min, 20 °C, sample: 13 mg/1.5 mL of CH_2Cl_2, injection 750 µL.

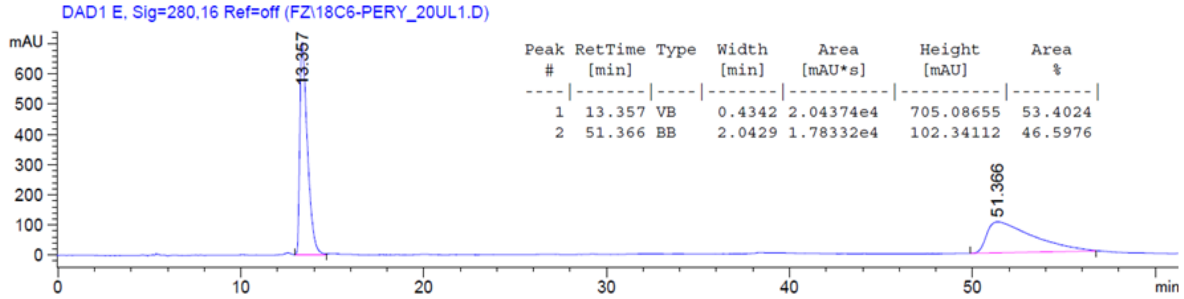

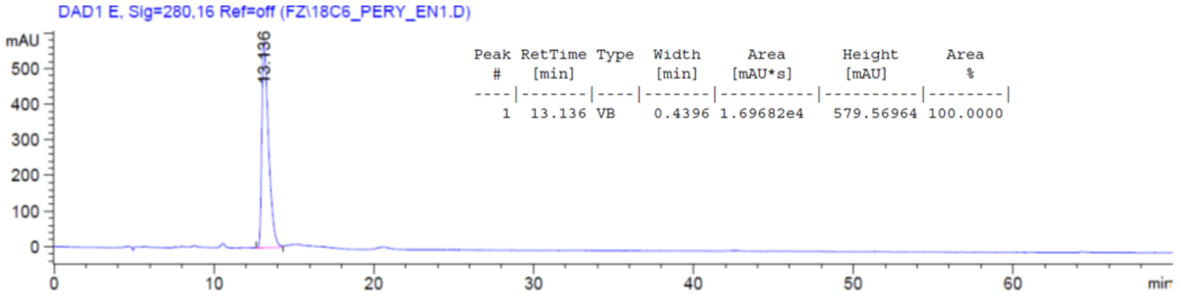

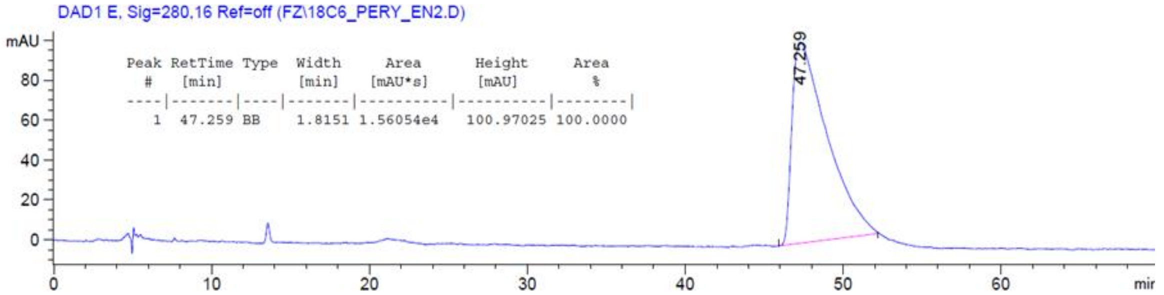

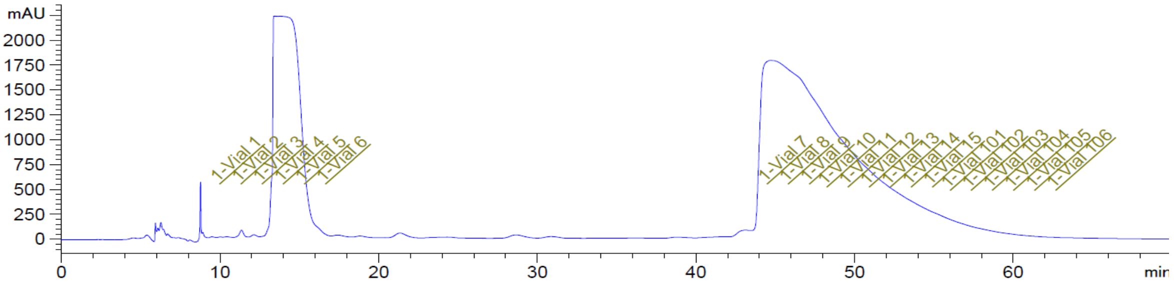

1st enantiomer: vials 2 to 5 were collected

2nd enantiomer: vials 7 to 105 were collected

3.3.5. Perylene-18C4

CSP-HPLC: semi-preparative IG, CH_2Cl_2 (+0.1% Et_2NH)/CH_3CN (+0.1% Et_2NH) = 80:20, 3 mL/min, 20 °C, sample: 5 mg/1.5 mL of CH_2Cl_2, injection 500 μL.

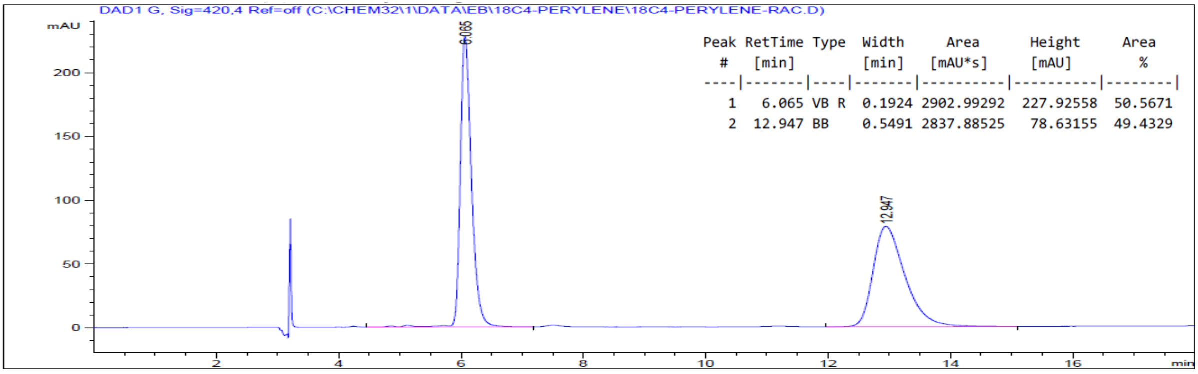

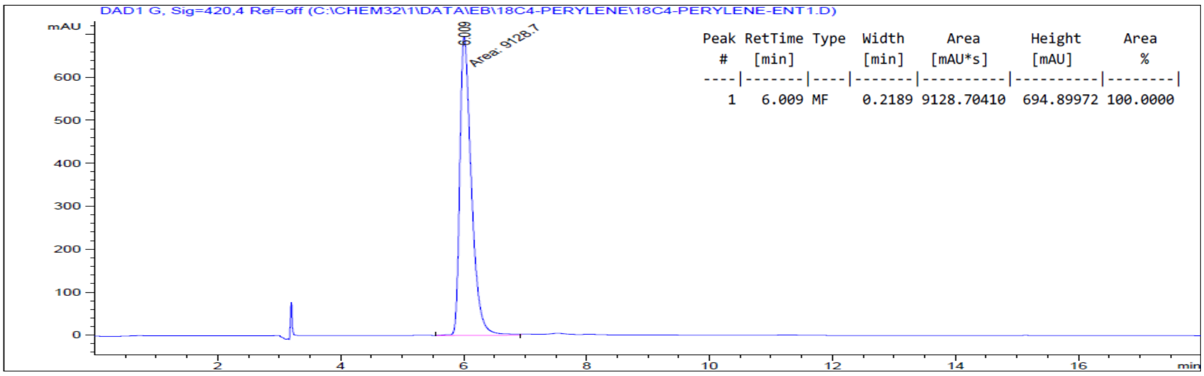

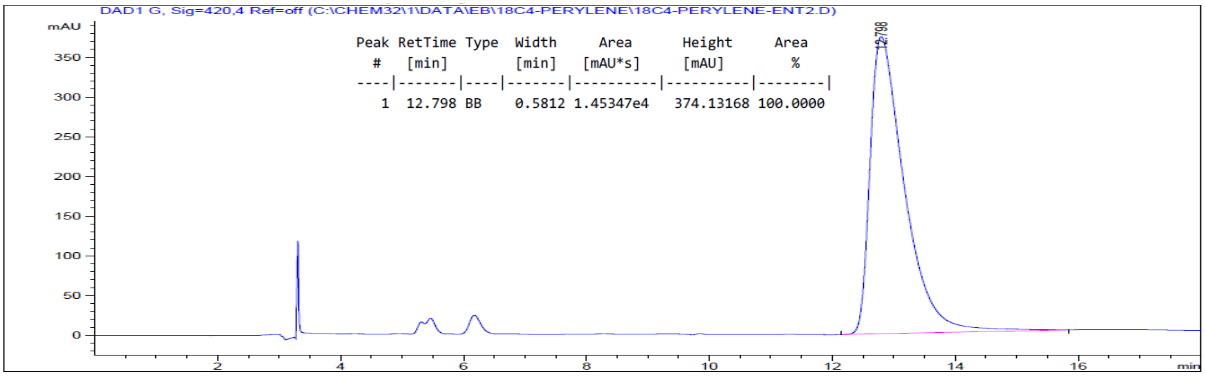

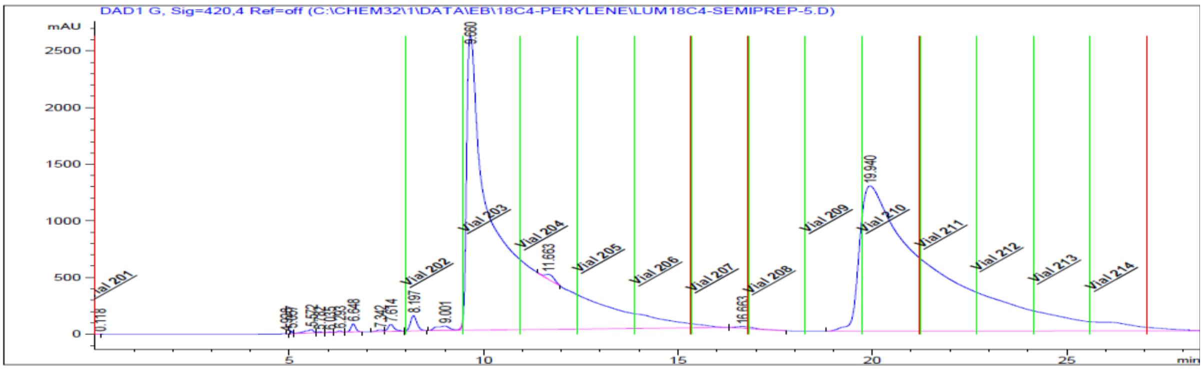

1st enantiomer: vials 3 to 5 were collected
2nd enantiomer: vials 9 to 12 were collected

3.3.6. Perylene-16C4

CSP-HPLC: semi-preparative IG, CH_2Cl_2 (+0.1% Et_2NH)/CH_3CN (+0.1% Et_2NH) = 80:20, 3 mL/min, 20 °C, sample: 5 mg/1.5 mL of CH_2Cl_2, injection 500 µL.

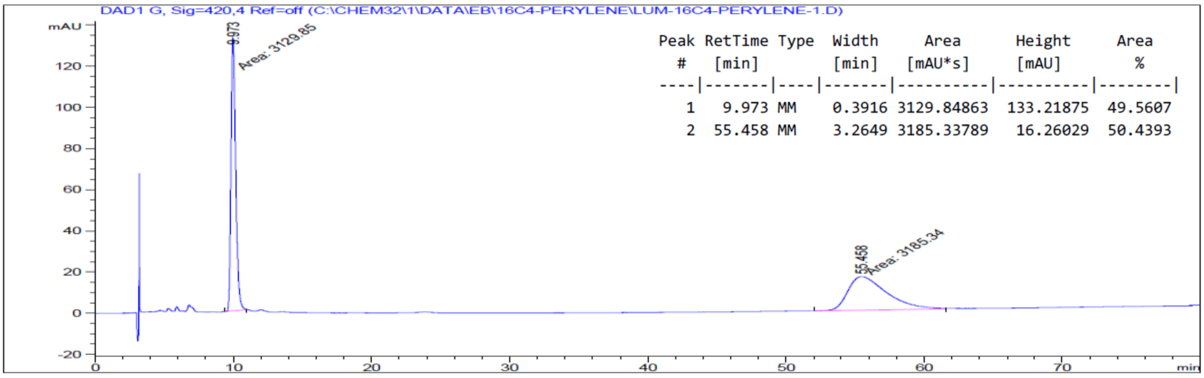

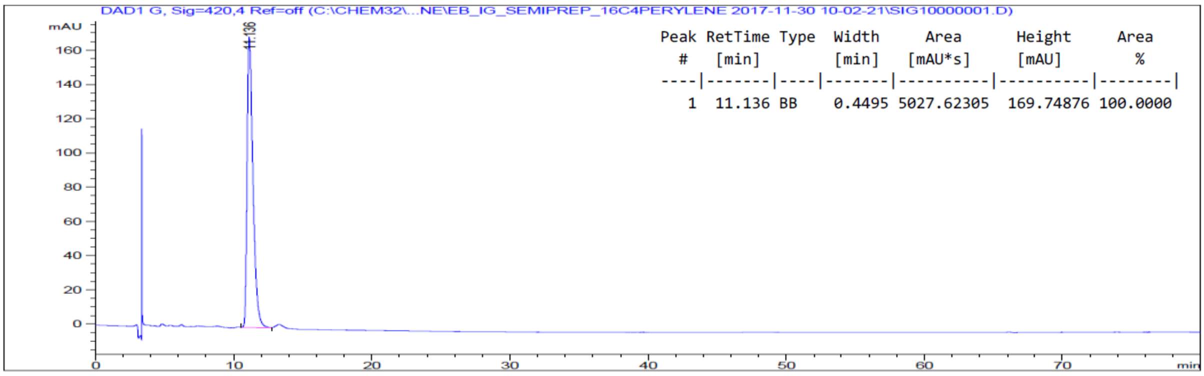

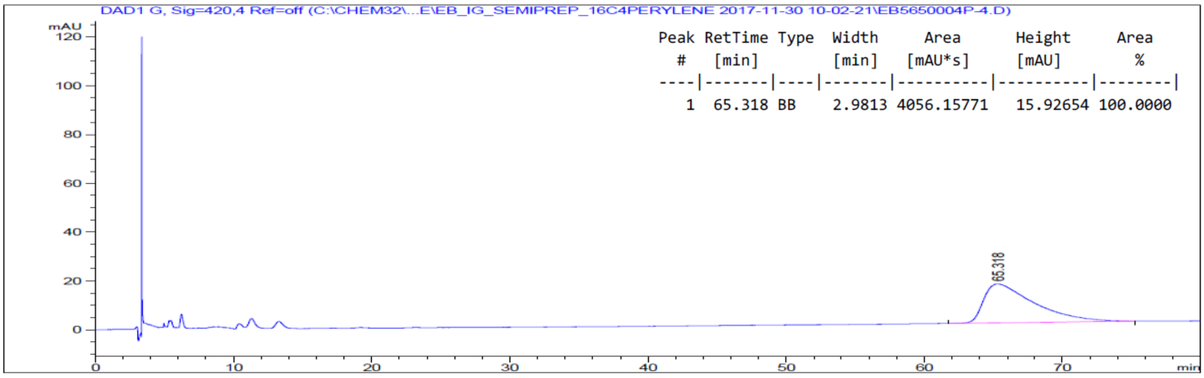

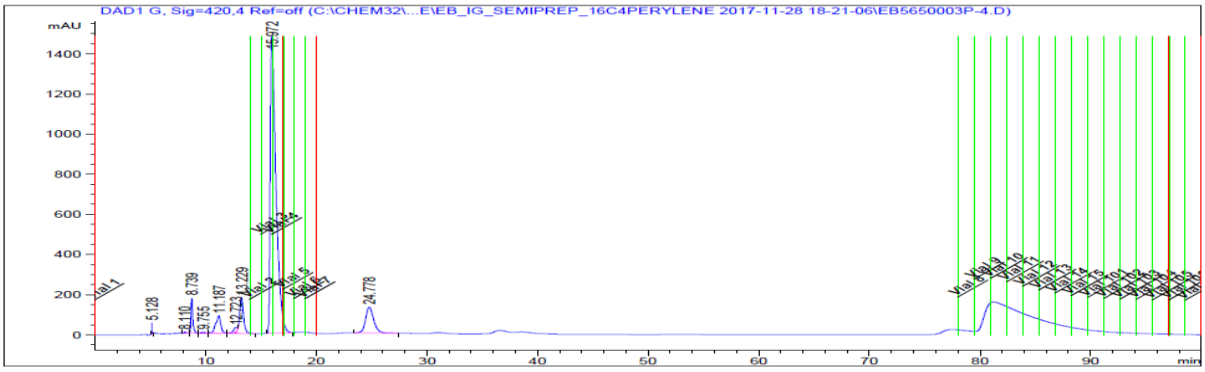

1st enantiomer: vials 3 to 5 were collected
2nd enantiomer: vials 9 to 15 were collected

3.3.7. Fluorene-18C6

CSP-HPLC: semi-preparative IG, CH_2Cl_2 (+0.1% Et_2NH)/CH_3CN (+0.1% Et_2NH) = 70:30, 2 mL/min, 20 °C, sample: 6 mg/1.2 mL of CH_2Cl_2, injection 400 µL.

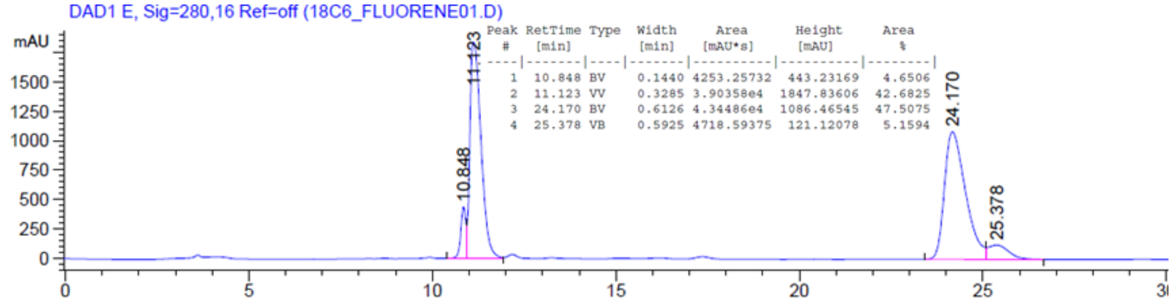

Peak #	RetTime [min]	Type	Width [min]	Area [mAU*s]	Height [mAU]	Area %
1	10.848	BV	0.1440	4253.25732	443.23169	4.6506
2	11.123	VV	0.3285	3.90358e4	1847.83606	42.6825
3	24.170	BV	0.6126	4.34486e4	1086.46545	47.5075
4	25.378	VB	0.5925	4718.59375	121.12078	5.1594

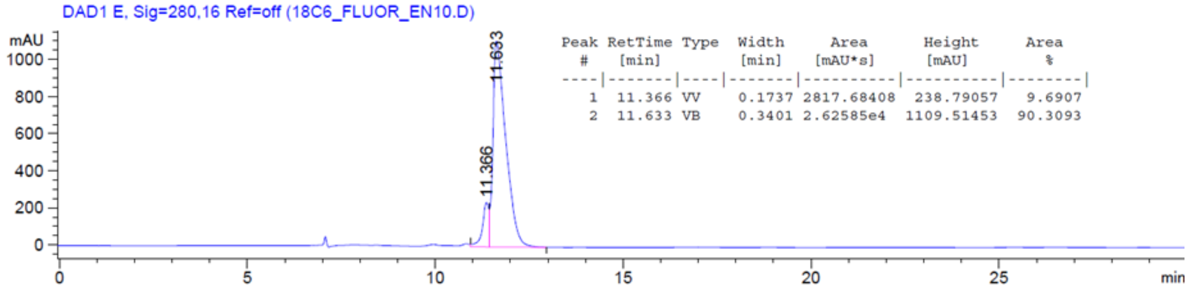

Peak #	RetTime [min]	Type	Width [min]	Area [mAU*s]	Height [mAU]	Area %
1	11.366	VV	0.1737	2817.68408	238.79057	9.6907
2	11.633	VB	0.3401	2.62585e4	1109.51453	90.3093

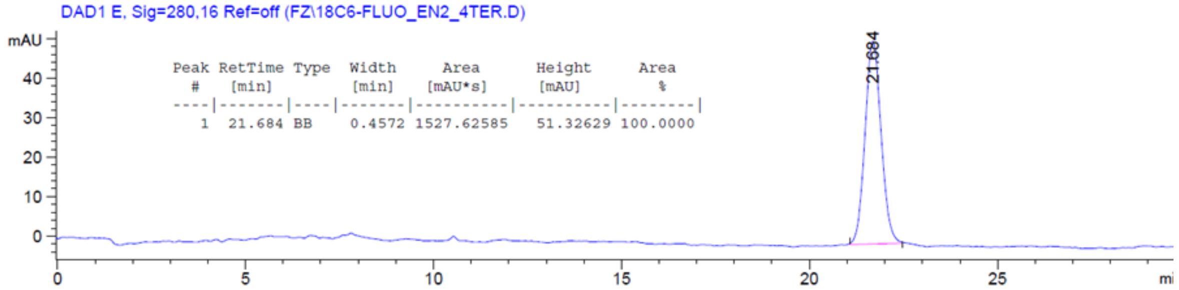

Peak #	RetTime [min]	Type	Width [min]	Area [mAU*s]	Height [mAU]	Area %
1	21.684	BB	0.4572	1527.62585	51.32629	100.0000

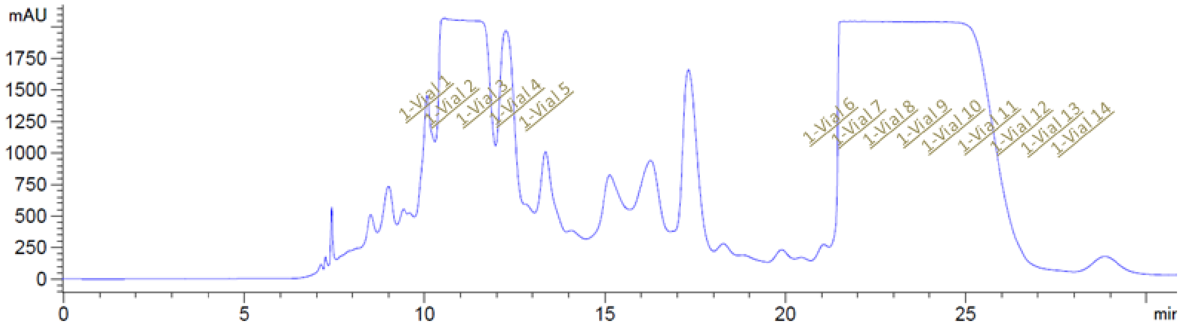

1st enantiomer: vials 1 to 3 were collected
2nd enantiomer: vials 6 to 14 were collected

3.3.8. Fluorene-18C4

CSP-HPLC: semi-preparative IG, CH_2Cl_2 (+0.1% Et_2NH)/CH_3CN (+0.1% Et_2NH) = 70:30, 3 mL/min, 20 °C, sample: 9 mg/0.5 mL of CH_2Cl_2, injection 250 µL.

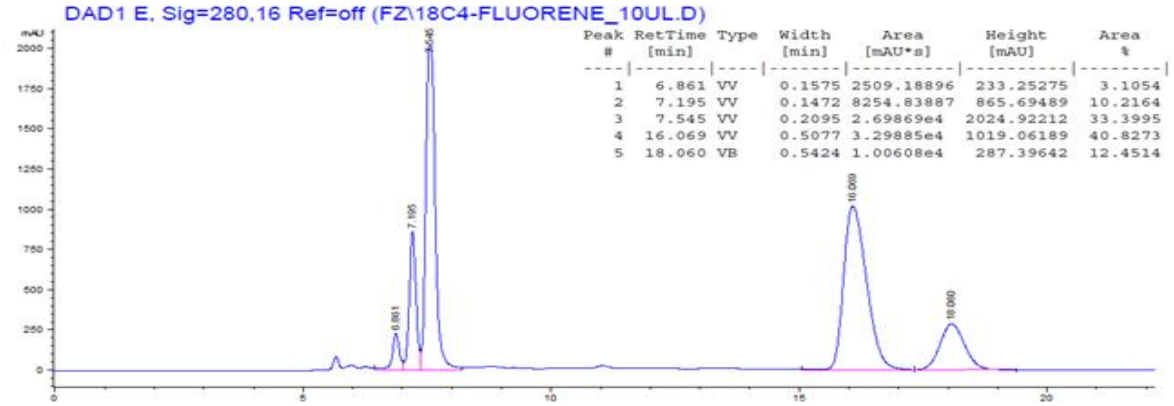

Peak #	RetTime [min]	Type	Width [min]	Area [mAU*s]	Height [mAU]	Area %
1	6.861	VV	0.1575	2509.18896	233.25275	3.1054
2	7.195	VV	0.1472	8254.83887	865.69489	10.2164
3	7.545	VV	0.2095	2.69869e4	2024.92212	33.3995
4	16.069	VV	0.5077	3.29885e4	1019.06189	40.8273
5	18.060	VB	0.5424	1.00608e4	287.39642	12.4514

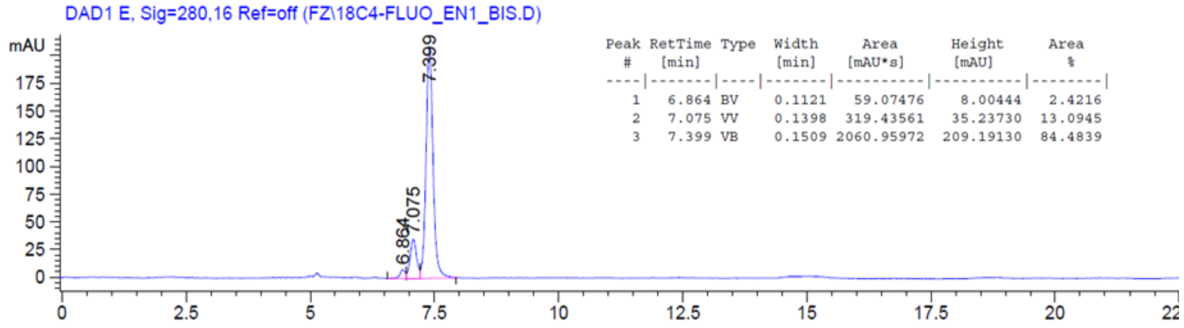

Peak #	RetTime [min]	Type	Width [min]	Area [mAU*s]	Height [mAU]	Area %
1	6.864	BV	0.1121	59.07476	8.00444	2.4216
2	7.075	VV	0.1398	319.43561	35.23730	13.0945
3	7.399	VB	0.1509	2060.95972	209.19130	84.4839

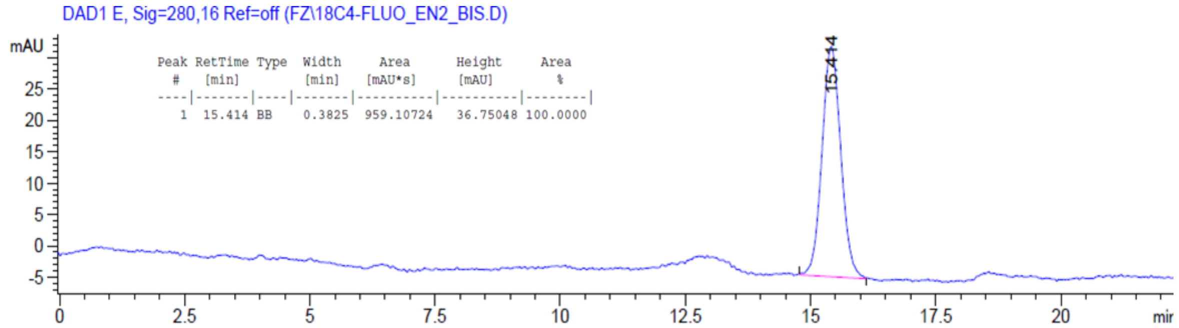

Peak #	RetTime [min]	Type	Width [min]	Area [mAU*s]	Height [mAU]	Area %
1	15.414	BB	0.3825	959.10724	36.75048	100.0000

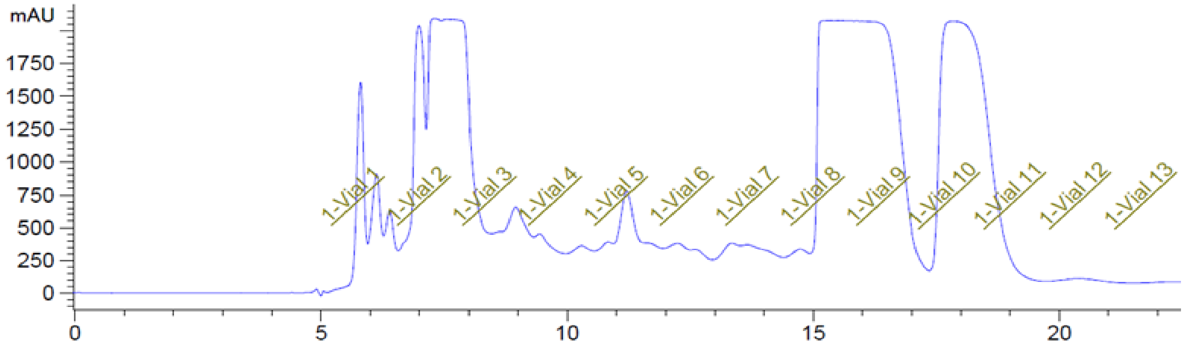

1st enantiomer: vials 2 and 3 were collected

2nd enantiomer: vials 8 and 9 were collected

3.3.9. Fluorene-16C4

CSP-HPLC: semi-preparative IG, CH_2Cl_2 (+0.1% Et_2NH)/CH_3CN (+0.1% Et_2NH) = 70:30, 3 mL/min, 20 °C, sample: 12 mg/0.5 mL of CH_2Cl_2, injection 250 µL.

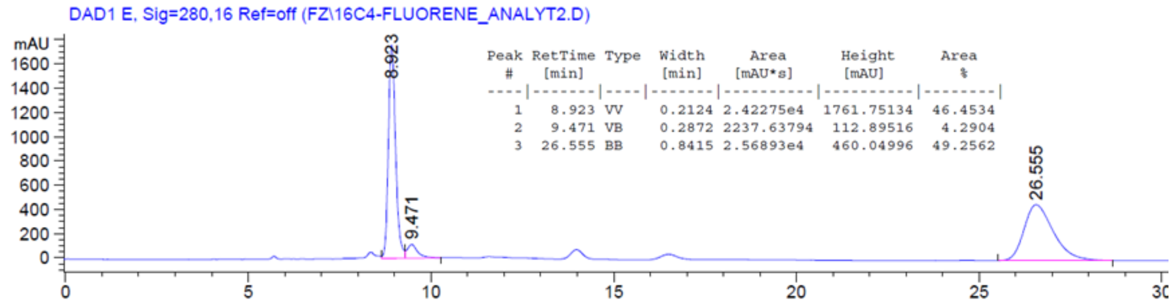

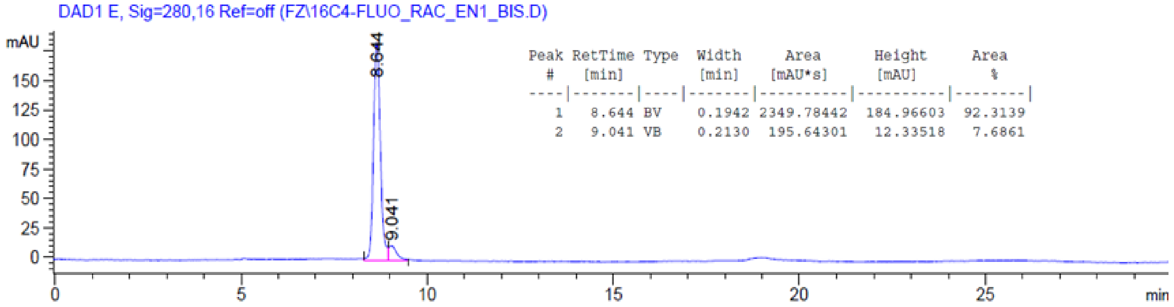

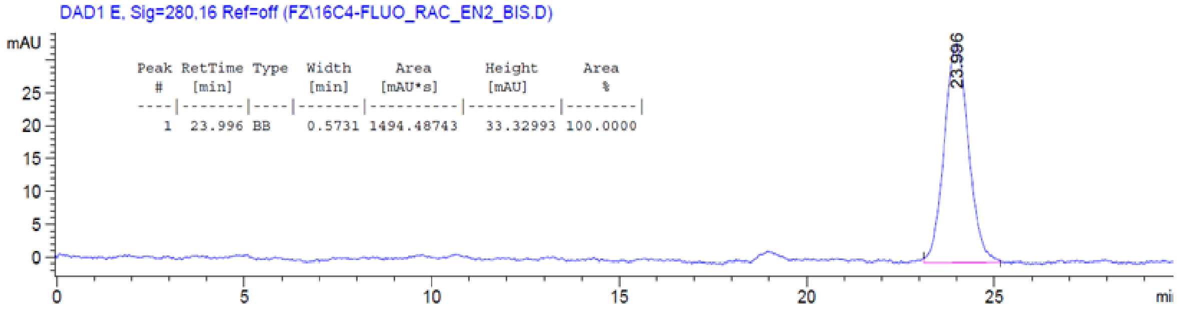

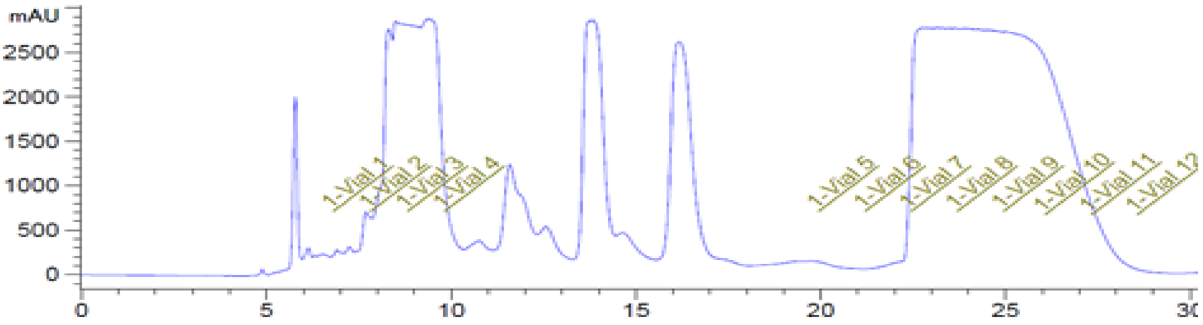

1st enantiomer: vials 1 to 4 were collected

2nd enantiomer: vials 6 to 11 were collected

3.3.10. NMI-18C6

CSP-HPLC: semi-preparative IG, CH_2Cl_2 (+0.1% Et_2NH)/CH_3CN (+0.1% Et_2NH) = 90:10, 2 mL/min, 20 °C, sample: 20 mg/1.5 mL of CH_2Cl_2, injection 500 µL.

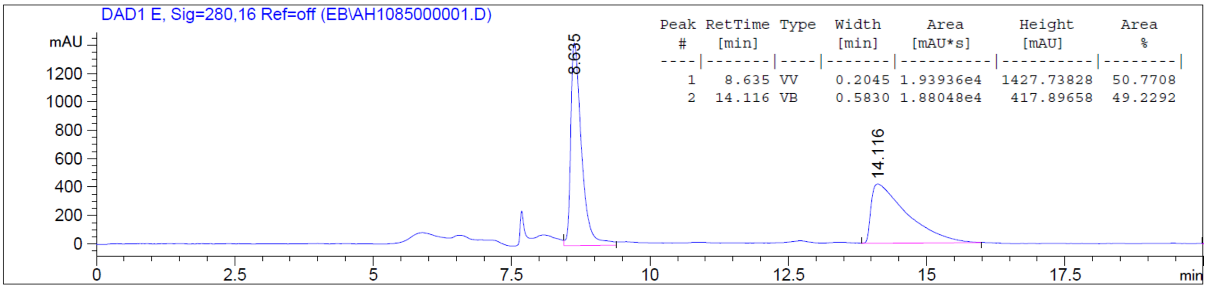

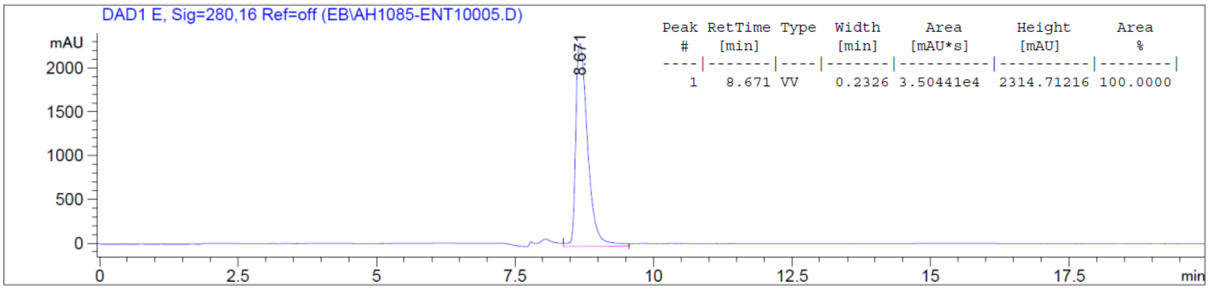

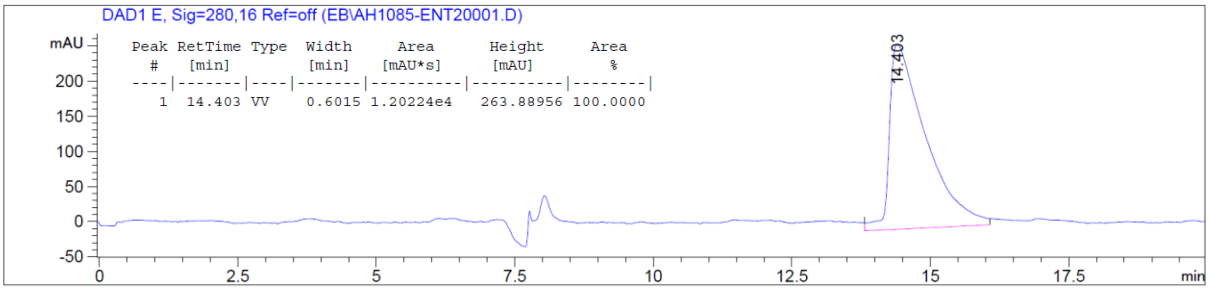

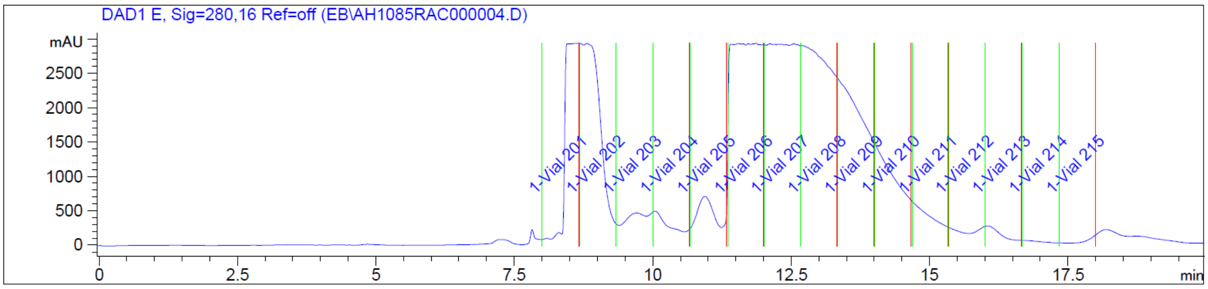

1st enantiomer: vials 1 and 2 were collected

2nd enantiomer: vials 6 to 11 were collected

4. Spectroscopic data

4.1. UV-Vis absorption and fluorescence spectra

4.1.1. Procedure

In a typical experiment, UV-Vis absorbance and fluorescence spectra of a solution of interest compound (ca. 10^{-5} M) in acetonitrile or CH_2Cl_2 were recorded in a 1 cm cell. For the complexation experiments, an excess of $Ba(ClO_4)_2$ or $NaBAr_F$ was added to the solution and the UV-Vis absorbance and fluorescence spectra were recorded again.

4.1.2. Summary of the optical properties

Table S3. Spectroscopic data without and with cation[a]

Entry	Compound	λ_{max} (nm)			ϕ	
		absorption	(monomer) emission	excimer emission	Without M^{n+}	With M^{n+}
1	pyrene-monomer	341	387	-	24% [b]	-
2	pyrene-18C6	345	387	490	24%[b]	<1%[b]
3	pyrene-18C4	348	387	481	46%[b]	<1%[b]
4	pyrene-16C4	350	387	491	32%[b]	<1%[b]
5	perylene-monomer	443	472	-	77% [d]	-
6	perylene-18C6	446	470	543	10%[d]	62%[d]
7	perylene-18C4	446	470	536	19%[d]	59%[d]
8	perylene-16C4	446	470	tailed	16%[d]	50%[d]
9	fluorene-monomer	288	335	-	18% [c]	-
10	fluorene-18C6	318	-	337	2%[c]	-
11	fluorene-18C4	316	-	338	2%[c]	-
12	fluorene-16C4	316	-	336	2%[c]	-
13	NMI-monomer	338	420	-	17%	-
14	NMI-18C6	340	386	485	10%[b]	18%[b]

[a] $Ba(ClO_4)_2/CH_3CN$ system used for **18C6** derivatives, $NaBAr_F/CH_2Cl_2$ system used for **18C4** and **16C4** compounds; [b] Relative to anthracene (ϕ = 27% in EtOH); [c] Relative to phenanthrene (ϕ = 12.5% in EtOH); [d] Relative to coumarine 153 (ϕ = 38% in EtOH).

4.1.3. Spectra of the monomeric amides without and with $Ba(ClO_4)_2$

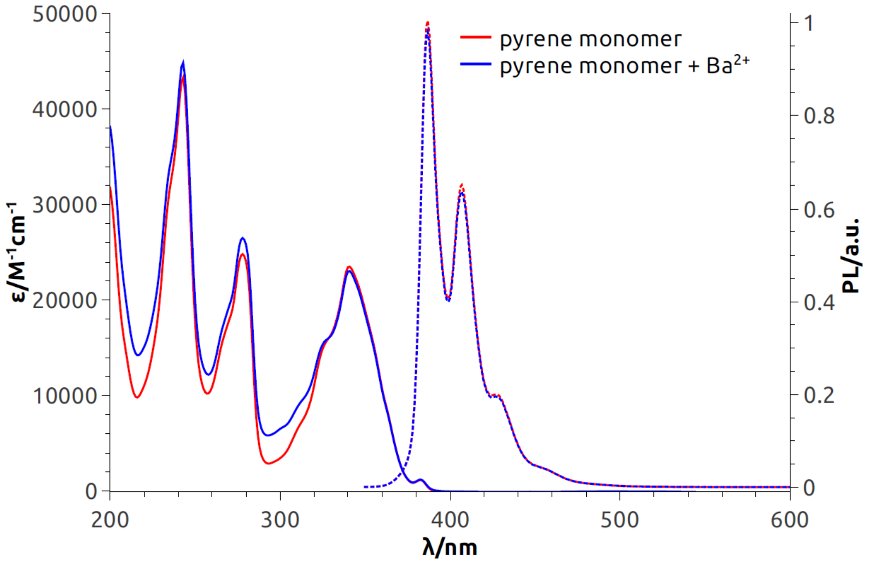

Figure S1. Absorption (continuous lines) and fluorescence (dotted lines) spectra of **pyrene monomer** in acetonitrile without (red) or with (blue) $Ba(ClO_4)_2$ (2 equiv).

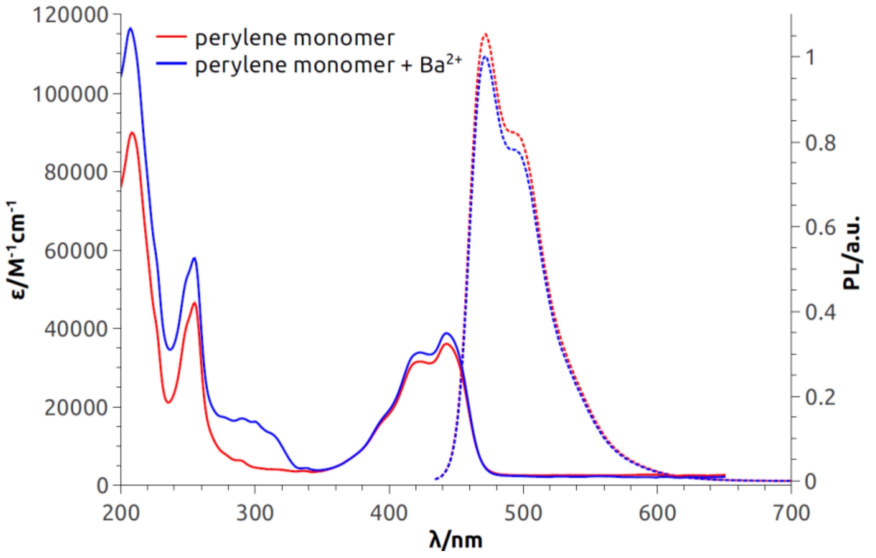

Figure S2. Absorption (continuous lines) and fluorescence (dotted lines) spectra of **perylene monomer** in acetonitrile without (red) or with (blue) $Ba(ClO_4)_2$ (2 equiv).

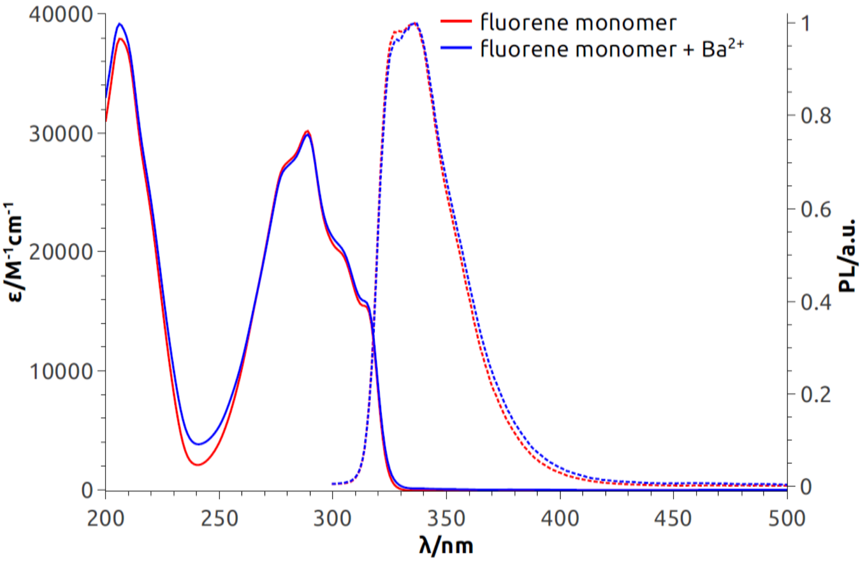

Figure S3. Absorption (continuous lines) and fluorescence (dotted lines) spectra of **fluorene monomer** in acetonitrile without (red) or with (blue) $Ba(ClO_4)_2$ (2 equiv).

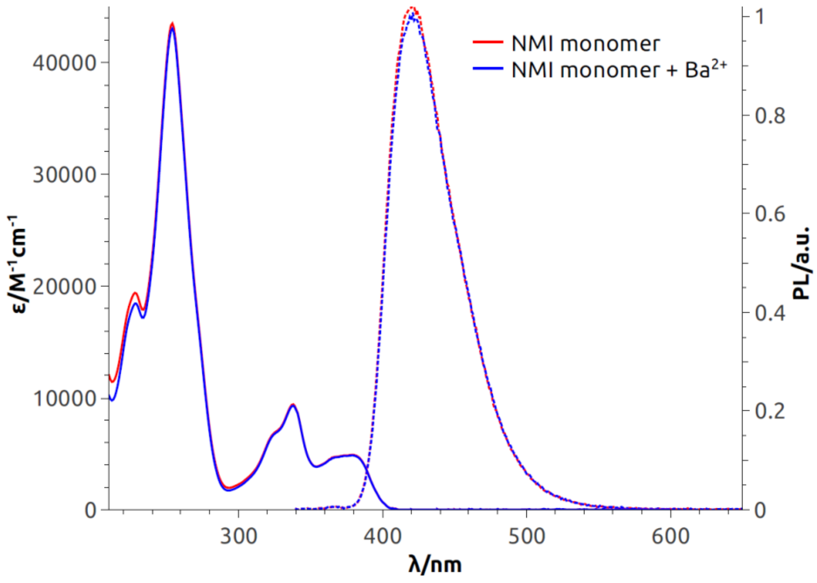

Figure S4. Absorption (continuous lines) and fluorescence (dotted lines) spectra of **NMI monomer** in acetonitrile without (red) or with (blue) $Ba(ClO_4)_2$ (2 equiv).

4.1.4. Spectra of the macrocycles without and with cations

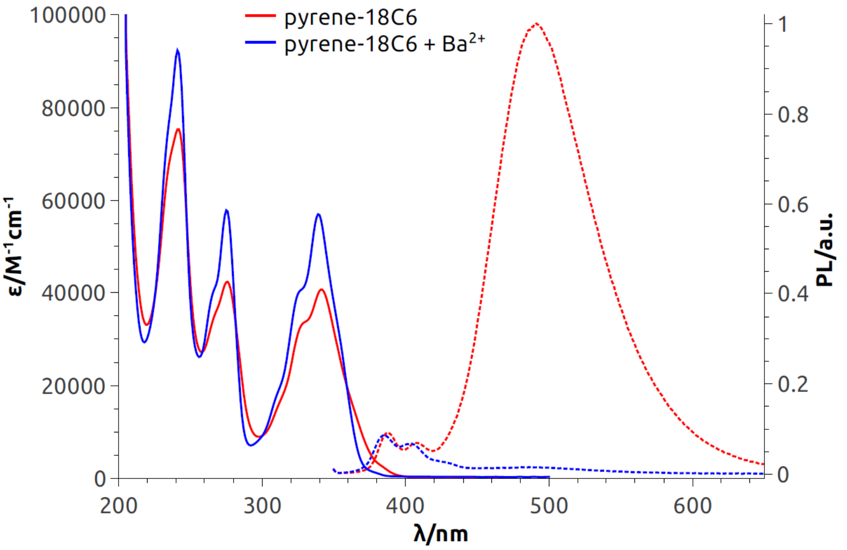

Figure S5. Absorption (continuous lines) and fluorescence (dotted lines) spectra of **pyrene-18C6** in acetonitrile without (red) or with (blue) $Ba(ClO_4)_2$ (2 equiv).

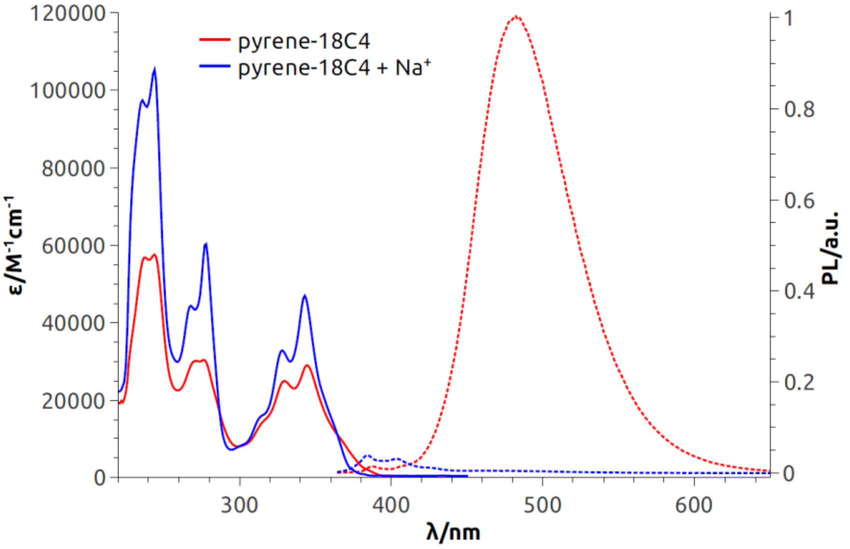

Figure S6. Absorption (continuous lines) and fluorescence (dotted lines) spectra of **pyrene-18C4** in CH_2Cl_2 without (red) or with (blue) $NaBAr_F$ (2 equiv).

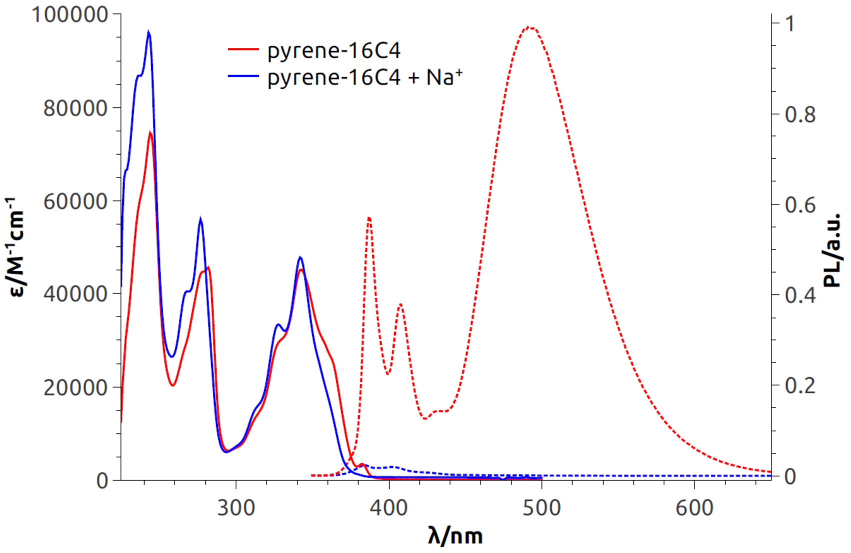

Figure S7. Absorption (continuous lines) and fluorescence (dotted lines) spectra of **pyrene-16C4** in CH_2Cl_2 without (red) or with (blue) $NaBAr_F$ (2 equiv).

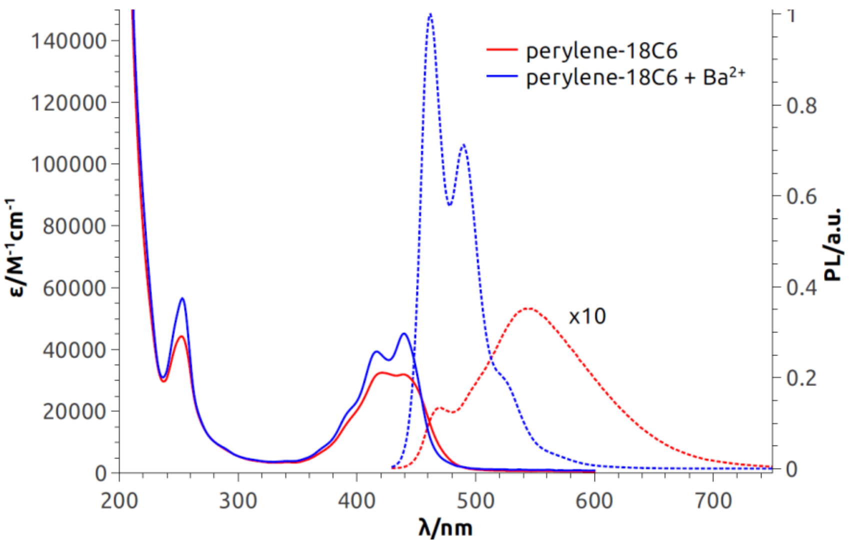

Figure S8. Absorption (continuous lines) and fluorescence (dotted lines) spectra of **perylene-18C6** in acetonitrile without (red) or with (blue) $Ba(ClO_4)_2$ (2 equiv).

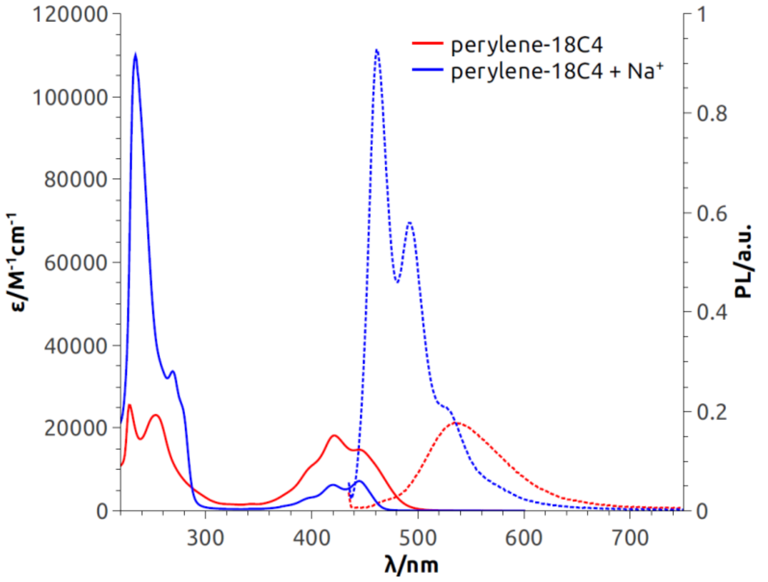

Figure S9. Absorption (continuous lines) and fluorescence (dotted lines) spectra of **perylene-18C4** in CH_2Cl_2 without (red) or with (blue) $NaBAr_F$ (2 equiv).

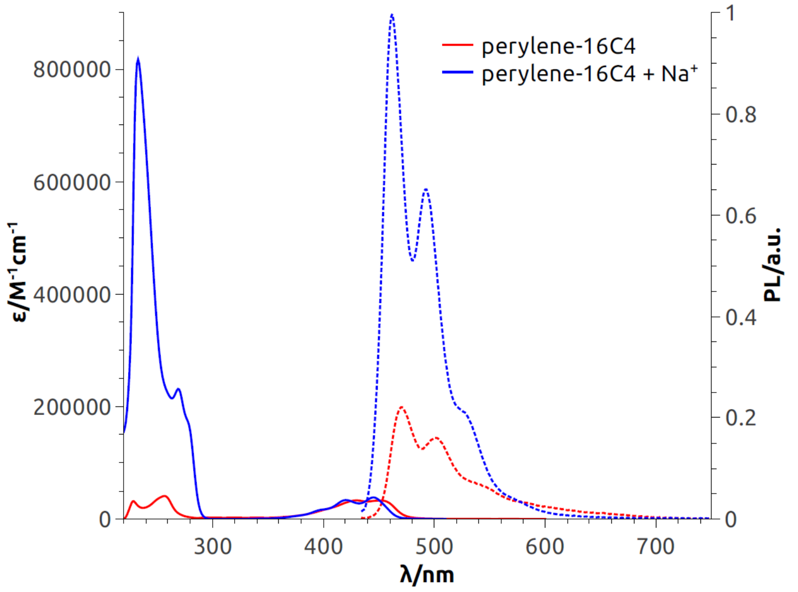

Figure S10. Absorption (continuous lines) and fluorescence (dotted lines) spectra of **perylene-16C4** in CH_2Cl_2 without (red) or with (blue) $NaBAr_F$ (2 equiv).

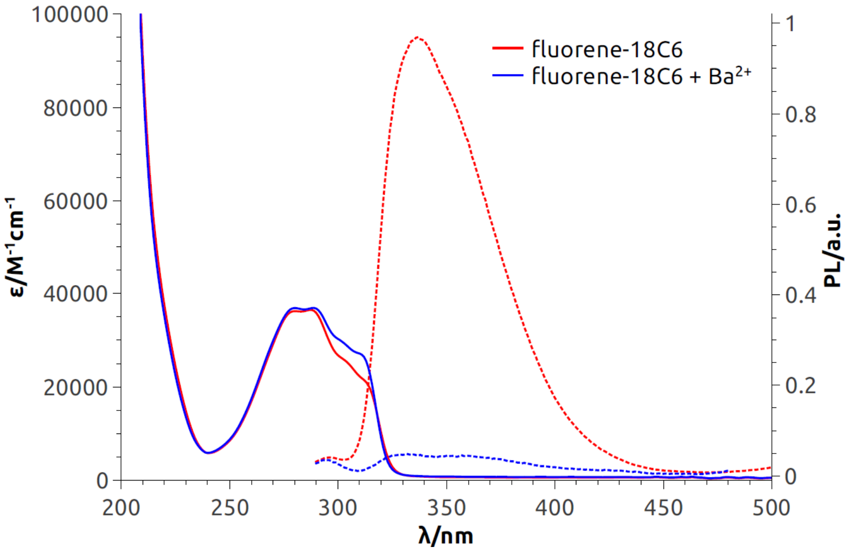

Figure S11. Absorption (continuous lines) and fluorescence (dotted lines) spectra of **fluorene-18C6** in acetonitrile without (red) or with (blue) of $Ba(ClO_4)_2$ (2 equiv).

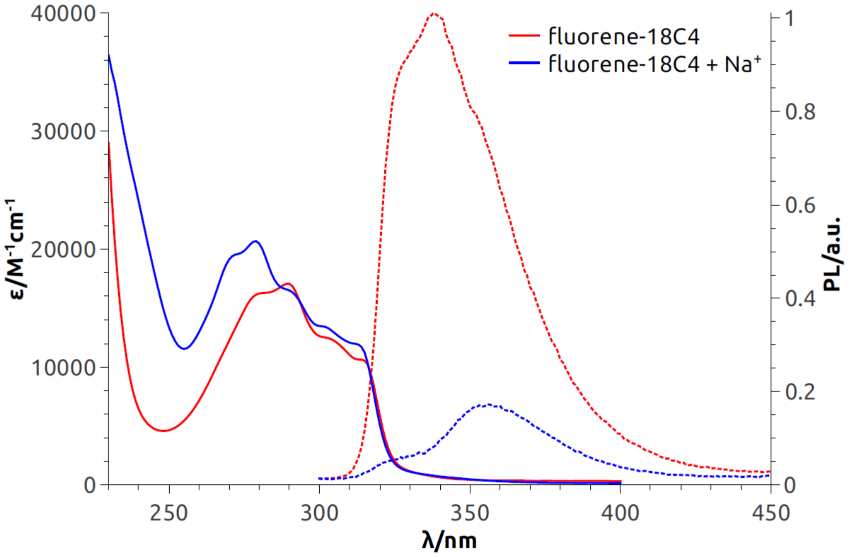

Figure S12. Absorption (continuous lines) and fluorescence (dotted lines) spectra of **fluorene-18C4** in CH_2Cl_2 without (red) or with (blue) $NaBAr_F$ (2 equiv).

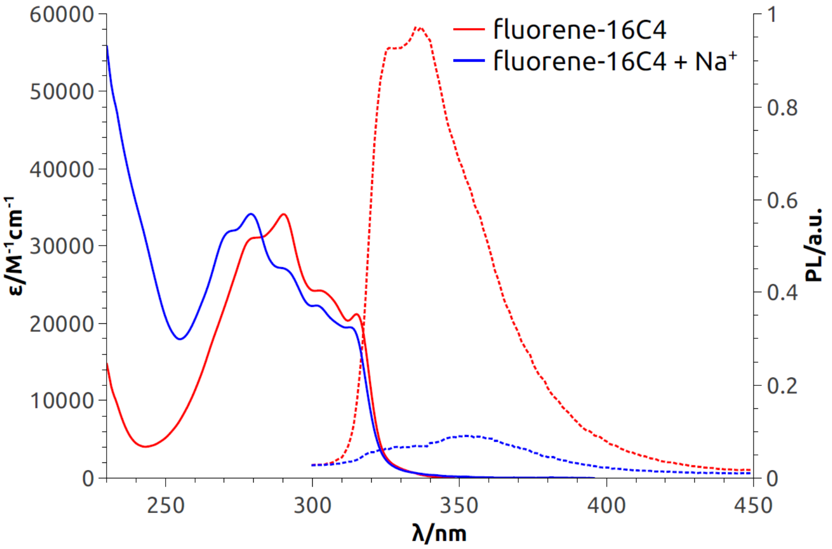

Figure S13. Absorption (continuous lines) and fluorescence (dotted lines) spectra of **fluorene-16C4** in CH_2Cl_2 without (red) or with (blue) $NaBAr_F$ (2 equiv).

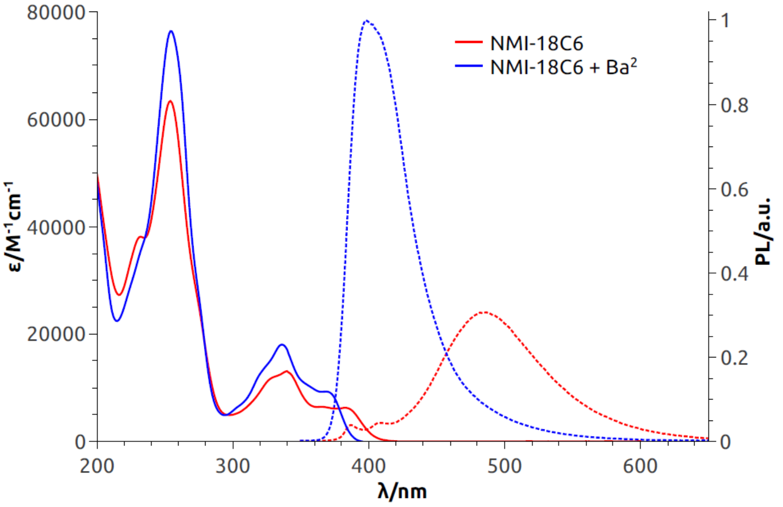

Figure S14. Absorption (continuous lines) and fluorescence (dotted lines) spectra of **NMI-18C6** in acetonitrile without (red) or with (blue) of $Ba(ClO_4)_2$ (2 equiv).

4.2. ECD spectra

4.2.1. General procedure

In a typical experiment, the ECD spectrum of a solution of enantiopure compound (ca. 10^{-5} M) in acetonitrile or CH_2Cl_2 was recorded in a 1 cm cell. For the complexation experiments, 3.0 equivalents (10^{-3} M stock solutions) of $NaBAr_F$ in dichloromethane or $Ba(ClO_4)_2$ in acetonitrile were added to the solution and the ECD spectrum was recorded again.

4.2.2. ECD spectra without and with NaBAr$_F$

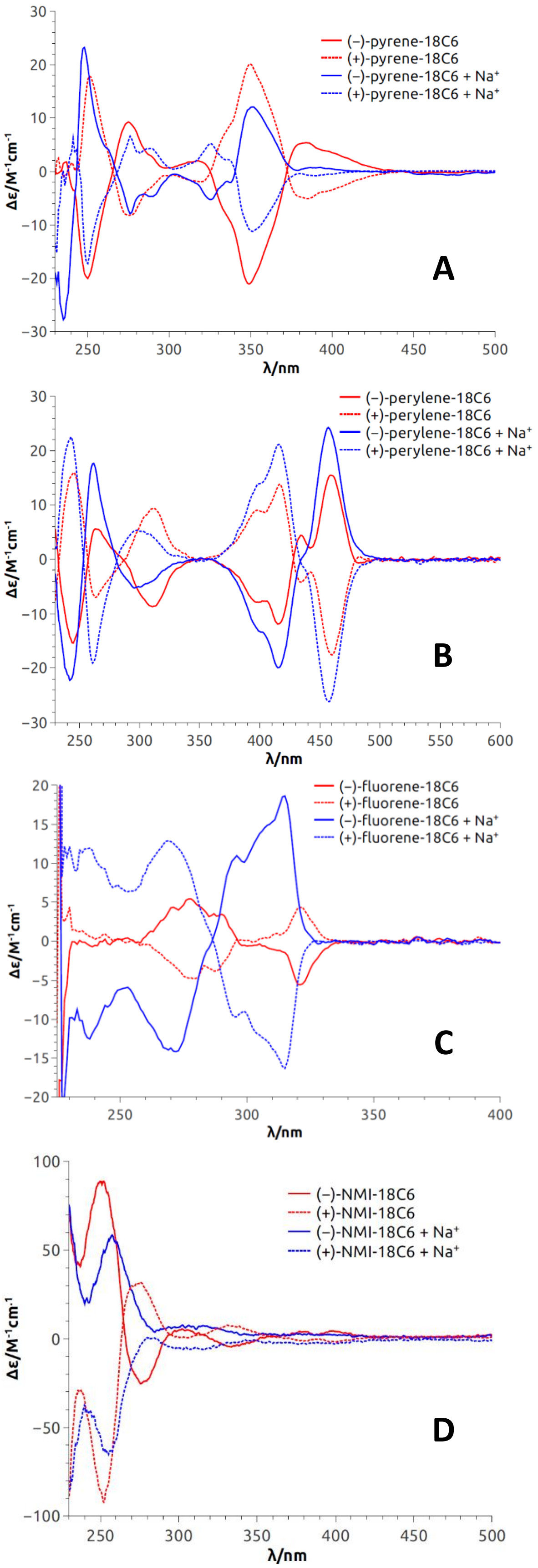

Figure S15. ECD spectra of **pyrene-18C6** (A), **perylene-18C6** (B), **fluorene-18C6** (C) and **NMI-18C6** (D)-in CH_2Cl_2 without (red) or with (blue) NaBAr$_F$.

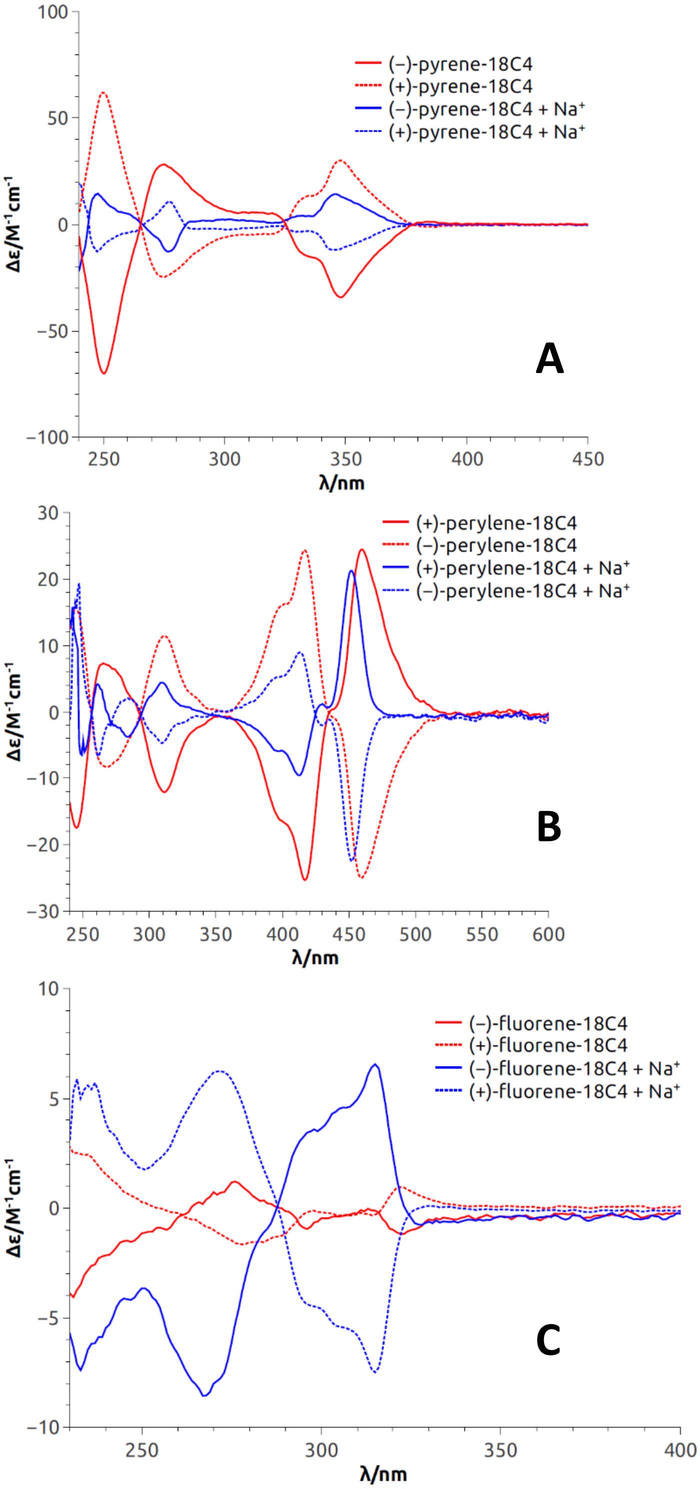

Figure S16. ECD spectra of **pyrene-18C4** (A)**, perylene-18C4** (B) and **fluorene-18C4** (C) in CH_2Cl_2 without (red) or with (blue) $NaBAr_F$.

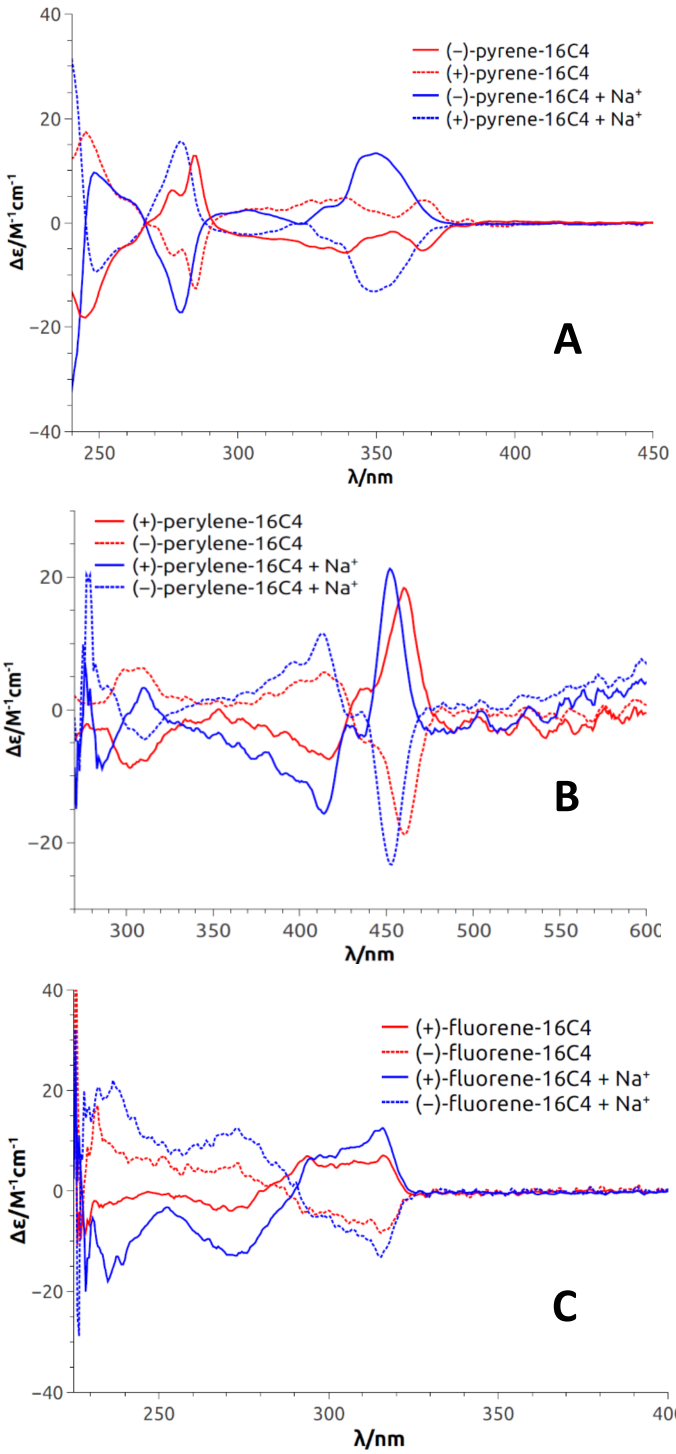

Figure S17. ECD spectra of **pyrene-16C4** (A)**, perylene-16C4** (B) and **fluorene-16C4** (C) in CH_2Cl_2 without (red) or with (blue) $NaBAr_F$.

4.3. CPL spectra

4.3.1. General procedure

In a typical experiment, the CPL spectrum of a solution of enantiopure compound (ca. 10^{-5} M) in acetonitrile or CH_2Cl_2 was recorded in a 1 cm cell. For the complexation experiments, 3.0 equivalents (10^{-3} M stock solutions) of $NaBAr_F$ in dichloromethane were added to the solution and the CPL spectrum was recorded again.

4.3.2. CPL spectra without and with $NaBAr_F$

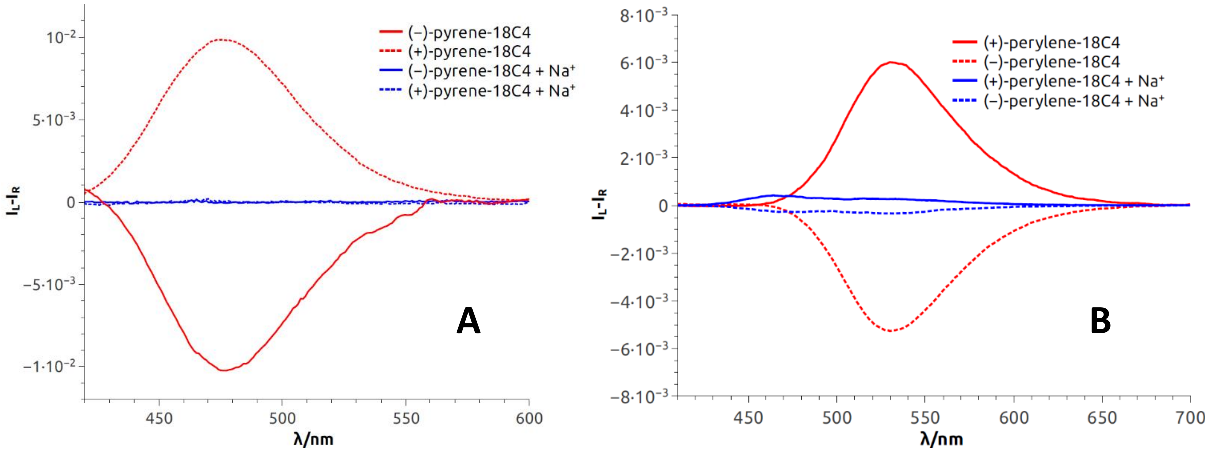

Figure S18. CPL spectra of **pyrene-18C4** (A) and **perylene-18C4** (B) in CH_2Cl_2 without (red) and with (blue) $NaBAr_F$.

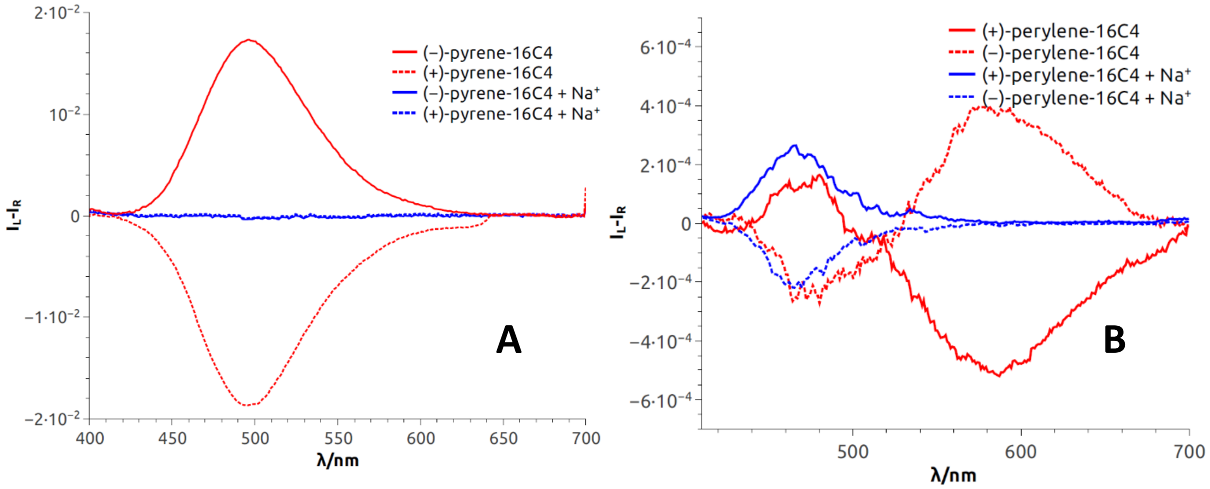

Figure S19. CPL spectra of **pyrene-16C4** (A) and **perylene-16C4** (B) in CH_2Cl_2 without (red) and with (blue) $NaBAr_F$.

4.4. Reversibility

4.4.1. Procedure

In a typical experiment, ECD/fluorescence/CPL spectrum of a solution of enantiopure compound (ca. 10^{-5} M) in CH_2Cl_2 was recorded. For the complexation experiments, 2 equiv of $NaBAr_F$ from a 10^{-3} M solution in CH_2Cl_2 were added to the solution and the spectrum was recorded again. To switch back the system, 2 equiv of 18-Crown-6 from a 10^{-3} M solution in CH_2Cl_2 were added to the solution and the spectrum was recorded again. The procedure was repeated over several cycles.

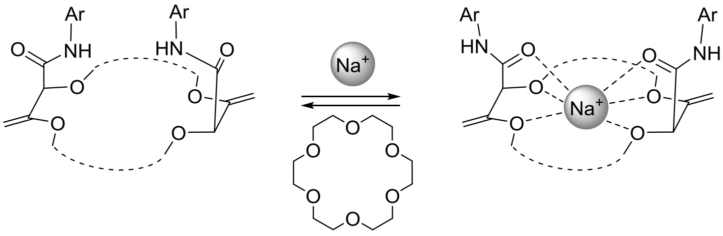

(S2)

4.4.2. ECD reversibility

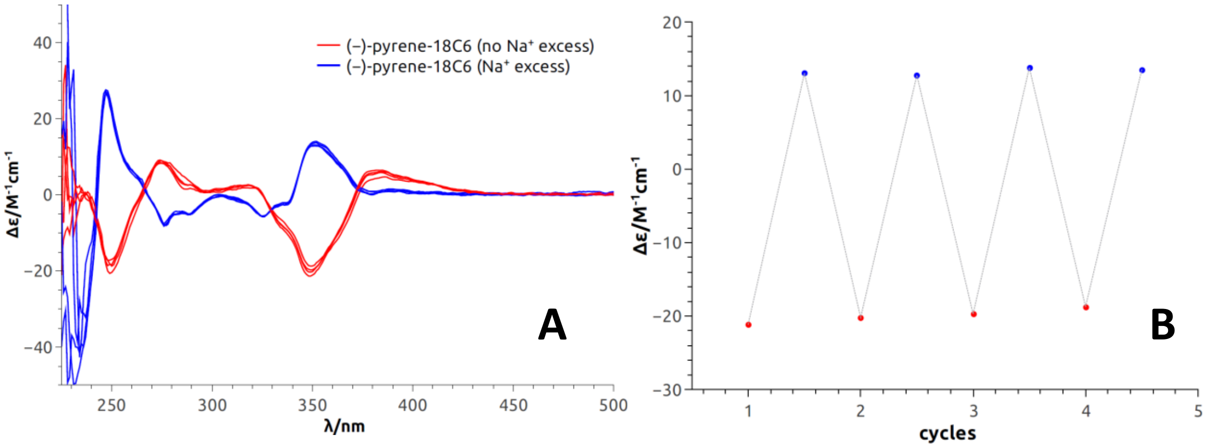

Figure S20. Reversible ECD (A) and intensities at 345 nm (B) for the first eluted (−)-enantiomer of **pyrene-18C6** over 4 cycles of $NaBAr_F$/18-Crown-6 additions (red/blue: without/with $NaBAr_F$).

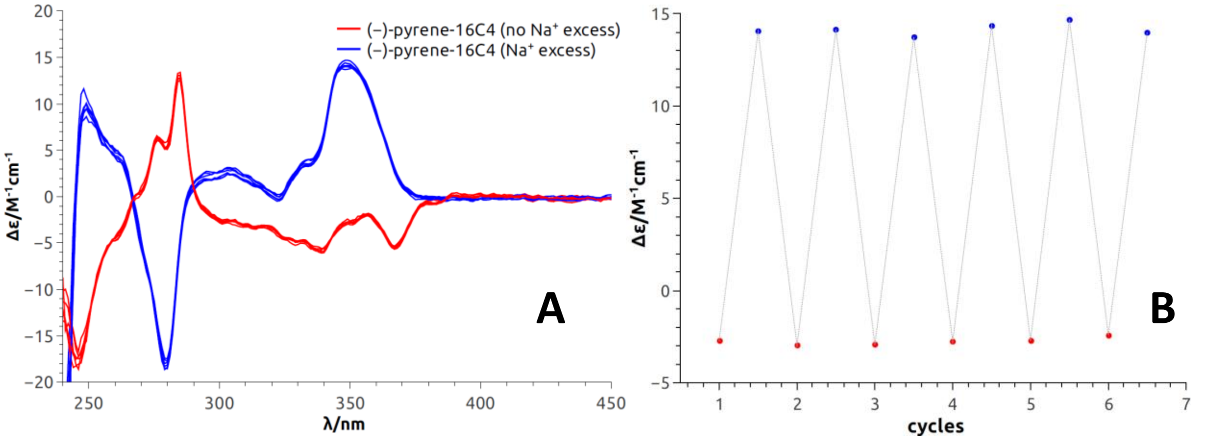

Figure S21. Reversible ECD (A) and intensities at 342 nm (B) for the first eluted (−)-enantiomer of **pyrene-16C4** over 6 cycles of $NaBAr_F$/18-Crown-6 additions (red/blue: without/with $NaBAr_F$).

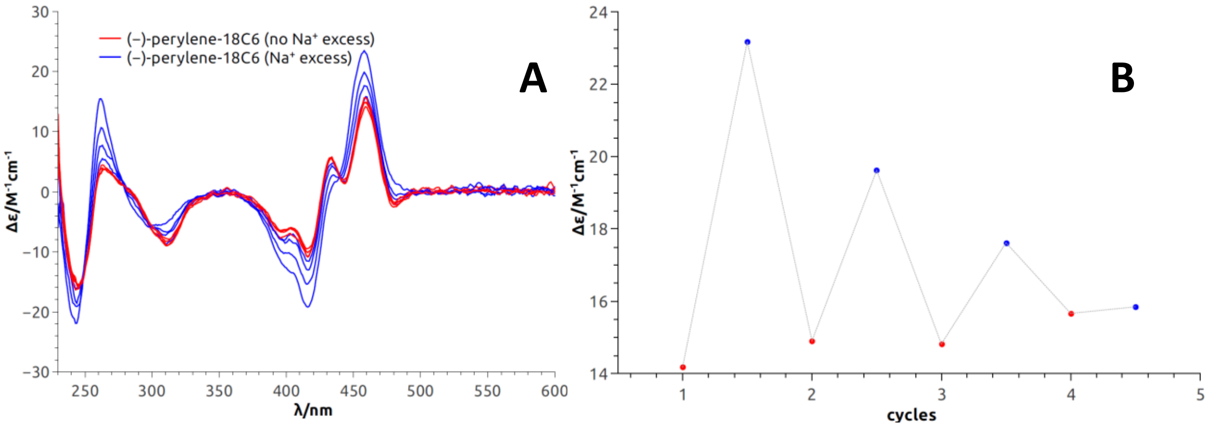

Figure S22. Reversible ECD (A) and intensities at 446 nm (B) for the first eluted (−)-enantiomer of **perylene-18C6** over 4 cycles of $NaBAr_F$/18-Crown-6 additions (red/blue: without/with $NaBAr_F$).

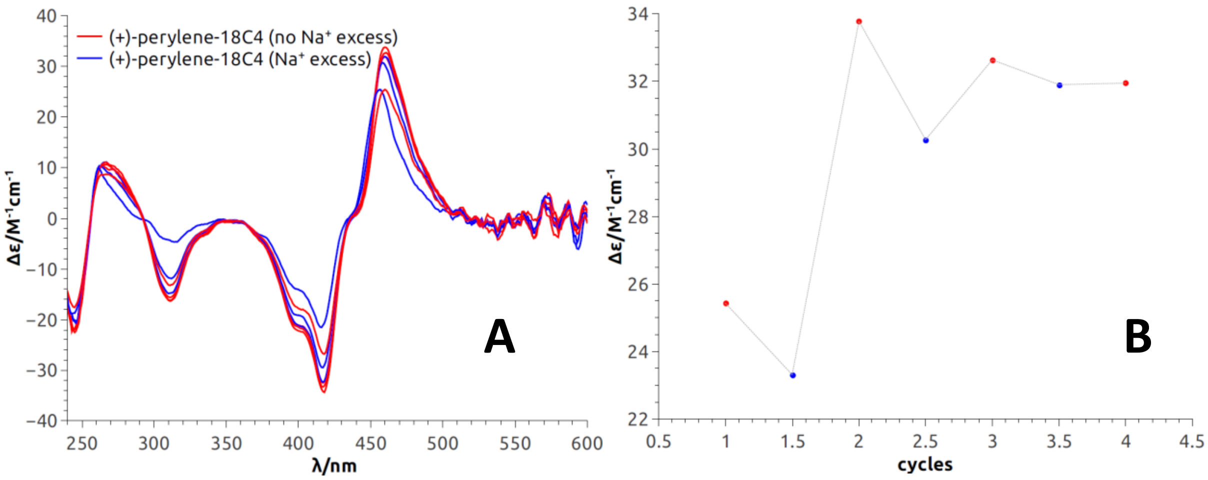

Figure S23. Reversible ECD (A) and intensities at 446 nm (B) for the first eluted (+)-enantiomer of **perylene-18C4** over 4 cycles of $NaBAr_F$/18-Crown-6 additions (red/blue: without/with $NaBAr_F$).

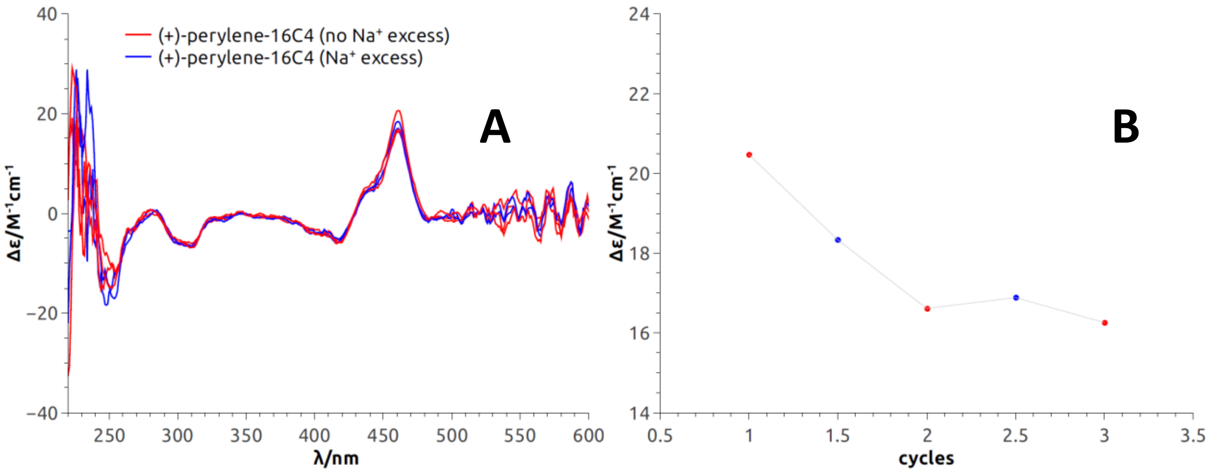

Figure S24. Reversible ECD (A) and intensities at 446 nm (B) for the first eluted (+)-enantiomer of **perylene-16C4** over 4 cycles of NaBAr$_F$/18C6 additions (red/blue: without/with NaBAr$_F$).

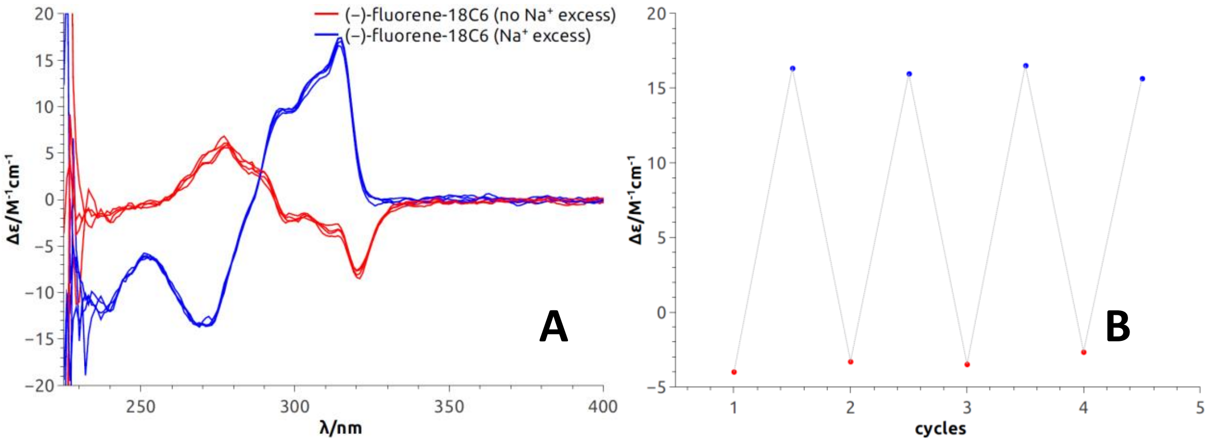

Figure S25. Reversible ECD (A) and intensities at 314 nm (B) for the first eluted (−)-enantiomer of **fluorene-18C6** over 4 cycles of NaBAr$_F$/18-Crown-6 additions (red/blue: without/with NaBAr$_F$).

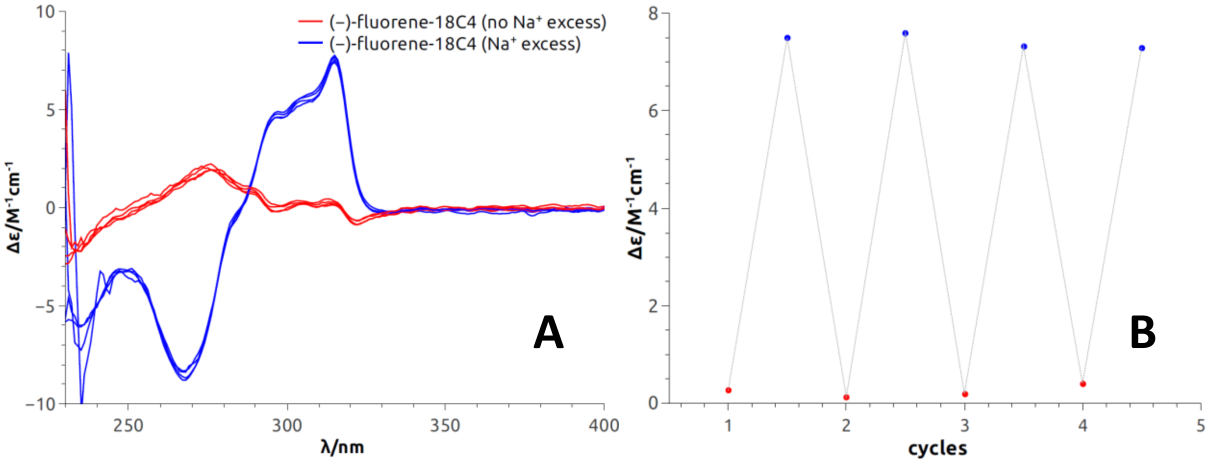

Figure S26. Reversible ECD (A) and intensities at 315 nm (B) for the first eluted (−)-enantiomer of **fluorene-18C4** over4 cycles of $NaBAr_F$/18-Crown-6 additions (red/blue: without/with $NaBAr_F$).

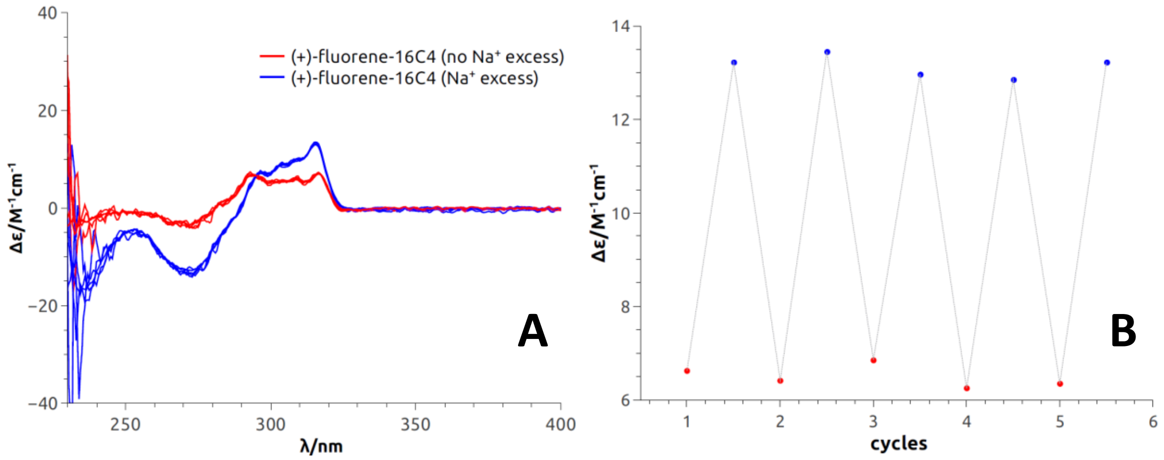

Figure S27. Reversible ECD (A) and intensities at 316 nm (B) for the first eluted (+)-enantiomer of **fluorene-16C4** over 5 cycles of $NaBAr_F$/18-Crown-6 additions (red/blue: without/with $NaBAr_F$).

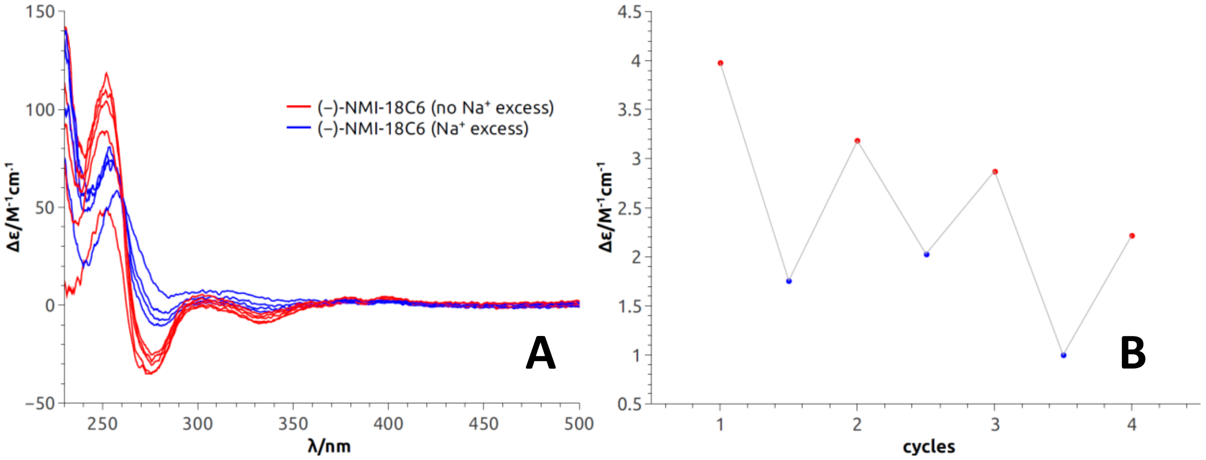

Figure S28. Reversible ECD (A) and intensities at 398 nm (B) for the first eluted (−)-enantiomer of **NMI-18C6** over 4 cycles of $NaBAr_F$/18-Crown-6 additions (red/blue: without/with $NaBAr_F$).

4.4.3. Fluorescence reversibility

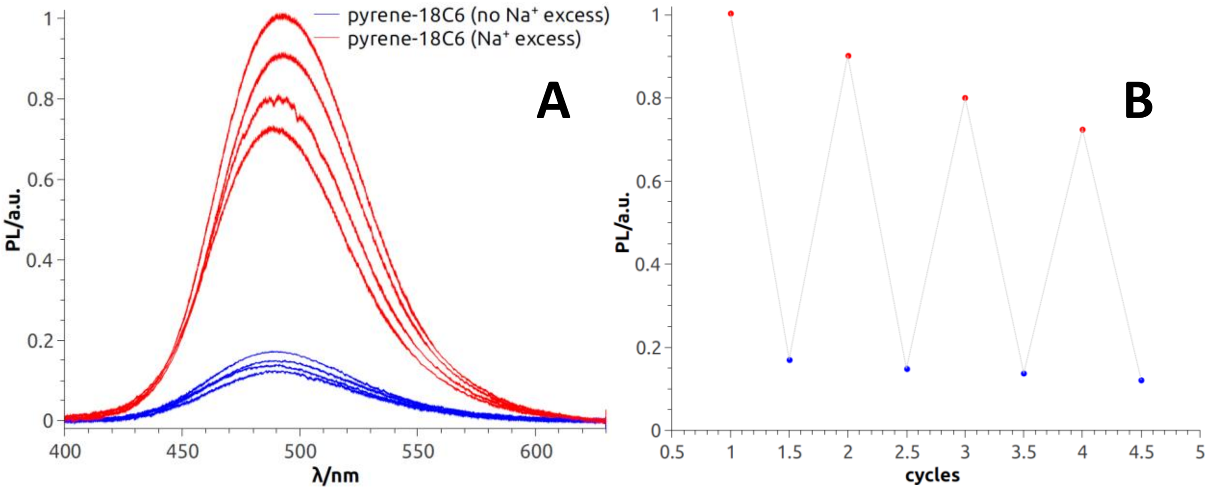

Figure S29. Reversible fluoresence (A) and intensities at 490 nm (B) for **pyrene-18C6** over 4 cycles of $NaBAr_F$/18-Crown-6 additions (red/blue: without/with $NaBAr_F$).

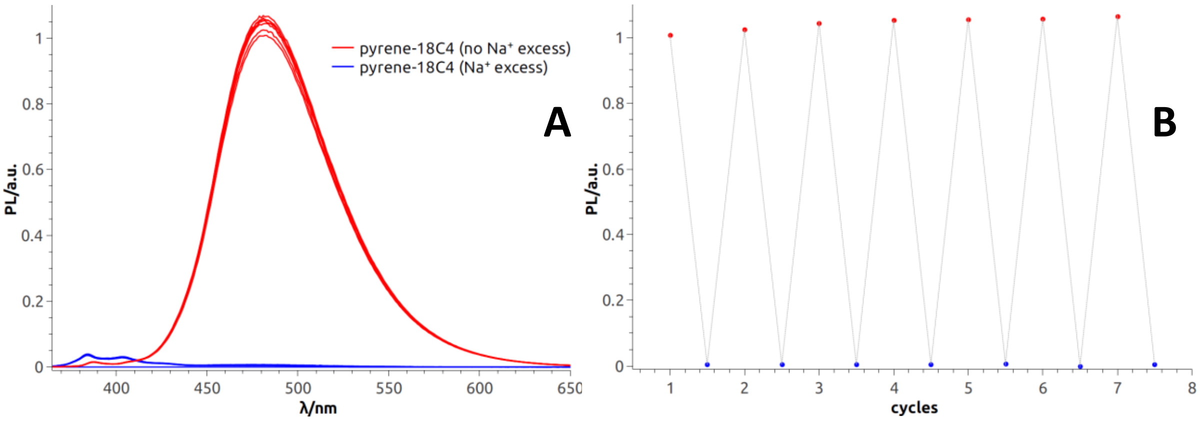

Figure S30. Reversible fluoresence (A) and intensities at 481 nm (B) for **pyrene-18C4** over 7 cycles of $NaBAr_F$/18-Crown-6 additions (red/blue: without/with $NaBAr_F$).

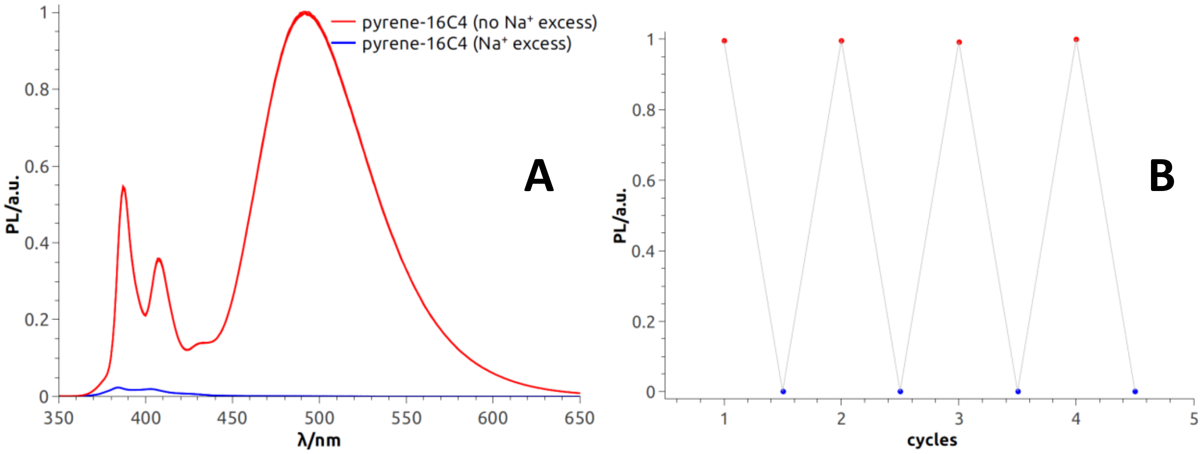

Figure S31. Reversible fluoresence (A) and intensities at 491 nm (B) for **pyrene-16C4** over 4 cycles of $NaBAr_F$/18-Crown-6 additions (red/blue: without/with $NaBAr_F$).

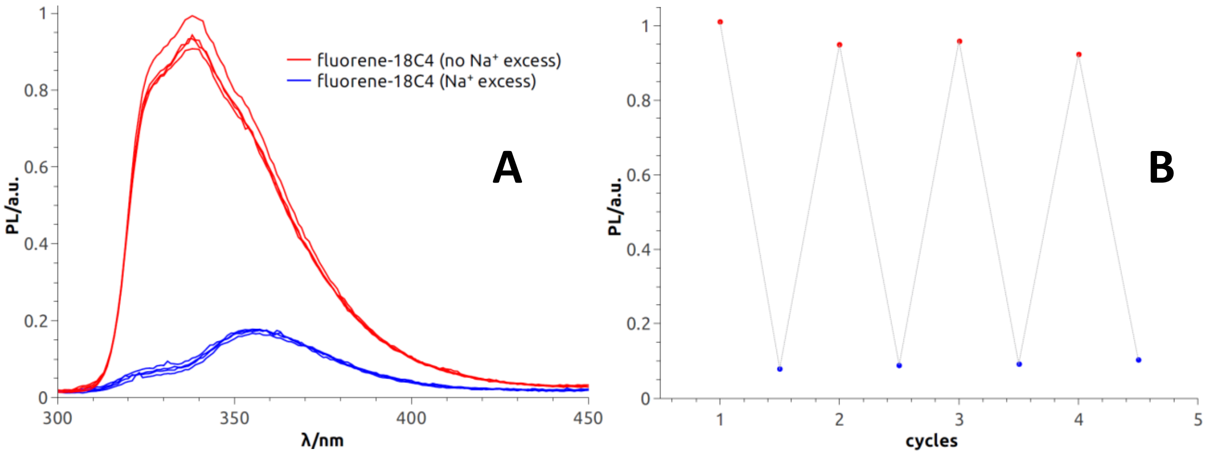

Figure S32. Reversible fluoresence (A) and intensities at 338 nm (B) for **fluorene-18C4** over 4 cycles of $NaBAr_F$/18-Crown-6 additions (red/blue: without/with $NaBAr_F$).

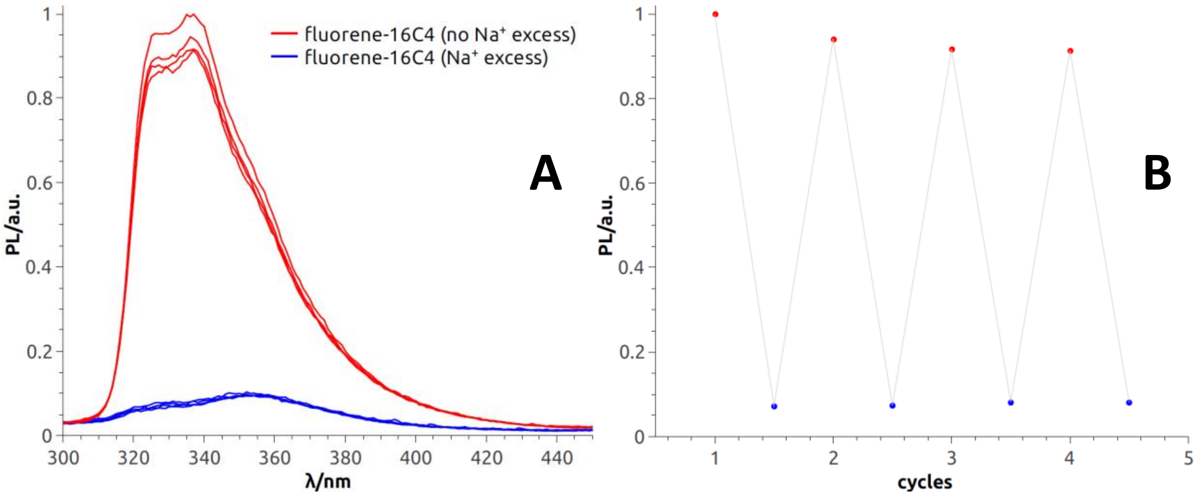

Figure S33. Reversible fluoresence (A) and intensities at 336 nm (B) for **fluorene-16C4** over 4 cycles of $NaBAr_F$/18-Crown-6 additions (red/blue: without/with $NaBAr_F$).

4.4.4. CPL reversibility

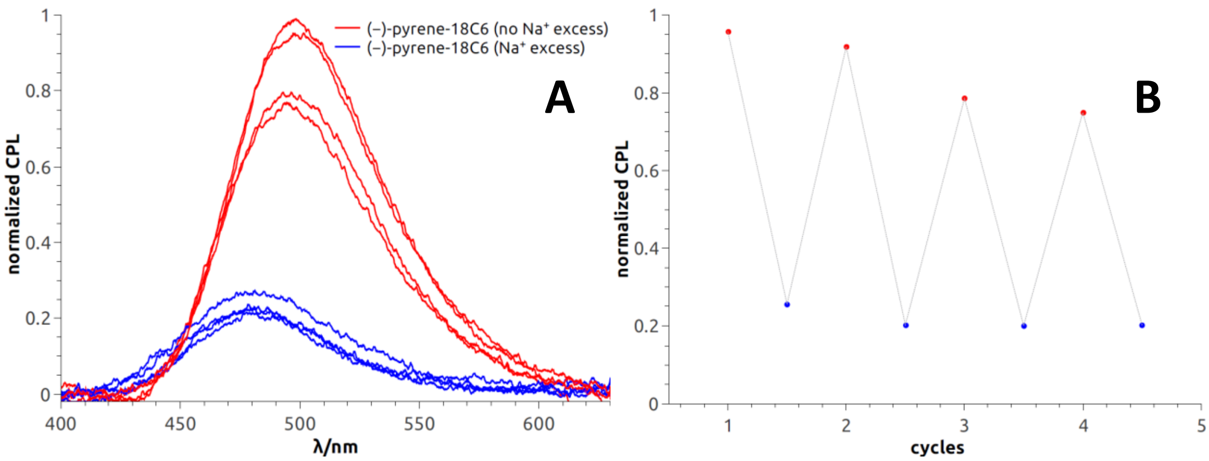

Figure S34. Reversible (normalized) CPL (A) and intensities at 490 nm (B) for the first eluted (−)-enantiomer of **pyrene-18C6** over 4 cycles of $NaBAr_F$/18-Crown-6 additions (red/blue: without/with $NaBAr_F$).

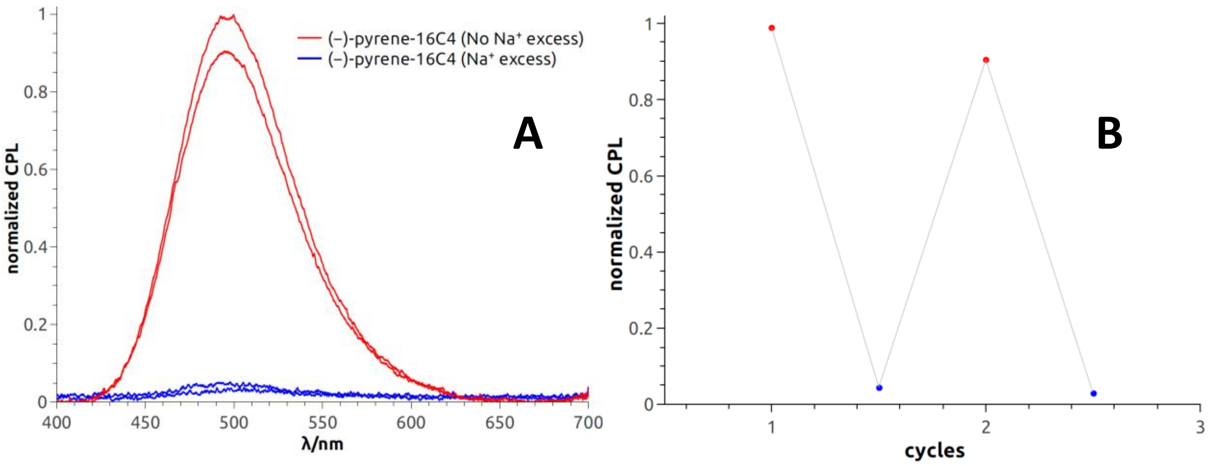

Figure S35. Reversible (normalized) CPL (A) and intensities at 491 nm (B) for the first eluted (−)-enantiomer of **pyrene-16C4** over 2 cycles of $NaBAr_F$/18-Crown-6 additions (red/blue: without/with $NaBAr_F$).

5. Computational Section

5.1. General remarks

All calculations were run with Gaussian'09 and Gaussian'16 suites of programs using defaults grids and convergence criteria.[7] The input geometry of all compounds was built starting from the X-ray structure of **pyrene-18C6** with (*S,S*) configuration bound to one water molecule. The conformational space was explored by varying manually the macrocycle–C(=O) and *N*–aryl bonds, and optimizing the resulting structures with DFT at M06-2X-D3/def2-SVP level,[8] including the IEF-PCM implicit solvent model for dichloromethane.[9] All converged structures displayed a clear π-stacking interaction between the aromatic rings. TDDFT calculations were run with different combinations of functionals (ωB97X-D, M06-2X, B3LYP, CAM-B3LYP), basis sets (def2-SVP, def2-TZVP) and environment description (in vacuo or with IEF-PCM solvent model for dichloromethane). The number of roots (excited states) varied from 50 to 80 depending on the specific compound. The calculated spectra shown in Figures S36 and S37 were obtained with M06-2X and CAM-B3LYP functionals, which yielded the best agreement with the experimental spectra. ECD plots were generated from Gaussian log files using the program SpecDis.[10] The plotting parameters were chosen on a best-fitting basis, and are listed in the caption of Figure S37.

5.2. Calculation results

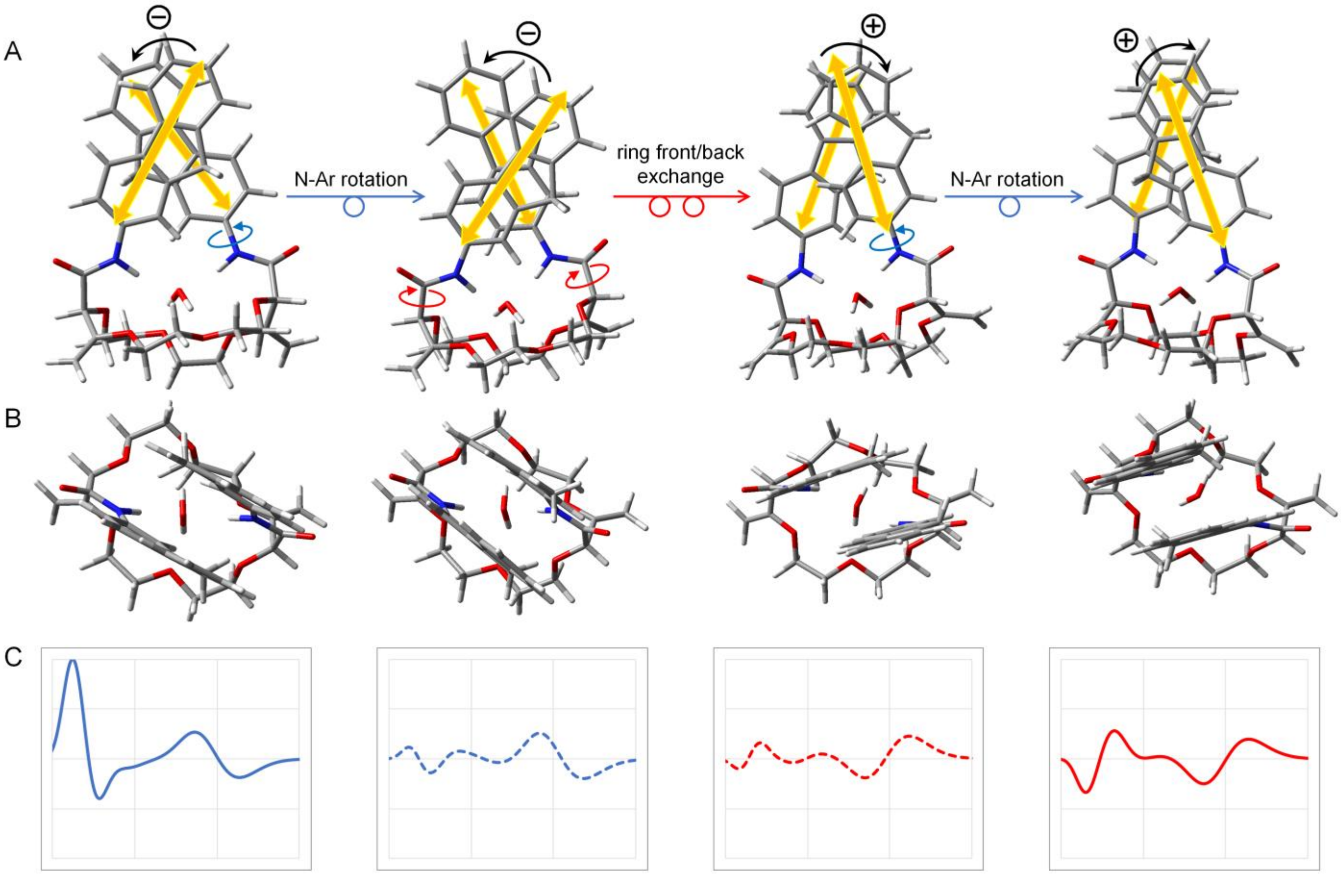

Figure S36. (A,B) Structures of (*S*,*S*)-**fluorene-18C6** optimized with DFT at the M06-2X-D3/def2-SVP/PCM level after rotation around the *N*–aryl and macrocycle–C(=O) bonds. A and B offer two different viewpoints of the same structures. The yellow double arrows depict the direction of the transition moments for the π-π* transition around 280 nm, and the signed curved arrows the chirality defined by the moments. (C) ECD spectra calculated for each structure at CAM-B3LYP/def2-SVP/PCM level. The horizontal and vertical ranges are 190-340 nm and –400-+400, respectively. Plotting parameters: σ = 0.3 eV, wavelength shift +15 nm.

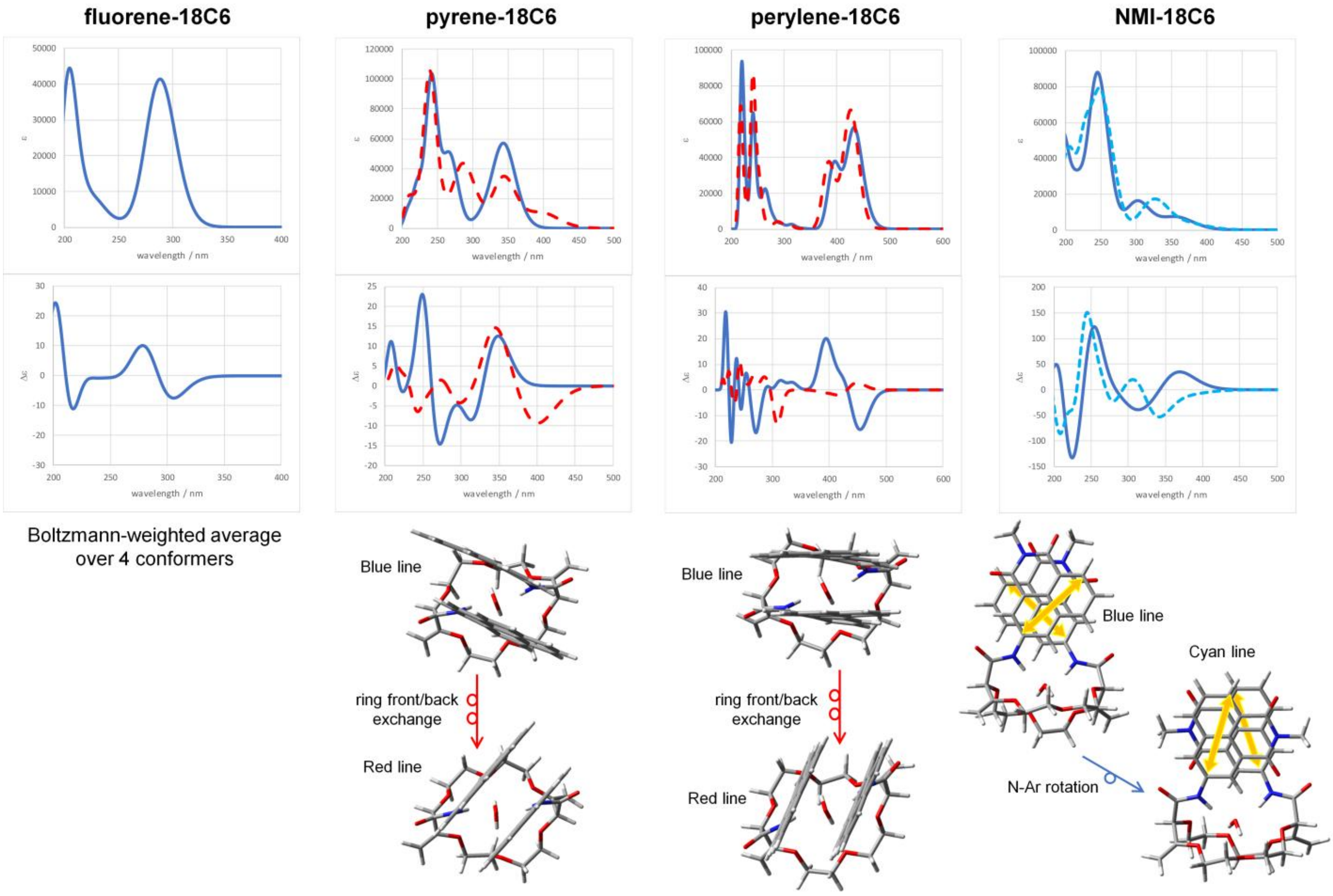

Figure S37. Summary of ECD calculation results for the (*S*,*S*)**-18C6** series. Calculation levels and plotting parameters: **fluorene-18C6**, see Figure S36, scaling factor 10; **pyrene-18C6**, M06-2X/def2-TZVP, σ = 0.25 eV, shift +12 nm, scaling factor 10; **perylene-18C6**, CAM-B3LYP/def2-SVP, σ = 0.15 eV, shift +20 nm, scaling factor 10; **NMI-18C6**, CAM-B3LYP/def2-TZVP, σ = 0.3 eV, no shift or scaling. The yellow double arrows depict the direction of the naphthalene long axes of NMI chromophore. See text explanation.

Because of the structural complexity and flexibility of the **18C6**-based macrocycles, ECD calculations were mainly intended for investigating the dependence of ECD spectra on the key structural parameters of the macrocycles.

In Figure S36, four structures of (*S,S*)-**fluorene**-**18C6** are reported which were obtained upon rotation around the *N*–aryl and macrocycle–C(=O) bonds. All structures feature a pair of stacked fluorene rings with clear chirality defined by their long axes, i.e. the direction along which the main π-π* transition around 280 nm is polarized. Accordingly, the respective calculated ECD spectra display strong ECD couplets, whose sign depends on the chirality defined by the transition moments. It is apparent that the modification the reciprocal arrangement of the fluorene rings (i.e., exchanging the fluorene in the front with that in the back) causes a sign reversal in the ECD couplet. This rearrangement is caused by concerted rotations around the macrocycle–C(=O) bonds.

While differing in sign and intensity, the four spectra are similar to each other in overall shape and position of bands. The weighted average of the four spectra (obtained using computed internal energies) is shown in Figure S37 (left). It consists in a negative ECD couplet, which is in agreement with experimental spectrum for the 1st eluted enantiomer (Figure 4C in the main text). The vibrational fine structure in the experimental spectrum cannot be predicted at the current level of calculation, which does not include vibronic effects.

For **pyrene-** and **perylene-18C6** we investigated the impact of switching the ring position on the ECD spectra (Figure S37, two middle columns). For (*S,S*)-**perylene-18C6** the situation is similar to **fluorene-18C6**: exchanging the ring position from front to back causes a sign reversal in the ECD couplet, which is also accompanied by a strong intensity decrease. The overall ECD profile consists in a negative ECD couplet, as experimentally found for the 2nd eluted enantiomer. The situation for **pyrene-18C6** is more complicated because the two arrangements – obtained after the rings exchange their positions – are associated with very different ECD profiles. The structure attained from re-optimization of the X-ray geometry is associated with a negative ECD couplet; the second structure with exchanged ring positions is associated instead with a red-shifted positive couplet (Figure S37). If one assumes that the first conformation is dominant in solution, the combination of the two would yield an overall spectrum similar to that observed for the 2nd eluted enantiomer (Figure 4A, main text), namely a positive ECD band at 350 nm (mainly due to the major conformer), flanked by a long-wavelength weak negative band (due to the minor conformer).

Finally, for **NMI**-**18C6** the rotamerism around the *N*-aryl bond has a pronounced impact on the orientation of the transition moments, polarized either along the chromophore long or short axis (see yellow double arrows in Figure S37). Therefore, we focused our attention on this degree of conformational freedom without switching the reciprocal position of the rings. The two different arrangements (obtained upon 180°-rotation around the *N*-aryl bonds) display ECD spectra which are almost the mirror image of each other long wavelengths, while having consistent shape and sign at shorter wavelengths (Figure S37, right). Assuming a coexistence of both structures in solution, this would lead to a signal cancelation at long wavelengths, yielding a positive exciton couplet at short wavelengths. The overall result resembles the ECD measured for the 2nd eluted enantiomer (Figure 4D, main text).

6. Crystallographic Data

CCDC number	1045593	
Empirical formula	C48 H42 N2 O6	
Formula weight	742.83	
Temperature	180.05(10) K	
Wavelength	1.54184 Å	
Crystal system	Triclinic	
Space group	P-1	
Unit cell dimensions	a = 11.8363(4) Å	α= 108.968(3)°
	b = 12.6884(4) Å	β= 104.837(3)°
	c = 14.8869(5) Å	γ = 104.872(3)°
Volume	1897.34(13) Å^3	
Z	2	
Density (calculated)	1.300 Mg/m^3	
Absorption coefficient	0.687 mm^{-1}	
F(000)	784	
Crystal size	0.3412 x 0.1328 x 0.0883 mm^3	
Theta range for data collection	3.381 to 72.598°.	
Index ranges	-14<=h<=12, -13<=k<=15, -18<=l<=15	
Reflections collected	12970	
Independent reflections	7288 [R(int) = 0.0177]	
Completeness to theta = 67.000°	99.8 %	
Absorption correction	Semi-empirical from equivalents	
Max. and min. transmission	1.00000 and 0.91638	
Refinement method	Full-matrix least-squares on F^2	
Data / restraints / parameters	7288 / 0 / 513	
Goodness-of-fit on F^2	1.023	
Final R indices [I>2sigma(I)]	R1 = 0.0352, wR2 = 0.0888	
R indices (all data)	R1 = 0.0450, wR2 = 0.0954	
Extinction coefficient	n/a	
Largest diff. peak and hole	0.187 and -0.183 e.Å^{-3}	

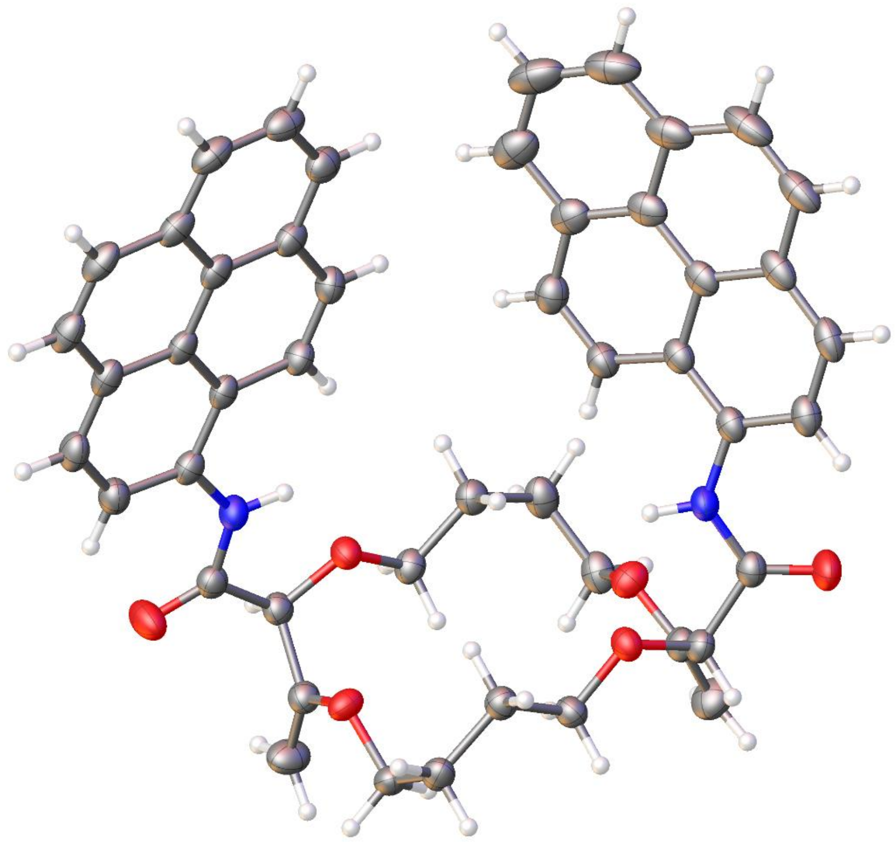

Figure S38. View of the asymmetric unit. Displacement ellipsoids are depicted at 50 percent probability level.

7. References

1 F. Zinna, T. Bruhn, C. A. Guido, J. Ahrens, M. Bröring, L. Di Bari and G. Pescitelli, *Chem. Eur. J*, 2016, **22**, 16089-16098.

2 (a) J. J. Looker, *J. Org. Chem.*, 1972, **37**, 3379-3381; (b) N. Almonasy, M. Nepraš, Š. Hyková, A. Lyčka, J. Čermák, M. Dvořák and M. Michl, *Dyes Pigm.*, 2009, **82**, 164-170.

3 A. Khosravi, S. Moradian, K. Gharanjig and F. Afshar Taromi, *Dyes Pigm.*, 2006, **69**, 79-92.

4 D. Poggiali, A. Homberg, T. Lathion, C. Piguet and J. Lacour, *ACS Catal.*, 2016, **6**, 4877-4881.

5 The crude solid is dissolved in CH_2Cl_2 (*ca.* 5 mL) and into the flask ca. 1.0 g of neutral alumina were added. The slurry was concentrated in vaccuo to yield the solid deposit.

6 Z. Jarolímová, M. Vishe, J. Lacour and E. Bakker, *Chem. Sci.*, 2016, **7**, 525-533.

7 (a) M. J. Frisch, G. W. Trucks, H. B. Schlegel, G. E. Scuseria, M. A. Robb, J. R. Cheeseman, G. Scalmani, V. Barone, B. Mennucci, G. A. Petersson, H. Nakatsuji, M. Caricato, X. Li, H. P. Hratchian, A. F. Izmaylov, J. Bloino, G. Zheng, J. L. Sonnemberg, M. Hada, M. Ehara, K. Toyota, R. Fukuda, J. Hasegawa, M. Ishida, T. Nakajima, Y. Honda, O. Kitao, H. Nakai, T. Vreven, J. Montgomery, J. A., J. E. Peralta, F. Ogliaro, M. Bearpark, J. J. Heyd, E. Brothers, K. N. Kudin, V. N. Staroverov, R. Kobayashi, J. Normand, K. Raghavachari, A. Rendell, J. C. Burant, S. S. Iyengar, J. Tomasi, M. Cossi, N. Rega, J. M. Millam, M. Klene, J. E. Knox, J. B. Cross, V. Bakken, C. Adamo, J. Jaramillo, R. Gomperts, R. E. Stratmann, O. Yazyev, A. J. Austin, R. Cammi, C. Pomelli, J. W. Ochterski, R. L. Martin, K. Morokuma, V. G. Zakrzewski, G. A. Voth, P. Salvador, J. J. Dannenberg, S. Dapprich, A. D. Daniels, O. Farkas, J. B. Foresman, J. V. Ortiz, J. Cioslowski and D. J. Fox, Gaussian, Inc., Wallingford CT, 2013; (b) M. J. Frisch, G. W. Trucks, H. B. Schlegel, G. E. Scuseria, M. A. Robb, J. R. Cheeseman, G. Scalmani, V. Barone, G. A. Petersson, H. Nakatsuji, X. Li, M. Caricato, A. V. Marenich, J. Bloino, B. G. Janesko, R. Gomperts, B. Mennucci, H. P. Hratchian, J. V. Ortiz, A. F. Izmaylov, J. L. Sonnemberg, D. Williams-Young, F. Ding, F. LIpparini, F. Egidi, J. Goings, B. Peng, A. Petrone, T. Henderson, D. Ranasinghe, V. G. Zakrzewski, J. Gao, N. Rega, G. Zheng, W. Liang, M. Hada, M. Ehara, K. Toyota, R. Fukuda, J. Hasegawa, M. Ishida, T. Nakajima, Y. Honda, O. Kitao, H. Nakai, T. Vreven, K. Throssell, J. Montgomery, J. A., J. E. Peralta, F. Ogliaro, M. Bearpark, J. J. Heyd, E. Brothers, K. N. Kudin, V. N. Staroverov, T. A. Keith, R. Kobayashi, J. Normand, K. Raghavachari, A. Rendell, J. C. Burant, S. S. Iyengar, J. Tomasi, M. Cossi, J. M. Millam, M. Klene, C. Adamo, R. Cammi, J. W. Ochterski, R. L. Martin, K. Morokuma, O. Farkas, J. B. Foresman and D. J. Fox, Gaussian, Inc., Wallingford CT, 2016.

8 (a) S. Grimme, J. Antony, S. Ehrlich and H. Krieg, *J. Chem. Phys.*, 2010, **132**, 154104; (b) Y. Zhao and D. G. Truhlar, *Theor. Chem. Acc.*, 2008, **120**, 215-241.

9 B. Mennucci and R. Cammi, *Continuum Solvation Models in Chemical Physics: From Theory to Applications.*, Wiley, New York, 2008.

10 T. Bruhn, A. Schaumlöffel, Y. Hemberger and G. Bringmann, *Chirality*, 2013, **25**, 243-249.
